# First-in-Class Covalent Inhibitors of PFKFB3: Discovery and Characterization in PDAC Models

**DOI:** 10.1021/acs.jmedchem.6c00235

**Published:** 2026-05-26

**Authors:** Alessandra Fiore, Antonio Scarano, Giulia Antonini, Alexandra Ioana Corfù, Lea Sicuro, Serena Faggiano, Adriana Celesia, Chiara Tesoriero, Raffaella Pacchiana, Andrea Vettori, Liaisan Arslanbaeva, Saverio Minucci, Isabella Pallavicini, Luca Mollica, Lucia Tamborini, Massimo Donadelli, Paola Conti, Stefano Bruno, Chiara Borsari

**Affiliations:** 1 Department of Neurosciences, Biomedicine and Movement Sciences, Section of Biochemistry, 19051 University of Verona, 37134 Verona, Italy; 2 Department of Food and Drug, University of Parma, Parco Area delle Scienze 23/a, 43124 Parma, Italy; 3 Department of Pharmaceutical Sciences, 9304 University of Milan, Via Mangiagalli 25, 20133 Milan, Italy; 4 Department of Medical Biotechnologies and Translational Medicine c/o L.I.T.A/ 9304 University of Milan, Via F.lli Cervi 93, 20090 Segrate (MI), Italy; 5 Fondazione IRCCS Ca’Granda Ospedale Maggiore Policlinico, Angelo Bianchi Bonomi Hemophilia and Thrombosis Center, 20133 Milan, Italy; 6 Institute of Biophysics, National Research Council (CNR), Via G. Moruzzi 1, 56124 Pisa, Italy; 7 Department of Biotechnology, 19051 University of Verona, 37134 Verona, Italy; 8 Department of Experimental Oncology, IEO European Institute of Oncology IRCSS, 20139 Milan, Italy; 9 Department of Oncology and Hematology-Oncology, 9304 University of Milan, 20122 Milan, Italy

## Abstract

Pancreatic ductal adenocarcinoma (PDAC) is an aggressive cancer, driven by metabolic reprogramming. Since direct glycolytic enzyme inhibition is limited by toxicity, indirect glycolysis modulation through inhibition of the kinase activity of 6-phosphofructo-2-kinase/fructose-2,6-bisphosphatase 3 (PFKFB3) may offer a safer therapeutic strategy. Herein, we report the first-in-class covalent PFKFB3 inhibitor (**6**), targeting a previously unexplored cysteine. Enzyme assays, site-directed mutagenesis, and mass spectrometry confirmed covalent binding and kinetic selectivity for PFKFB3. Compound **6** reduced viability across multiple PDAC cell lines and suppressed PDAC growth in zebrafish xenografts. Its combination with standard chemotherapeutics revealed synergistic effects. Although the limited cellular activity of **6** restricts its use as a chemical probe in biological studies, we proved for the first time the druggability of a previously unexplored cysteine in PFKFB3. Our work represents a significant achievement in the selective targeting of this kinase, paving the way for an innovative mechanism of action for PFKFB3 inhibitors.

## Graphical abstract

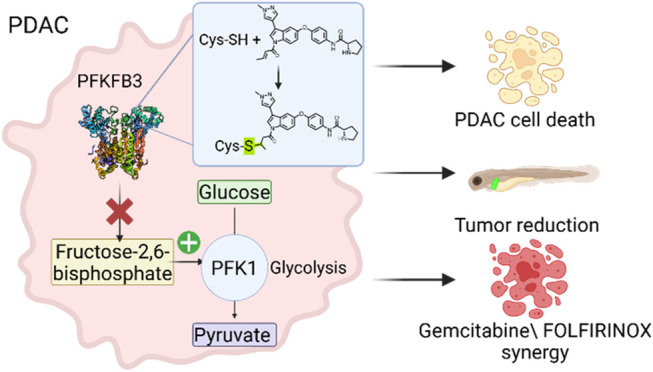

## Introduction

Pancreatic ductal adenocarcinoma (PDAC) is a highly aggressive malignancy with poor prognosis and rising incidence. PDAC accounts for 90–95% of all pancreatic tumors and the 5-year overall survival rate is only 11%. Approximately 80% of pancreatic cancer patients present with advanced disease or distant metastases and have no effective treatment options. The difficulty in diagnosing and assessing PDAC at an early stage, together with its particularly aggressive biology and limited responsiveness to standard chemotherapeutic treatments, are major therapeutic challenges.^1–3^ In particular, chemoresistance is a key impediment to treating PDAC.^4,5^ Therefore, drugs with innovative mechanisms of action could help overcome resistance to existing therapies.^6^

Pancreatic cancer cells reprogram their metabolism and enhance anaerobic glycolysis, regardless of oxygen concentration, a phenomenon known as the Warburg effect. This metabolic reprogramming is a hallmark of cancer and is responsible for the malignant behavior and rapid progression of PDAC. Therefore, targeting glycolysis has attracted significant attention in PDAC therapeutic research.^7^ Unfortunately, despite promising preclinical investigations, the inhibition of glycolytic enzymes has not yet been translated into clinical practice, mainly due to systemic toxicity,^8^ suggesting that enzymes indirectly involved in glycolysis regulation may represent more viable therapeutic targets. Phosphofructo-2-kinase/fructose-2,6-bisphosphatases (PFK-2/FBPases-2) catalyze the conversion of fructose-6-phosphate (F6P) to fructose-2,6-bisphosphate (F2,6BP), a potent allosteric activator of phosphofructokinase-1 (PFK-1), which catalyzes one of the rate-limiting steps of glycolysis.^9^ PFK-2/FBPases-2 are bifunctional, also possessing a phosphatase domain that catalyzes the hydrolysis of F2,6BP to F6P. The four PFK-2/FBPase-2 isoforms (PFKFB1–PFKFB4) display tissue-specific expression and different kinase/phosphatase ratios. Among them, PFKFB3 stands out due to its high kinase/phosphatase ratio, which strongly favors F2,6BP synthesis and glycolytic flux, whereas the other isoforms show significantly lower ratios and more balanced activities.^10^ PFKFB3 is expressed almost ubiquitously and is upregulated in response to mitogenic, inflammatory, and hypoxic signals.

In the context of PDAC, PFKFB3 is markedly upregulated in tumor tissues compared to normal pancreatic epithelium. Its overexpression contributes to the pronounced glycolytic dependency characteristic of PDAC cells, supporting rapid proliferation, survival under hypoxic conditions, and resistance to chemotherapy.^11,12^ Inhibition of PFKFB3 in preclinical models has been shown to impair tumor growth, reduce metastatic potential, and sensitize cancer cells to standard treatments such as gemcitabine and radiotherapy.^13–16^ These findings identify PFKFB3 as a promising metabolic vulnerability in PDAC and underscore the therapeutic potential of its pharmacological inhibition, which can selectively disrupt the elevated glycolytic flux in cancer cells while preserving basal glycolysis in healthy tissues. This partial modulation of glucose metabolism, unlike the complete blockade associated with the inhibition of glycolytic enzymes, offers a potentially broader therapeutic window and underscores the relevance of PFKFB3 as an attractive target for transforming PDAC therapy.

Several PFKFB3 inhibitors from different chemical classes have been identified,^17^ with 3PO (**1**, Figure 1a) being the most studied.^18^ Although 3PO initially showed promising results, its poor water solubility, limited selectivity, and the high doses required to achieve therapeutic efficacy have hampered its clinical use. Moreover, a recent study demonstrated that 3PO does not directly bind PFKFB3, and thus its effects on glycolysis are mediated through alternative mechanisms.^19^ Derivatives of 3PO were developed, including PFK15 (**2**, Figure 1a), which showed greater selectivity and potency against PFKFB3 compared to 3PO. In addition, PFK15 exhibited improved pharmacokinetic properties, such as reduced clearance, and demonstrated significant anticancer activity *in vivo*.^17,19^ Other PFKFB3 inhibitors with distinct chemical scaffolds were identified, including a benzopyranone-based molecule (**3**, Figure 1a).^20^ Overall, these claimed selective PFKFB3 inhibitors (**1–3**) show limited structural complexity and could be rather classified as pan-assay interference compounds (PAINS). More recently, an *N*-methylpyrazole derivative (compound **4**, Figure 1b and c) was identified as a potent reversible PFKFB3 inhibitor, showing selectivity for PFKFB3 (IC_50_ = 0.021 μM) over the isoforms PFKFB1 (IC_50_ = 2.35 μM) and PFKFB2 (IC_50_ = 0.818 μM).^21^ Despite the encouraging and promising results achieved over the years, substantial opportunities remain for improving the design and synthesis of novel selective PFKFB3 inhibitors.

**Figure 1:**
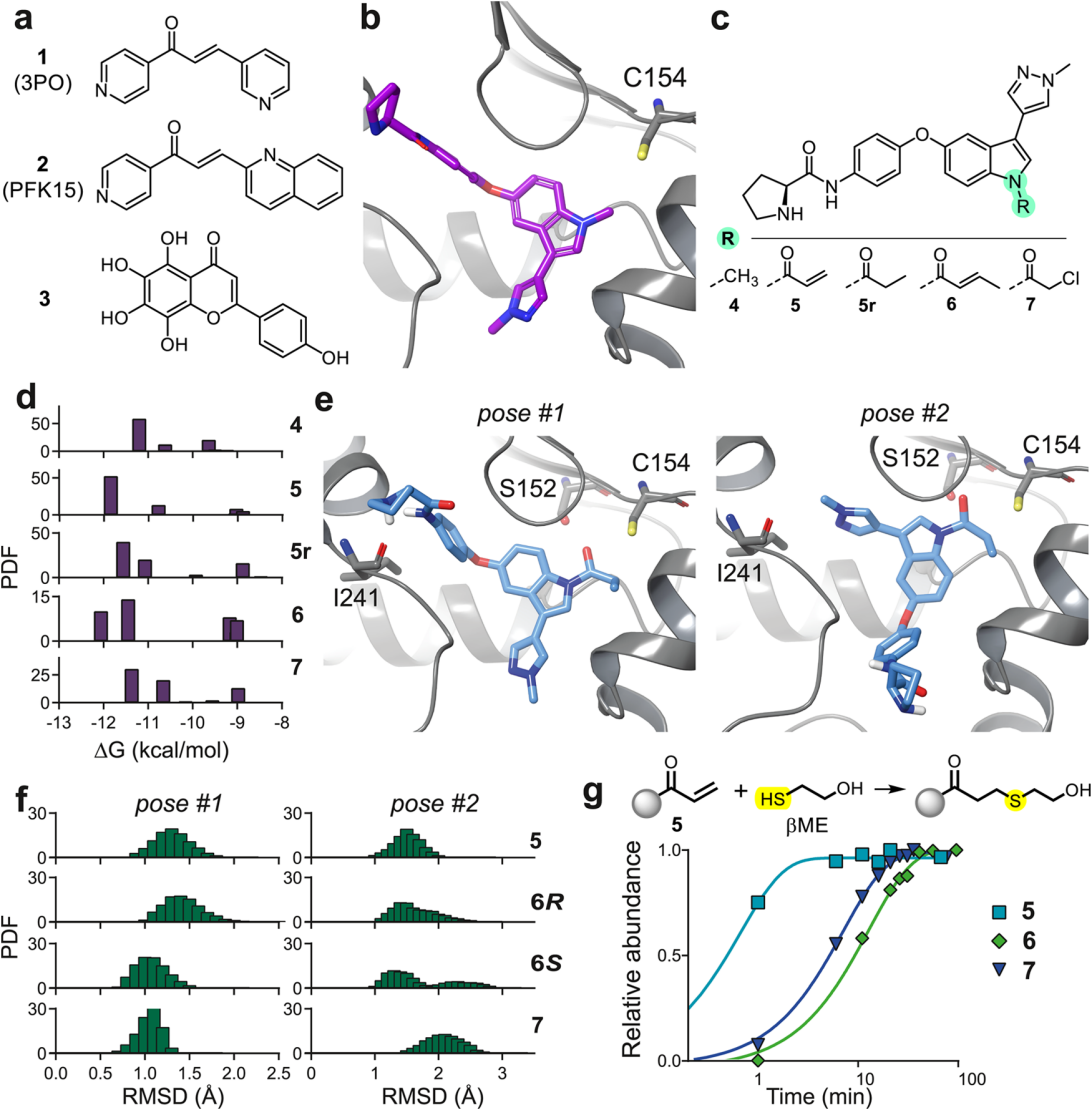
Design and evaluation of covalent PFKFB3 inhibitors. (a) Chemical structure of claimed selective PFKFB3 inhibitors. (b) Crystal structure of **4** in complex with the catalytic domain of PFKFB3 (PDB ID: 5ak0). (c) Chemical structure of ligand **4** and novel inhibitors (**5**–**7**) designed as covalent derivatives of **4**. (d) Statistical distribution of Δ*G* (expressed in kcal·mol^–1^) over 100 docking poses for **4**–**7**. Probability distribution functions (PDF) are expressed in arbitrary units. (e) Representative structures of *pose #1* and *pose #2* for **5**. (f) Root mean square deviation (RMSD, expressed in Å) of the heavy atoms of the covalently bound **5**, **6** *R*-enantiomer and *S*-enantiomer, and **7** for the MD simulations of *pose #1* and *pose #2*. Probability distribution functions (PDF) are expressed in arbitrary units. (g) General reaction of warhead-containing compounds with βME and reactivity of βME (60 mM) with compounds **5–7**, each at 100 μM, incubated at 25 °C in a buffer solution containing 10 mM ammonium acetate at pH 7.0. The *m*/*z* corresponding to the adduct between the compounds and βME (Table S1 in the Supporting Information) was monitored over time by ESI-MS. The intensities were normalized to the end-point value and fitted using a shifted exponential function. Values are mean ± standard deviation (SD) (*n* = 3). Error bars are not shown when smaller than symbols.

Particularly, covalent inhibitors of PFKFB3 could overcome the limitations of reversible molecules. Over the past decade, there has been a renewed interest in compounds containing a reactive chemical moiety, known as “warhead”, capable of forming a covalent bond with the target protein.^22^ Covalent drugs have demonstrated substantial therapeutic benefits across a variety of diseases, owing to their unmatched potency, selectivity, and prolonged duration of action.^22,23^ Notably, several covalent kinase inhibitors have already been approved for oncology applications, including afatinib, ibrutinib, osimertinib, neratinib, acalabrutinib, and dacomitinib.^24–28^ In 2025 alone, four targeted covalent inhibitors of kinases have been approved (zongertinib,^29^ sunvozertinib,^30^ rilzabrutinib,^31^ and remibrutinib^32^) for the treatment of cancer and immune diseases. Beyond the therapeutic applications, covalent molecules are also used as chemical probes to explore the role of enzymes in human diseases.^33–35^ To date, no covalent inhibitors targeting PFKFB3 have been reported.

Herein, we disclose the first-in-class covalent PFKFB3 inhibitors, paving the way for the development of novel, potent and selective PFKFB3 ligands for PDAC treatment.

## Results and Discussion

### Design of Covalent Inhibitors

Mapping of unexplored cysteine residues in PFKFB3 allowed the identification of Cys154, which lies in the ATP-binding site and is in close proximity to the scaffold of recently discovered ATP-competitive reversible inhibitors.^21,36,37^ Starting from the known structure of PFKFB3 in complex with compound **4** (Figure 1b), novel putative covalent ligands were designed by introducing electrophilic warheads into the indole ring of **4**. Considering the distance between the indole ring and the cysteine side chain (approximately 4 Å), acrylamides and α-chloroacetyl groups were selected as electrophiles, yielding compounds **5**–**7** (Figure 1c). These electrophilic moieties are known to covalently react with cysteine side chains and have been previously employed in kinase targeting.^34^ In addition to the putative covalent inhibitors, an appropriate negative control incapable of forming covalent bonds was designed by introducing a propionyl group on the indole ring (**5r**, Figure 1c).

The newly designed covalent inhibitors **5**–**7** (Figure 1c), along with the reversible analog **5r**, were investigated *in silico* through docking calculations and molecular dynamics (MD) simulations prior to synthesis. Compound **4** was also included in these computational studies as positive control, being a known noncovalent ligand of PFKFB3 with the resolved crystal structure, which served as a model for our investigations.^21^ Noncovalent docking calculations were performed and a statistical approach to docking, i.e., based on the generation of several docking poses that included ligand torsional dynamics, led to a distribution of semiempirical binding free energies (Δ*G*, Figure 1d). For all the examined ligands, the most populated binding poses were those with the lowest estimated Δ*G* values. In addition, the distribution of ΔG shows that for compounds **5–7** and **5r** some poses occur with a more favorable binding energy than **4**. For the putative covalent ligands **5**–**7**, we monitored the distances between the Cys154 sulfur and the β-carbon of the warhead (Figure S1 in the Supporting Information), and it turned out to be highly populated around 3 Å, a distance compatible with the formation of the desired covalent bond. Moreover, we performed a detailed analysis of the docking poses and identified two key hydrogen bonds that serve as anchoring points for the ligands within the binding site: one between Ser152 side chain and the carbonyl oxygen of the warhead, and one between Ile241 backbone and the proline NH moiety present in **4** and in its derivatives. Based on the hydrogen-bond network formed by covalent ligands **5**–**7**, and on the position and orientation of the indole core within the binding site, we identified two distinct binding modes, hereafter referred to as *pose #1* and *pose #2* (Figure 1e and Figure S1). In *pose #1*, the ligand almost completely overlaps with the orientation of ligand **4** in the original crystallographic structure (Figure 1b), maintaining hydrogen bonds with both Ser152 and Ile241, and preserving an orientation compatible with covalent bond formation. In contrast, *pose #2* shows the ligand flipped by approximately 180° relative to the original orientation (Figure 1b), resulting in the loss of stabilizing interactions with residue Ile241 (Figure 1e). However, the position of the indole ring, although flipped compared to *pose #1*, still allows sufficient proximity to the cysteine of interest, enabling formation of the covalent bond. To investigate the covalent interaction with the protein, the energetically most favorable docking poses of **5**–**7** were covalently bound to PFKFB3. The resulting covalent complexes were then subjected to 100 ns MD simulations. Compound **6** was simulated as (*R*) and (*S*) enantiomer to investigate the configuration of the chiral center created after the covalent bond formation. The root-mean-square deviation (RMSD) of the entire covalently bound ligand averaged over time revealed a greater stability for *pose #1* with respect to *pose #2*, considering the lower RMSD values obtained for the first binding mode compared with the second (Figure 1f). Overall, all the designed putative covalent ligands (**5**–**7**) could interact with the protein by forming stabilizing interactions *in silico*, allowing stable positioning of the ligand in the binding pocket with an orientation that enables subsequent covalent interactions with the targeted cysteine residue. Moreover, the results indicated that even after covalent bond formation, the ligands are likely to remain stable and well-positioned within the binding pocket, suggesting a persistent occupation of the active site within small geometrical fluctuations around the starting position and orientation.

### Chemistry

Guided by the encouraging *in silico* findings, the designed inhibitors were synthesized through a multistep procedure, starting with the preparation of the common scaffold **13**, followed by its functionalization with the appropriate acylating agent to yield the covalent inhibitors **5**–**7** and the reversible compound **5r** (Scheme 1).

**Scheme 1:**
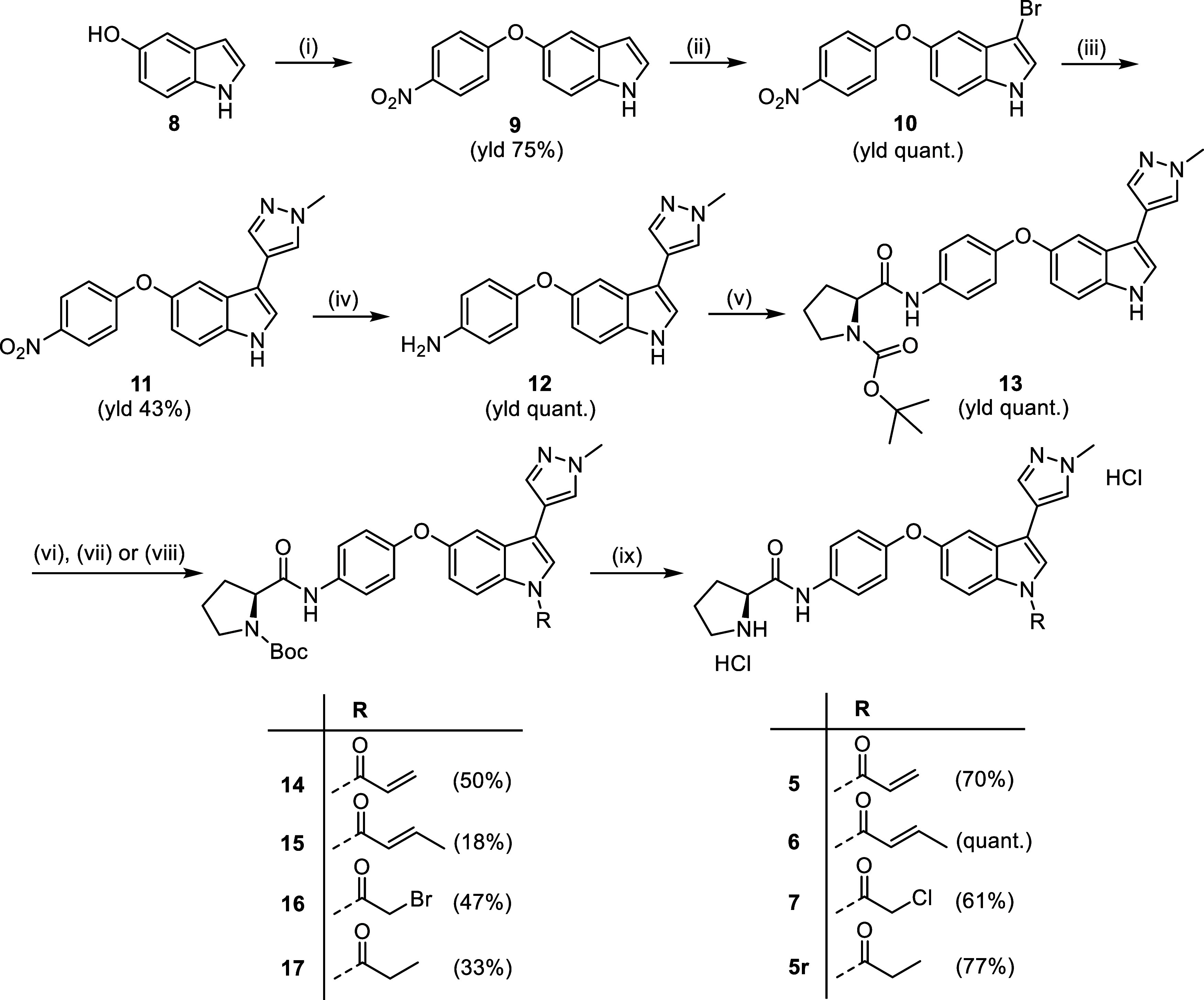
Synthesis of Compounds **5**–**7** and **5r** ^a^

a Reagents and conditions: (i) 1-fluoro-4-nitrobenzene, Cs_2_CO_3_, dry DMF, r.t., o/n; (ii) NBS, dry DMF, r.t., 2 h; (iii) 1-methyl-4-(4,4,5,5-tetramethyl-1,3,2-dioxaborolan-2-yl)­pyrazole, Pd­(PPh_3_)_4_, 1 M K_3_PO_4_, dry 1,4-dioxane, 80 °C, o/n; (iv) H_2_, 10% Pd/C, MeOH, r.t., 3 h; (v) *N*-Boc-*L*-proline, HBTU, DIPEA, dry CH_2_Cl_2_, r.t., 3 h; (vi) acryloyl chloride or propionyl chloride, Cs_2_CO_3_, dry DMF, r.t., o/n (for **14** and **17**); (vii) crotonyl chloride, NaOH, tetrabutylammonium hydrogen sulfate (TBAHS), dry CH_2_Cl_2_, r.t., 3 h (for **15**); (viii) bromoacetyl bromide, DBU, dry CH_2_Cl_2_, r.t., 1.5 h (for **16**); (ix) acetyl chloride, dry MeOH, r.t., 1 h.

The synthetic route, inspired by the previously reported^21^ synthesis of compound **4**, began with a nucleophilic aromatic substitution using commercially available 1-fluoro-4-nitrobenzene and **8**, affording intermediate **9** in good yield. Bromination at position 3 of the indole ring was then carried out using *N*-bromosuccinimide, yielding compound **10** in quantitative yield. Compound **10** was subsequently subjected to a Suzuki cross-coupling reaction with 1-methyl-4-(4,4,5,5-tetramethyl-1,3,2-dioxaborolan-2-yl)­pyrazole, using K_3_PO_4_ as base and tetrakis­(triphenylphosphine)­palladium(0) as catalyst, to give compound **11** in a moderate yield (43%). The nitro group of **11** was then reduced via catalytic hydrogenation using 10% palladium on activated carbon, providing the corresponding aniline derivative **12** in quantitative yield. The following step involved an amide coupling between **12** and *N*-Boc-*L*-proline, employing HBTU and *N*,*N*-diisopropylethylamine (DIPEA), to afford the scaffold **13** in excellent yield. Afterward, the indole NH-group of **13** was acylated with the appropriate acylating agents. Intermediate **14** and its corresponding reversible molecule **17** were obtained in moderate yields (50 and 33%, respectively) by acylation of scaffold **13** using acryloyl chloride or propionyl chloride in the presence of Cs_2_CO_3_ as base. Derivative **15** was synthesized in modest yield using crotonyl chloride as acylating agent, finely powdered NaOH as base and a catalytic amount of tetrabutylammonium hydrogen sulfate (TBAHS).^38^ These modified conditions were explored due to the low reactivity observed under the standard conditions (Cs_2_CO_3_ as base) employed for the preparation of **14** and **17**. The brominated derivative **16** was synthesized in moderate yield using bromoacetyl bromide in the presence of DBU. Initial attempts employing chloroacetyl chloride to obtain the corresponding chlorinated acylated product resulted in unsatisfactory yields. This outcome was attributed to the low reactivity of the electrophile; therefore, bromoacetyl bromide was selected as a more reactive alternative. The final step involved removal of the Boc-protecting group on the proline moiety, carried out under acidic conditions using methanolic hydrochloric acid generated *in situ* from acetyl chloride. This step afforded the desired products (**5**–**7** and **5r**) as dihydrochloride salts in good to excellent yields. Notably, application of this deprotection protocol to compound **16** resulted in efficient Boc deprotection accompanied by concomitant halogen exchange, ultimately yielding the target compound **7** bearing the α-chloroacetyl electrophilic warhead.

Given the modest yields observed during the functionalization of the indole core with various acylating agents (Scheme 1), we sought to optimize this transformation by leveraging flow chemistry technology. Intermediates **15** and **17**, showing the lowest yield in batch, were selected as proof-of-concept substrates to develop an appropriate continuous-flow procedure, using a reactor packed with a solid or immobilized base. A first flow experiment was performed employing a glass column packed with finely ground NaOH as the heterogeneous base. A solution of indole **13** containing the phase-transfer catalyst TBAHS was mixed in a T-piece with a solution of crotonyl chloride (Scheme 2a). At room temperature and 15 min residence time, complete consumption of the starting material was observed by TLC. After aqueous workup, followed by flash chromatography, the acylated product was isolated in 24% yield. Although the improvement of the yield compared to the batch conditions was modest (24% vs 18%), we significantly reduced the reaction time (15 min vs 3 h). Unfortunately, a gradual increase in system pressure was observed during the reaction, consistent with potential bed compaction, channeling, or partial blockage in the NaOH-packed column. To address this issue, the base was switched from powdered NaOH to Amberlite 900 (^−^OH), prepared from the commercially available Amberlite 900 (Cl^–^) via ion-exchange. The column was packed with the resin and conditioned with dry CH_2_Cl_2_. Then, the reaction was performed using the same conditions described above. Standard workup followed by column chromatography afforded the desired product **15** in good yield (56%). Moreover, an in-line workup was added to reduce working time and manual handling. An inlet of aqueous 1 M NaOH was added followed by a 2 mL reactor coil and a liquid–liquid separator (Scheme 2b). The collected organic phase was then dried over anhydrous Na_2_SO_4_ and evaporated to obtain the crude product. The same reaction was performed using propionyl chloride as acylating agent to obtain the desired intermediate **17** with >95% purity and in 45% yield. These results show that continuous-flow acylation of indole intermediate **13** using an immobilized hydroxide base (Amberlite 900 (^−^OH)) significantly enhances reaction performance, yielding product **15** and **17** in 56 and 45% yield, respectively, in only 15 min of residence time. Moreover, a lower boiling point solvent was used (CH_2_Cl_2_ instead of DMF), and an in-line workup was introduced to further streamline isolation and improve throughput.

**Scheme 2:**
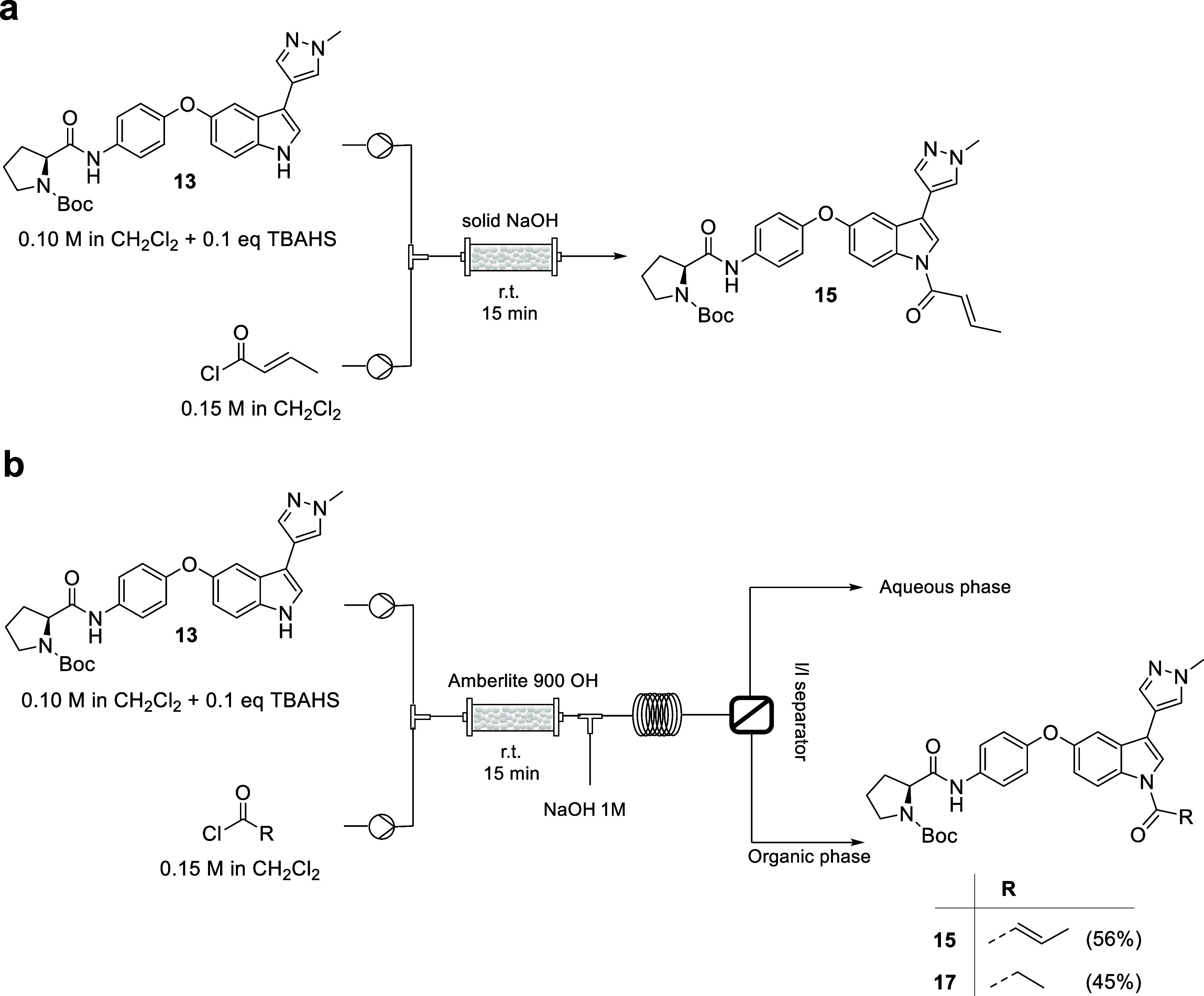
Continuous Flow Synthesis of Intermediate **15** and **17** Using (a) NaOH-Packed Column or (b) Amberlite 900 (^−^OH)-Packed Column with Integrated Work Up

### Assessment of Intrinsic Warhead Reactivity

To evaluate the reactivity of the compounds designed to covalently target cysteine residues, we incubated compounds **5**–**7** with the model thiol β-mercaptoethanol (βME),^34,39^ followed by time-resolved analysis of the adducts via electrospray ionization mass spectrometry (ESI-MS). Both the intact compounds and their covalent adducts with βME were observed at the expected mass-to-charge ratios (Table S1 in the Supporting Information). Peak intensities, normalized to the maximum intensity of the time course, showed a progressive decrease of the unreacted compounds and a corresponding increase of the βME adducts (Figure 1g and Figure S2). From these data, pseudo first-order rate constants were determined and converted into second-order rate constants, yielding the following values: *k*_chem_ (**5**) = 0.35 M^–1^·s ^–1^, *k*_chem_ (**6**) = 0.020 M^–1^·s^–1^, *k*_chem_ (**7**) = 0.035 M^–1^·s^–1^. Overall, **6** displayed a second-order rate constant 17.5-fold and 1.75-fold lower than those of **5** and **7**, respectively, suggesting reduced potential for off-target binding and improved drug-like properties. In comparison, ibrutinib, with a *k*_chem_ of 1.42·10^–4^ M^–1^·s^–1^, exhibited a 140-fold slower reactivity in comparison to **6**. However, higher reactivity may be advantageous in early stage probe discovery.

### Inhibitory Activity toward PFKFB3 and the C154S Variant

To assess the binding of our novel compounds to their target protein, we recombinantly expressed PFKFB3 (Figure S3a). A C154S variant was also expressed, with the dual purpose of clarifying the biochemical role of the target cysteine and serving as a control variant incapable of forming covalent bonds through Cys154. In SEC analysis, PFKFB3 showed an apparent molecular weight (MW) of 114,600 Da, consistent with a dimer (expected MW: 120,600 Da), as reported for the nonphosphorylated form of the enzyme^40^ (Figure S3b). A continuous enzyme assay based on pyruvate kinase (PK) and lactate dehydrogenase (LDH), reported for other kinases^41^ but not for PFKFBs, was first adapted to PFKFB3 by adjusting substrate concentrations, pH, and temperature conditions to maximize linearity. Since phosphoenolpyruvate (PEP) was reported to inhibit PFKFBs,^42^ a discontinuous version of the assay with PEP added only in the detection phase was tested. Reaction rates differed less than 5% from the continuous assay, showing negligible PEP interference under our conditions (Figure S3c). Therefore, this novel continuous assay was used to confirm the kinetic parameters of PFKFBs^43^ and assess those for the C154S variant (Figure S3d and Table S2) at 2.5 μM concentration.

The kinetic comparison between PFKFB3 and its C154S variant revealed modest changes: although the mutation approximately doubled the *K*_m_ for both ATP (151 → 272 μM) and F6P (331 → 643 μM), the *k*_cat_ also doubled. As a result, the overall catalytic efficiencies (*k*_cat_/*K*_m_) remained essentially unchanged for both substrates. These results suggested that Cys154 plays a limited but measurable role in modulating substrate binding, likely by influencing the active site environment or local flexibility.

Prior to evaluating covalent inhibition, we performed enzyme assays to confirm the binding of **4**—reported to display an IC_50_ of ∼0.02 μM for PFKFB3 in cellular assays^21^—and to assess the binding of the putative reversible inhibitor **5r**. For both molecules, the IC_50_ was determined to be 0.15 μM, corresponding to *K*_*i*_s of 0.02 μM, calculated using eq 1 to account for substrate concentration in the assay (Table 1 and Figure S4a). Therefore, the introduction of bulky substituents on scaffold **4** preserved PFKFB3 binding affinity at nanomolar levels. C154S PFKFB3 exhibited *K*_*i*_s of 0.13 μM and 0.009 μM for **4** and **5r**, respectively (Table 1 and Figure S4a), supporting the use of the C154S variant as a tool to study the binding of the covalent inhibitors targeting Cys154.

**Table 1:** Ligand Binding Parameters of PFKFB3 and C154S PFKFB3 with **4**, **5r**, and **6**

	**4**	**5r**	**6**			
	*K*_*i*_ (μM)	*K*_*i*_ (μM)	*K*_*i*_ (μM)	*k*_inact_ (s^–1^)	*K*_*I*_ (μM)	*k*_inact_/*K*_*I*_ (M^–1^·s^–1^)
PFKFB3	0.02 ± 0.01	0.02 ± 0.01	-	0.018 ± 0.002	11.3 ± 2.1	(1.62 ± 0.14) × 10^3^
C154S PFKFB3	0.13 ± 0.07	0.009 ± 0.002	0.09 ± 0.02	-	-	-

### Evaluation of Covalent Binding

Compounds **5**–**7**, structurally derived from **4** and containing a thiol-reactive moiety, were tested as inhibitors of PFKFB3 using the coupled assay. All compounds almost completely inhibited the enzyme at 5 μM concentration after 30 min (Figure 2a). Inhibition assays were then performed at inhibitor concentrations below that of the enzyme. Protein-to-ligands ratios ranging between 1 and 1.7 were measured, indicating a near stoichiometric inhibition (Figure 2b). To rule out any interference of the chemically reactive compounds with the coupled enzymes of the assay, we followed the inhibition with an NMR assay that directly monitors ADP formation and ATP consumption (Figure 2c). In the experiment with PFKFB3 alone, the peak of the purine H8 proton shifted from 8.395 to 8.402 ppm, indicating the conversion of ATP to ADP over time (Figure S4b). The presence of the compounds reduced the rate of formation of ADP from ATP, thus confirming their inhibitory effect on PFKFB3.

**Figure 2:**
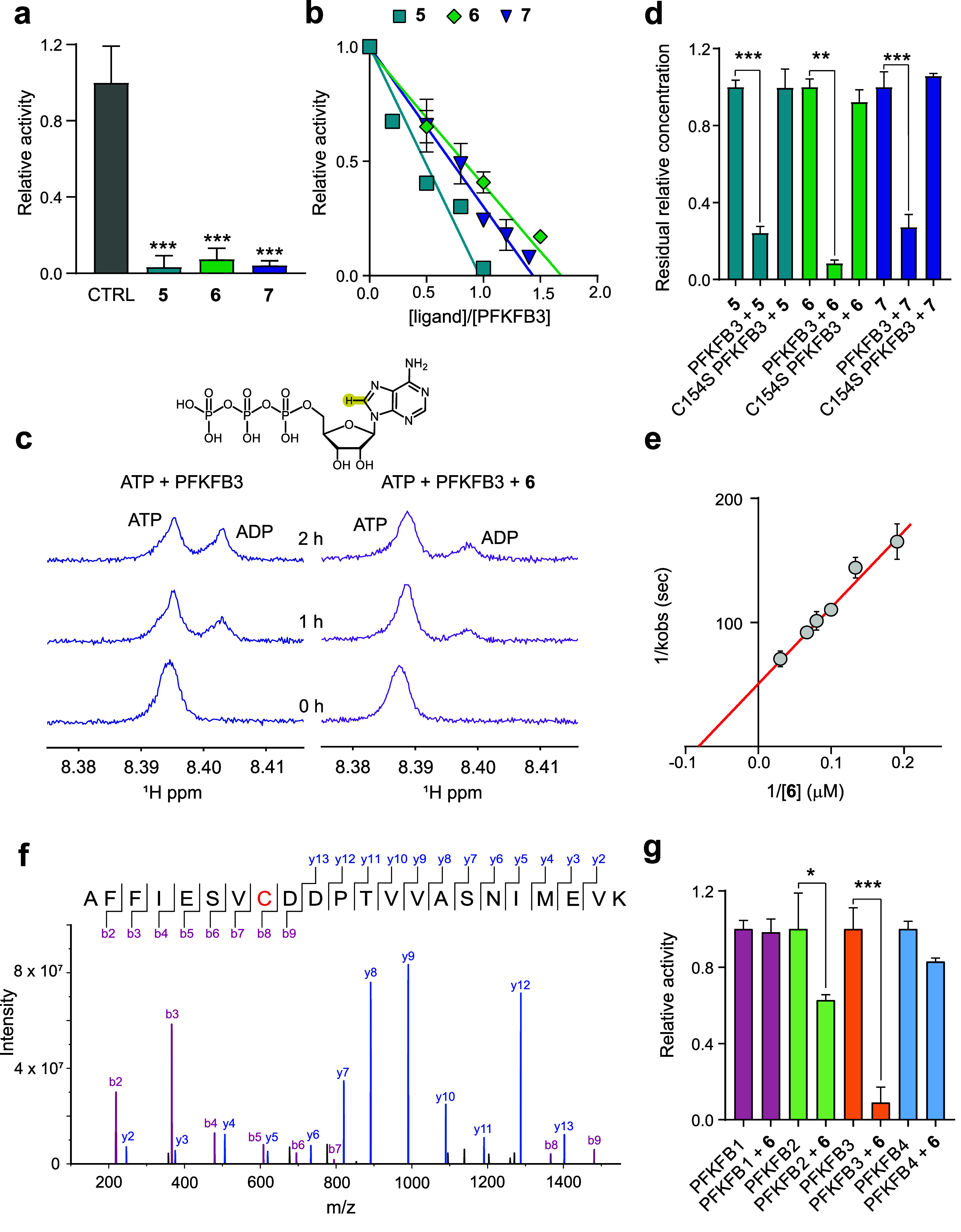
Binding of covalent inhibitors to PFKFB3. (a) Activity of PFKFB3 after incubation with compounds **5**–**7** (5 μM, 30 min). Enzyme concentration was 2.5 μM. Data are plotted as mean of three independent replicates ± SEM. Error bars are not shown when smaller than the symbols. Statistical analysis was performed using a paired two-tailed *t* test (***p < 0.001). (b) Inhibition of PFKFB3 (2.5 μM) by **5** (black), **6** (blue), and **7** (red) at different inhibitor concentrations below that of the enzyme (2.5 μM). Residual enzymatic activity is expressed as a fraction of the untreated control. Data are plotted as mean of three independent replicates ± SEM. Error bars are not shown when smaller than the symbols. (c) NMR spe**c**tra of the reaction mixture (ATP 200 μM initial concentration, F6P 2 mM, PFKFB3 2.5 μM) recorded in the absence (left) and presence (right) of **6** (12.5 μM) at three time points. (d) Binding of **5**, **6**, and **7** to PFKFB3 and the C154S variant assessed by TFA-induced coprecipitation. Proteins (25 μM) were incubated with each compound (16.5 μM) at 25 °C, followed by TFA addition and centrifugation. The absorbance of the supernatant was measured at the compound-specific λ_max_ and normalized to that of the compound subjected to mock TFA treatment (no protein). Data are plotted as mean of three independent replicates ± SEM. Statistical analysis was performed using a paired two-tailed *t* test (**p < 0.01, ***p < 0.001). (e) Kitz–Wilson analysis of **6**. The plot shows the relationship between inhibitor concentration and inactivation rate, enabling the determination of *k*_inact_ and *K*_*I*_. Data are plotted as mean of three independent replicates ± SEM. Error bars are not shown when smaller than the symbols. (f) MS/MS spectrum of peptide AFFIESVCDDPTVVASNIMEVK obtained by HCD fragmentation. *y* and *b* ions confirm sequence identity and localize the covalent modification to Cys154 (in red), consistent with a +469.21 Da mass shift. Fragment annotation and scores were generated with the software Skyline. (g) Residual activity of PFKFB1–4 (2.5 μM) incubated with **6** at 3 μM concentration for 30 min at 20 °C in a buffer solution containing 10 mM sodium phosphate, 50 mM NaCl, 0.2% Tween 20, pH 7.5. Each data point is the mean of three replicates ± SEM. Statistical analysis was performed using a paired two-tailed *t* test (*p < 0.05, ***p < 0.001).

Once we established that **5**–**7** are direct inhibitors of PFKFB3, we investigated their mechanism of inhibition through a trifluoroacetic acid (TFA)-induced coprecipitation assay, after testing that TFA produced full precipitation of the protein without affecting the solubility of **5**–**7**. The residual concentrations of **5**–**7** upon incubation with the enzymes were then measured by UV–visible absorbance spectrophotometry (Figure S5a). In the mixtures containing PFKFB3, coprecipitation of protein and compounds was observed (Figure 2d). On the contrary, the concentration of the compounds remained unaltered when incubated with the C154S variant, hinting at Cys154 as the reactive residue. The irreversibility of binding was confirmed by dialysis experiments: after incubating PFKFB3 with compounds **5**–**7**, dialysis failed to restore enzyme activity, consistent with covalent inhibition (Figure S5b).

After establishing the covalent nature of the inhibition, the Kitz–Wilson analysis using eq 2 ^44^ was performed by monitoring inhibition kinetics at different concentrations of **6** using the coupled enzyme assay (Figure 2e). The resulting *K*_*I*_ was 11.3 μM, the *k*_inact_ was 0.018 s^–1^ and the *k*_inact_/*K*_*I*_ ratio was 1.62·10^3^ M^–1^·s^–1^ (Table 1), within the lower range of clinically relevant irreversible inhibitors. The rate acceleration of **6** with PFKFB3 relative to βME, expressed as the ratio between the enzymatic inactivation efficiency (*k*_inact_/*K_I_*) and the intrinsic chemical reactivity toward βME (*k*_chem_, see Figure S2), was approximately 8·10^4^. This enhancement likely arises from favorable noncovalent interactions and optimal positioning of the electrophile within the enzyme binding site. The reaction was also followed using the coprecipitation assay at near-stoichiometric concentrations of PFKFB3 and **6**, confirming the association between enzyme inhibition and covalent binding (Figure S5c).

To independently estimate the noncovalent binding affinity of **6**, we determined its *K*_*i*_ for the C154S variant by enzyme assays, obtaining a value of 90 nM, comparable to that of **4** (130 nM, Table 1). This result indicated that the structural modifications introduced in **4** to generate **6** did not affect its intrinsic affinity for the enzyme. The *K*_*i*_ for the C154S variant (90 nM) was much lower than the Kitz–Wilson-derived *K*_*I*_ observed for PFKFB3 (11.3 μM), suggesting that **6** may bind the enzyme more tightly than suggested by the kinetic analysis, based on the otherwise very similar behavior between the two variants (Table 1). Such instances can be associated with a non-negligible *k*_inact_ relative to the dissociation rate constant *k*_off_ of the enzyme–inhibitor complex, potentially leading to deviation from rapid equilibrium conditions (eq 3 and 4 in the Experimental Section).

To confirm covalent inhibition and identify the reactive cysteine, we performed LC-MS/MS experiments. PFKFB3 was incubated with **6** at 150 μM concentration for 30 min prior to tryptic digestion and analysis by LC-MS/MS. Through MaxQuant analysis, two **6**-modified peptides were identified (Table S3). Peptide AFFIESVCDDPTVVASNIMEVK, encompassing Cys154, exhibited high-confidence modification, with a localization probability of 0.79, a PEP of 2.8·10^–75^, a localization score of 166.3, and a delta score of 192.98 (Figure 2f). The precursor ion intensity was 4.74·10^9^. A secondary peptide, PSCLPPEVPTQLPGQNMK, containing Cys493, showed a lower localization probability (0.55395), a localization score of 110.23 and an intensity of 2.41·10^9^. Overall, these results indicate that, among the 14 cysteine residues present in PFKFB3, Cys154 was the most probable site of covalent modification by **6**. In the untreated protein, the peptide AFFIESVCDDPTVVASNIMEVK was confidently identified (score 283.66, PEP 1.05 × 10^–83^) with high intensity and strong spectral support (21 MS/MS events). Cys154 was observed in its carbamidomethylated form, consistent with alkylation during sample preparation (Figure S6).

To evaluate selectivity, we assessed the reactivity of the PFKFB1–4 isoforms, which share 85–86% sequence similarity and all contain a conserved cysteine residue corresponding to Cys154 in PFKFB3, as determined by BLASTp analysis. PFKFB1–4 (2.5 μM each) were incubated with compound **6** (3 μM) for 30 min at 20 °C. Under these conditions, PFKFB3 was almost completely inhibited, whereas PFKFB2 showed a moderate reduction in activity (∼40%). In contrast, no significant inhibition was observed for PFKFB1 or PFKFB4 (Figure 2g). These results support a clear preference of compound **6** for the PFKFB3 isoform. When more permissive thresholds were applied, PSI-BLAST analysis (5 iterations; inclusion threshold *E*-value ≤ 0.005) retrieved additional, more distantly related proteins, such as phosphoglycerate mutases (PGAM1, PGAM2, PGAM4), bisphosphoglycerate mutase, and TIGAR, displaying low sequence similarity (∼15–30% identity) and partial coverage (∼30–60%). None of the retrieved sequences showed alignment with PFKFB3 in the region encompassing Cys154.

Finally, the selectivity of compound **6** (10 μM) for PFKFB3 was assessed in a DiscoverX scanMAX kinase assay against 97 kinases, ranging from tyrosine kinases (e.g., ABL1, EGFR, SRC), serine/threonine kinases (e.g., AKT, CDKs, MAPKs), and lipid kinases. Overall, **6** exhibited very weak binding across the panel, with %Ctrl values generally remaining high (>35%), indicating low affinity interactions for most targets (Figure S7 and Table S4). Only marginal reductions in %Ctrl were observed for a few kinases (e.g., RIOK2, nonphosphorylated ABL1, LKB1), but none reached thresholds typically associated with meaningful inhibition. Consistently, selectivity scores S(35), S(10), and S(1) were all equal to 0, confirming the absence of significant hits.

It should be noted that neither isoform kinetic selectivity nor the lack of reactivity within the investigated panel of protein and lipid kinases can exclude potential off-target effects, which may arise from reactivity toward cysteine residues in homologous or unrelated proteins, where the reaction may be driven by *k*_inact_ rather than *K*_*I*_, as well as from noncovalent interactions that are independent of covalent bond formation.

### Profiling of Covalent Inhibitors in PDAC

To confirm the potential relevance of PFKFB3 in PDAC, we performed a series of bioinformatic analyses integrating bulk and single-cell gene expression data sets. Using the GEPIA2 platform, we observed that PFKFB3 mRNA levels are significantly higher in PDAC tissues compared with normal pancreas (Figure S8a). This was further supported by single-cell RNA sequencing (scRNA-seq) data from the Pancreatic Tissue Single Cell Atlas, which revealed that PFKFB3 expression is enriched in malignant epithelial cells rather than in normal ductal populations (Figure S8b and S8c). Moreover, Kaplan–Meier survival analyses indicated that elevated PFKFB3 expression correlates with reduced disease-free and, in tumors with grade ≥ G2, with overall survival (Figure S8d and S8e). Collectively, these results confirm PFKFB3 as a clinically relevant metabolic driver in PDAC. To identify the most representative cellular models for testing our PFKFB3 inhibitors, we next examined the expression of PFK and PFKFB3 by Western blotting in a panel of pancreatic cell lines. Normal human dermal fibroblasts (NHDF) showed no detectable expression of any of the enzymes. In contrast, both immortalized pancreatic ductal epithelial cells (HPDE) and six PDAC-derived cell lines displayed robust PFKFB3 and PFK expression (Figure 3a). These results are consistent with the bioinformatic findings and confirm that PFKFB3 upregulation is a hallmark of PDAC, supporting the use of these cellular models for subsequent inhibitor evaluation. At first, the compounds (**4**–**7** and **5r**) were tested for their in-cell selectivity using these two nontumorigenic human cell lines. No cytotoxic effects were observed in NHDF cells at concentrations up to 50 μM, indicating a complete lack of activity of the compounds in PFKFB3-negative fibroblasts. In contrast, only minimal cytotoxicity was observed in HPDE cells, with cell viability remaining above 50% even at the highest tested concentrations (Figure 3b). These results suggest a favorable selectivity profile, with negligible activity in nontumorigenic cells regardless of PFKFB3 expression. Afterward, we assessed the cytotoxic activity of the compounds across a panel of human PDAC cell lines, including PANC-1, MIA PaCa-2, AsPC-1, Hs 766T, PaCa-3, and SUIT-2. All compounds exhibited a clear dose-dependent cytotoxic response, with IC_50_ values ranging from 15 to 30 μM depending on the cell line. Among all tested molecules, the covalent inhibitor **6** and the reversible inhibitor **5r** consistently demonstrated the most potent cytotoxic activity, identifying them as the most promising compounds for further studies (Figure 3c).

**Figure 3:**
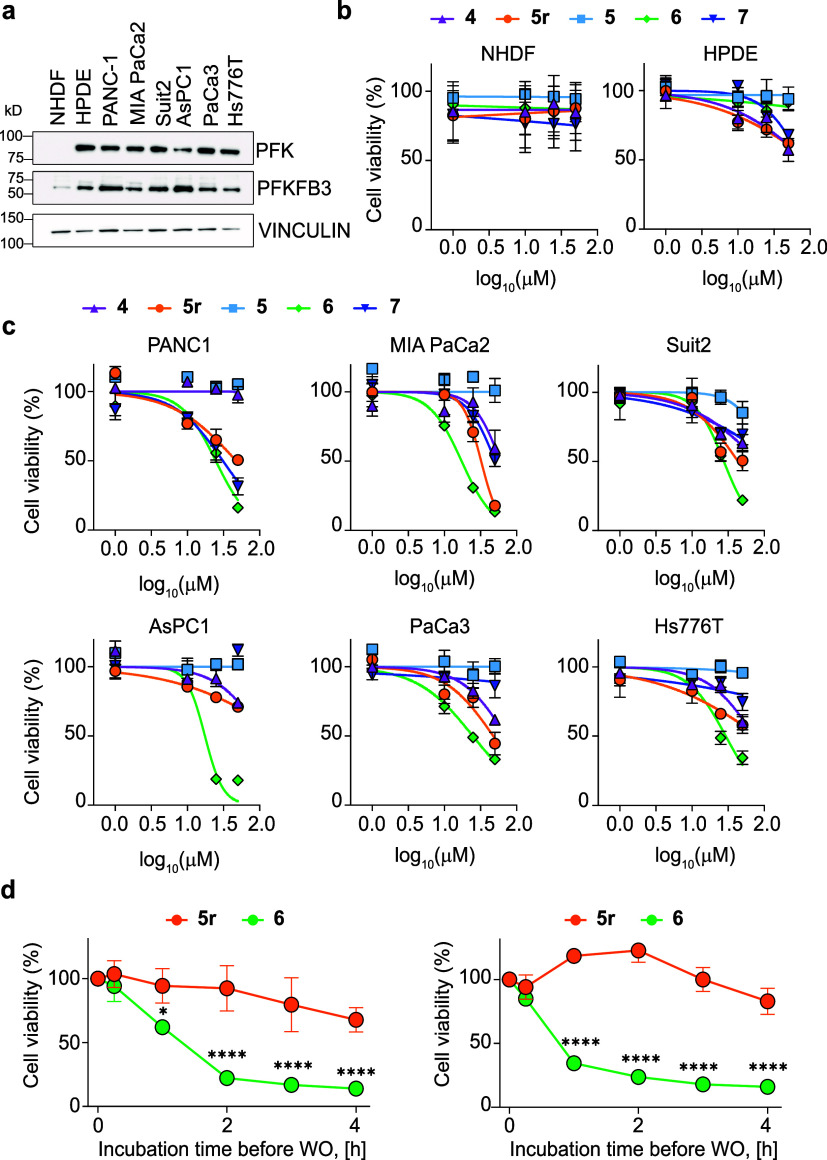
*In vitro* efficacy of covalent PFKFB3 inhibition in PDAC cell models. (a) Immunoblot analysis of PFK and PFKFB3 expression in NHDF, HPDE1, PANC-1, MIA PaCa-2, Suit2, AsPC1, PaCa3, and Hs776t cell lines. Vinculin was used as a loading control. (b, c) Dose–response curves showing cell viability in (b) NHDF and HPDE1 cells and (c) human PDAC cell lines (PANC-1, MIA PaCa-2, Suit2, AsPC1, PaCa3, Hs776t) following 48 h treatment with increasing concentrations of compounds **4**–**7** and **5r** (1, 10, 25, 50 μM). Cell viability was measured by MTT assay and expressed as a percentage relative to the DMSO control. The *Y*-axis indicates cell viability (%), and the *X*-axis shows compound concentration (log_10_[μM]). Data plotted are mean of three independent replicates ± SD. Error bars are not shown when smaller than the symbols. (d) PANC-1 (left) and MIA PaCa-2 (right) cells were treated with **5r** or **6** (25 μM) for 15 min, 1, 2, 3, or 4 h. After treatment, free inhibitor was washed out and replaced with fresh medium. The residual effect of the inhibitors was quantified by Crystal Violet staining after 72 h. Data plotted are mean of three independent replicates ± SD. Error bars are not shown when smaller than the symbols. Statistical analysis was performed using two-way ANOVA with Sidak’s multiple comparisons test (*p < 0.05, ***p < 0.001, ****p < 0.0001).

To investigate the discrepancy between cellular IC_50_ and enzymatic *K_i_* values, and to assess whether limited cellular permeability could account for the reduced cellular potency, we performed a Caco-2 bidirectional permeability assay for compound **6**, using propranolol and erythromycin as reference controls. Compound **6** demonstrated moderate permeability and an efflux ratio greater than 3, suggesting the possibility of transporter-mediated efflux (potentially involving P-gp). Overall, the recovery of quality control (QC) samples fell within the acceptable range. However, the recovery of compound **6** was only 25.98% (A → B) and 57.44% (B → A; Table S5 in the Supporting Information), which falls below the typically acceptable recovery range of 80–120%. This might be attributed to degradation in the assay buffer or nonspecific binding to the membrane.

### Validation of Extended Duration of Action in Cellular Washout Studies

To gain further insight into the long-term inhibition of PFKFB3, we focused on MIA PaCa-2 and PANC-1 cell lines, which were selected due to their complementary PDAC-relevant features. These lines harbor distinct driver mutations frequently observed in PDAC (*KRAS* and *TP53*) and exhibit divergent metabolic profiles, with MIA PaCa-2 being strongly glycolytic and PANC-1 primarily relying on oxidative phosphorylation.^45^ This combination allows assessment of prolonged compound activity across genetically and metabolically diverse PDAC models. Cells were incubated with 25 μM of each compound for varying durations (15 min, 1, 2, 3, or 4 h), after which the compounds were extensively washed out to remove any residual free compound. Cell viability was then assessed at 72 h (h) post-treatment. The results revealed that a 1 h incubation with compound **6** was sufficient to significantly impair long-term cell viability in both cell lines, indicating that transient exposure to this compound effectively exerts prolonged cytotoxic effects. In contrast, noncovalent compound **5r** completely lost its cytotoxic activity under the same conditions, demonstrating the potential of our covalent molecule for sustained target inhibition (Figure 3d). Finally, to extend our analysis beyond pancreatic models, we first assessed PFKFB3 levels by Western blot in a panel of human cancer cell lines from lung (A549), kidney (ACHN, 293T), and CNS tumors (glioblastoma U251, neuroblastoma SH-SY5Y), confirming robust expression across the whole panel. Consistently, compound **6** showed strong antiproliferative effects in these lines. In contrast, nontransformed fibroblasts (BJ, WI-38), which lacked detectable PFKFB3, were less sensitive to treatment, supporting an on-target mechanism of action (Figure S9a and S9b).

### Evaluation of *In Vivo* Efficacy in a Zebrafish Xenograft Model

To further evaluate the antitumor efficacy of compound **6** *in vivo*, we used a zebrafish xenograft model. Prior to these experiments, a dose titration study was performed in 3 days postfertilization (dpf) zebrafish larvae to determine the toxicity profile of compound **6**, testing a range of concentrations (7, 10, 15, 25, and 50 μM) and assessing viability and locomotor alterations after 48 h. Based on these results (summarized in Table S6), 7 μM was selected for subsequent experiments, as it was fully tolerated with all larvae viable and responsive. MIA PaCa-2 and PANC-1 cells were fluorescently labeled with the Vybrant lipophilic membrane dye to enable visualization after injection into 2 dpf embryos. At 1-day postinjection (dpi), fluorescence imaging was performed to assess initial tumor cell engraftment, and larvae were treated with 7 μM of compound **6** in fish water. The fluorescence intensity of the injected cells was monitored at 24 (T1) and 48 h (T2) post-treatment. Control larvae showed a decrease in fluorescence over time, likely reflecting physiological clearance, dye dilution during cell proliferation, partial redistribution to host cells, or changes in membrane integrity. In contrast, larvae treated with compound **6** exhibited a pronounced and more statistically significant reduction in fluorescence intensity, especially at T2 (Figure 4a and b). Although some decrease in fluorescence was also observed in control larvae, the reduction in compound **6**-treated larvae was statistically significant, indicating a specific antitumor effect beyond baseline signal decay. The strong efficacy observed at 7 μM reflects the high sensitivity of these pancreatic cancer cells to compound **6** in the zebrafish xenograft system, where direct exposure in a small aquatic volume allows efficient drug uptake and rapid pharmacological effects.

**Figure 4:**
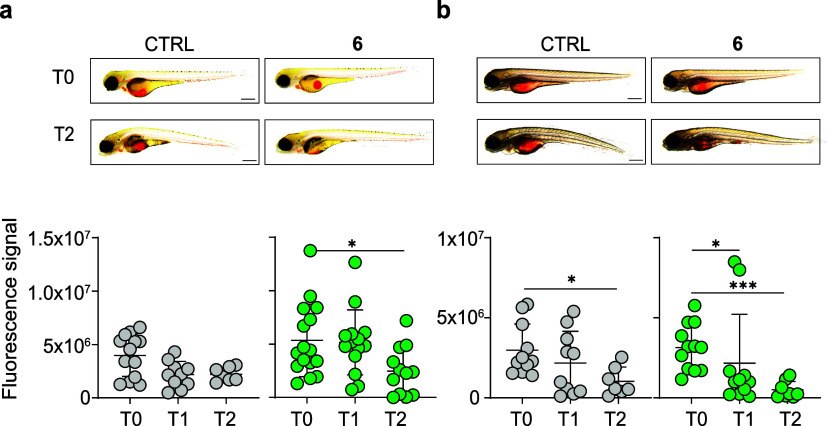
*In vivo* efficacy of covalent PFKFB3 inhibition in zebrafish xenografts. (a, b) Fluorescence quantification in MIA PaCa-2 (a) and PANC-1 (b) zebrafish xenografts treated with vehicle (CTRL) or compound **6** (7 μM). Fluorescent signals were measured before the treatment (T0), 24 h (T1) and 48 h (T2) post-treatment. Sample sizes were as follows: PANC-1 CTRL (T0 *n* = 11, T1 *n* = 10, T2 *n* = 8), PANC-1 compound 6 (T0 *n* = 12, T1 *n* = 11, T2 *n* = 9); MIA PaCa-2 CTRL (T0 *n* = 15, T1 *n* = 11, T2 *n* = 8), MIA PaCa-2 compound 6 (T0 *n* = 16, T1 *n* = 14, T2 *n* = 12). Scale bars: 300 μm for T0 and 400 μm for T2. Statistical analysis was performed using Dunn’s multiple comparisons test (*p < 0.05, ***p < 0.001).

### Metabolic Stability

To evaluate the metabolic stability of **6**, the compound was incubated at both 1 and 10 μM *in vitro* with microsomes from different animal species. Liver microsomes from humans (HLM, Xenotech, cat. No. H0610), Sprague–Dawley rats (RLM, Xenotech, cat. No. R1000), CD-1 mice (MLM, Xenotech, cat. No. M1000) and Beagle dogs (DLM, Gibco, cat. No. DGMCPL) were exploited to evaluate interspecies variability in metabolic clearance and pinpoint the best animal model for future studies. After incubation with rat and human liver microsomes, compound **6** showed a moderate stability with half-lives were >1 h for both species (68 min in HLM vs >120 min in RLM at 10 mM compound concentration, Table 1 and Figure 5). Species differences between rat and human liver microsomes were relatively minor, as indicated by resulting medium-to-low intrinsic clearance (CL_int_) rates of 21.10 mL/min/mg protein for HLM and 9.08 mL/min/mg protein for RLM (Tables S7 and S8 in Supporting Information). On the contrary, the clearance with mouse and dog liver microsomes was higher, resulting in CL_int_ rates of 118.88 mL/min/mg protein for mouse liver microsome (MLM) and 113.61 mL/min/mg protein for dog liver microsomes (DLM), Tables S9 and S10 in Supporting Information). Compound **6** was moderately stable in human liver microsomes (HLM) and highly stable in rat liver microsomes (RLM), whereas it was rapidly metabolized in mouse and dog liver microsomes (half-lives <15 min for both species, Table 2 and Figure 5). As **6** was much more stable in rat liver microsomes (RLM) than in mouse liver microsomes (MLM), rats could be exploited in the future for the preclinical assessment of toxicity and efficacy. The %QH (percentage of hepatic blood flow) was determined for estimation of the ability of drug clearance from the liver. It reflects the ratio of intrinsic clearance to the liver blood flow rate and is usually applied for the classification of substances by their intrinsic clearance profile. Hence, the %QH value allows us to evaluate In Vitro to In Vivo Extrapolation (IVIVE) and metabolic stability of the drug. Compound **6** showed a low clearance in human and rat with <30% QH (Tables S7 and S8 in Supporting Information) whereas in mouse and dog it showed high clearance with >70% QH value. In addition, compound **6** shows degradation in the absence of cofactor NADPH (Tables S7–S10 in Supporting Information). These findings suggest nonenzymatic or NADPH-independent enzymatic degradation, possibly due to intrinsic chemical instability, non-CYP450 enzymatic activity, or other degradation processes.

**Figure 5:**
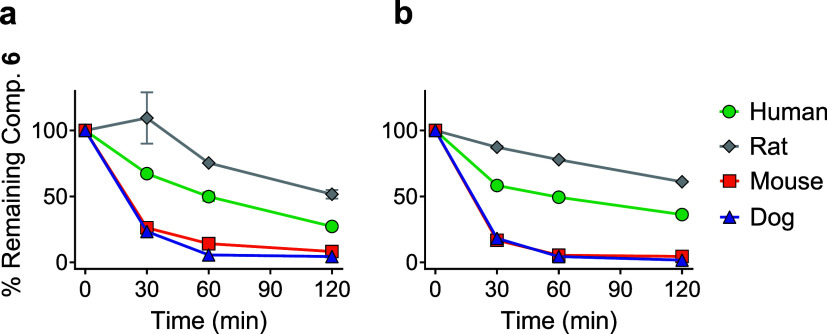
(a, b) Metabolic stability study of compound **6** at concentrations of 1 μM (a) and 10 μM (b) in liver microsomes of mouse, rat, dog, and human. The data is shown as percentage of parent compound remaining over time. The data is in terms of means (*n* = 2), Error bars are not shown when smaller than the symbols. See the corresponding numerical data in Tables S7–S10 of the Supporting Information. The species are indicated as mouse (red), rat (gray), dog (blue), and human (green).

**Table 2:** Stability of Compound **6** in Human, Rat, Mouse, and Dog Microsomes^a^

	Half-life (min)				CL_int_ protein (μL/min/mg protein)			
Compound	HLM	RLM	MLM	DLM	HLM	RLM	MLM	DLM
6 (1 μM)	62.68	109.43	15.53	14.30	22.24	12.67	89.28	96.95
6 (10 μM)	68.18	>120	11.66	12.24	21.10	9.08	118.88	113.61

a HLM: Human Liver Microsomes; RLM: Rat Liver Microsomes; MLM: Mouse Liver Microsomes; DLM: Dog Liver Microsomes.

### Investigation of the Direct Effect of Compound **6** on Glycolysis

To assess the cellular efficacy of our lead compound in the MIA PaCa-2 cell model, PFK activity was measured in cell lysates following a 2 h incubation with either the covalent compound **6** or the reversible molecule **5r** at a final concentration of 25 μM. After incubation, cells were extensively washed to remove any noncovalently bound material prior to lysis. A statistically significant reduction in PFK activity was observed in cells treated with **6**, whereas no significant change was detected in cells exposed to compound **5r** compared with untreated controls (Figure 6a).

**Figure 6:**
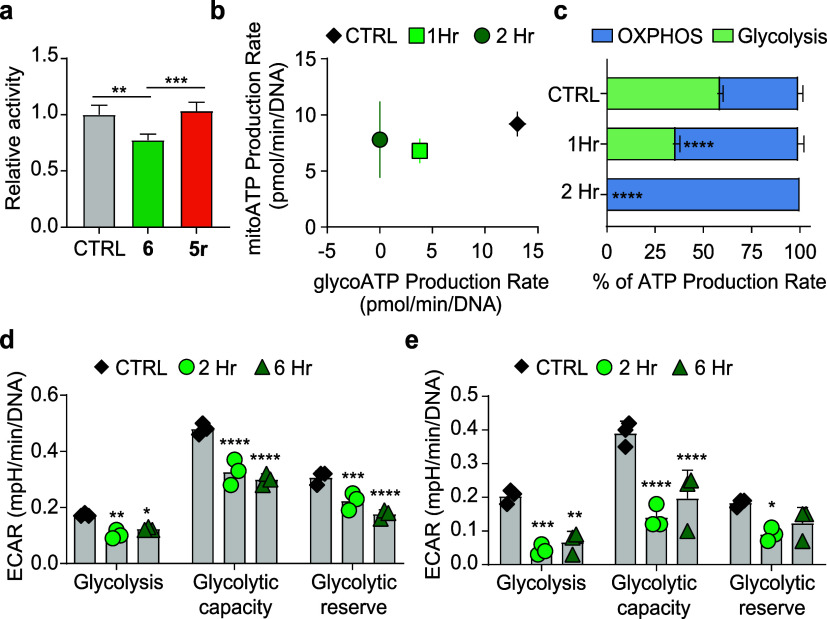
Covalent PFKFB3 inhibition disrupts glycolytic flux in PDAC cells. (a) PFK activity measured in MIA PaCa-2 cell lysates after 2 h treatment with compound **6** (25 μM). Cells were washed prior to lysis to remove noncovalently bound material. Data plotted are mean of five independent replicates ± SD. Statistical analysis was performed using a paired two-tailed *t* test (** *p* < 0.01, ***p < 0.001). (b, c) ATP rate assay performed 1 and 2 h after treatment with 25 μM of compound **6**. (b) shows the cellular energy map, while (c) shows the ATP production rate, divided into contributions from glycolysis and oxidative phosphorylation (OXPHOS). (d, e) Glycolysis stress tests in PANC-1 (d) and MIA PaCa-2 (e) cells following 2 and 6 h treatment with compound **6**, indicating significant decreases in glycolytic rate, glycolytic capacity, and glycolytic reserve. Data plotted are mean of three independent replicates ± SD. Statistical analysis was conducted using Tukey’s multiple comparisons test (*p < 0.05, ** *p* < 0.01, *** *p* < 0.001, **** *p* < 0.0001).

Finally, the inhibition of PFKFB3 (17 μM) by **6** (25 μM) was evaluated in the presence of a 10-fold excess (w/w) of total protein derived from a MIA PaCa-2 cell lysate (Figure S11 in Supporting Information). The percentage of inhibition remained unchanged compared to the enzyme assayed in buffer alone, indicating that **6** does not exhibit promiscuous reactivity toward intracellular proteins under the assay conditions.

These findings are consistent with the covalent mode of action of compound **6**, leading to sustained enzyme inhibition. In contrast, the lack of measurable effect following compound **5r** treatment confirms a reversible interaction with PFKFB. Collectively, these results highlight the importance of covalent binding in achieving durable target engagement and prolonged modulation of PFK activity in cells. To further test the cellular efficacy of compound **6**, we conducted ATP rate assays at two time points, 1 and 2 h post-treatment. The results revealed a rapid, time-dependent decrease in total ATP production, with glycolytic ATP production markedly reduced already at 1 h and completely abolished by 2 h in compound **6**-treated cells (Figure 6b and c). These data demonstrate that compound **6** rapidly and effectively shuts down glycolytic ATP rate in MIA PaCa-2 cells, confirming its potent intracellular activity. Importantly, the ATP rate assay provided a dynamic readout of the compound impact on cellular bioenergetics, enabling precise temporal resolution of its inhibitory effects on glycolytic ATP generation.

Furthermore, glycolysis stress tests performed on MIA PaCa-2 and PANC-1 cells demonstrated a significant decrease in key glycolytic parameters, including glycolytic rate, capacity, and reserve, after only 1 h of treatment (Figure 6d and e). These findings suggest that **6** effectively impairs the glycolytic flux and the ability of cancer cells to respond to metabolic stress, further supporting its mechanism of action as a covalent PFKFB3 inhibitor.

### Evaluation of Synergism of Compound **6** and Chemotherapy

Resistance to chemotherapy is a major hurdle in PDAC treatment, limiting the effectiveness of standard regimens such as gemcitabine and FOLFIRINOX (a combination of folinic acid, fluorouracil, irinotecan, and oxaliplatin).^46,47^ This challenge highlights the need for new therapeutic approaches to improve patient outcomes. In this context, we sought to investigate the potential synergistic effects between our covalent PFKFB3 inhibitor, compound **6**, and chemotherapeutic agents commonly used in PDAC treatment. The two most aggressive and chemoresistant PDAC cell lines, MIA PaCa-2 and PANC-1, were selected for this study. Compound **6** was tested in combination with gemcitabine at a fixed ratio of 1:10, as well as with a FOLFIRINOX-like cocktail (excluding folinic acid, FOI),^48^ to evaluate whether the metabolic inhibition mediated by compound **6** could enhance chemotherapy efficacy. Cell viability assays were performed after treatment with increasing concentrations of **6**, chemotherapeutic agents alone, or their combinations. In both cell lines, single-agent treatments with either compound **6** or chemotherapy resulted in a dose-dependent reduction in cell viability (Figure 7a–d). Notably, the combined treatments produced a more pronounced cytotoxic effect than either drug alone, particularly at intermediate and higher concentrations, suggesting a potentiating interaction between compound **6** and the chemotherapeutics. To quantitatively assess drug interactions, data were analyzed using CompuSyn software following the Chou–Talalay method. In MIA PaCa-2 cells, the combination of compound **6** with FOI or gemcitabine showed additive effects at low fractional effects (Fa < 0.4) and clear synergism at higher Fa values (Fa > 0.5), as confirmed by isobologram analysis (Figure 7e,f). In PANC-1 cells, compound **6** combined with FOI displayed strong synergy at low to intermediate effect levels (Fa < 0.6; CI 0.3–0.5), becoming additive at higher Fa (Figure 7g). Conversely, the compound **6** plus gemcitabine combination maintained robust synergy over a wider range (Fa = 0.1–0.8; CI 0.3–0.6), turning additive only at very high Fa (>0.9, Figure 7h). Overall, the dose–response and combination index analyses demonstrate that compound **6** enhances the cytotoxic activity of both FOI and gemcitabine in PDAC cells, suggesting that metabolic inhibition via PFKFB3 targeting may sensitize tumor cells to chemotherapy-induced stress, thereby offering a promising combinatorial therapeutic approach for chemoresistant PDAC.

**Figure 7:**
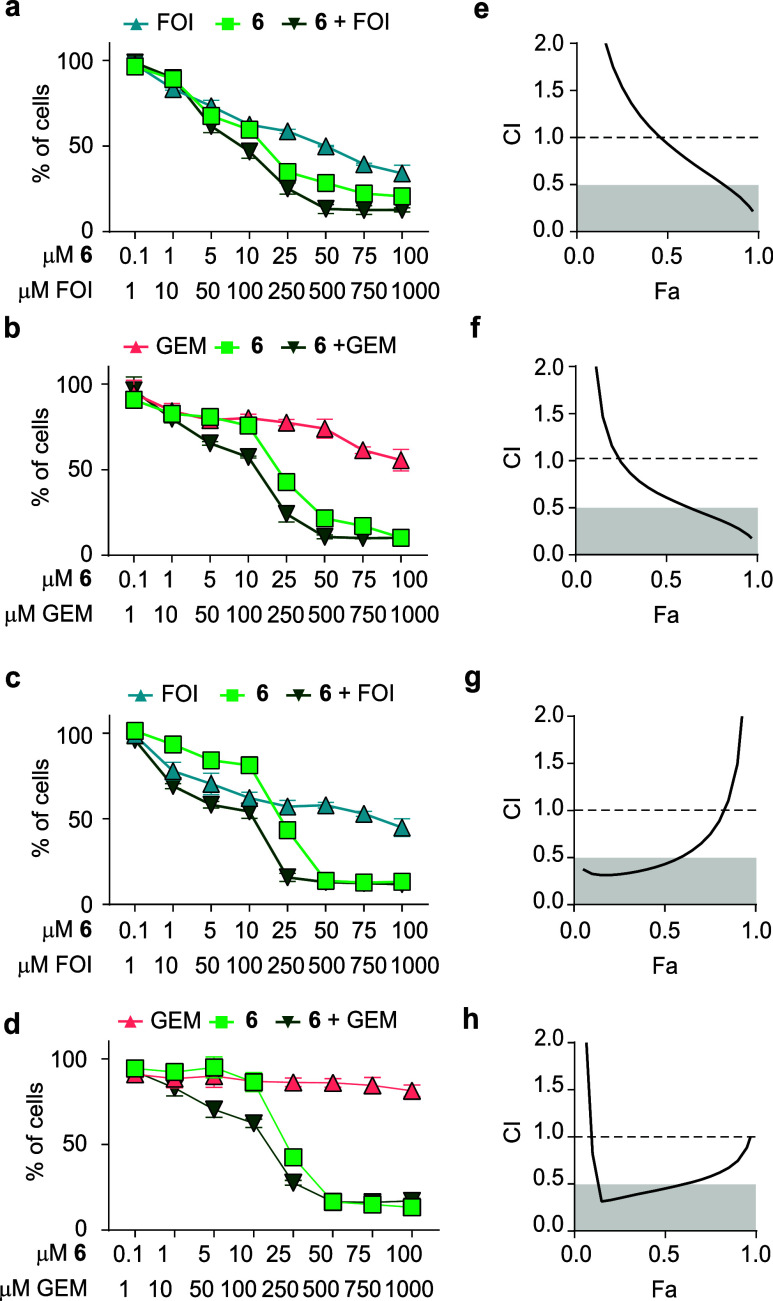
Synergistic anticancer effects of compound **6** combined with chemotherapy in chemoresistant PDAC cell lines. (a–d) Dose–response curves showing cell viability of MIA PaCa-2 (a, b) and PANC-1 (c, d) cells treated with increasing concentrations of compound **6**, chemotherapeutic agents alone, or their combinations in a 1:10 molar ratio. Data plotted are mean of three independent replicates ± SD. Error bars are not shown when smaller than the symbols. (e–h) Combination index (CI) analysis of compound **6** in combination with FOI or gemcitabine in MIA PaCa-2 (e, f) and PANC-1 (g, h) cells, calculated using the Chou–Talalay method. CI values are plotted as a function of the fractional effect (Fa) for each treatment condition.

## Conclusions

By mapping the structure of PFKFB3, we identified Cys154 as a nucleophilic hotspot situated at the edge of the ATP-binding pocket of the kinase. Cys154 does not directly participate in catalysis, and its substitution with a Ser residue only minimally affects the binding of substrates and reversible ligands. This suggests that covalent engagement at this site can be achieved without compromising essential enzyme conformational dynamics. Leveraging on the structure of a known high-affinity reversible inhibitor (compound **4**), we designed the first-in-class covalent inhibitor of PFKFB3 (compound **6**), opening the door to covalent ligand applications for this kinase. The kinetic behavior of **6** is characteristic of a two-step covalent inhibitor. Kitz–Wilson analysis revealed *k*_inact_/*K*_*I*_ in the lower but pharmacologically relevant range for clinically used covalent inhibitors. Notably, independent affinity measurements using the C154S variant indicated that the reversible component of binding remains in the nanomolar range, similar to the parent noncovalent scaffold. This validates our design strategy: introducing a warhead into the indole ring does not compromise recognition of the ATP pocket but creates a productive alignment for irreversible modification. The >10^4^-fold rate acceleration of **6** for PFKFB3 relative to βME underscores that the active site microenvironment, rather than electrophile strength, dictates covalent bond formation. This catalysis-by-proximity results from a network of anchoring hydrogen bonds and a prereactive orientation observed in MD simulations and docking free-energy distributions. Mass spectrometry unambiguously confirmed Cys154 as the primary site of covalent labeling, with only minor reactivity observed at more distal cysteines. This high site-selectivity is particularly significant given the large number of potential nucleophiles within PFKFB3 and the relatively reactive nature of cysteine residues in glycolytic enzymes. Taken together, these data indicate that **6** establishes a specific, structurally guided covalent bond with Cys154, rather than reacting opportunistically with surface-exposed thiols.

Compound **6** proved capable of reducing the viability of PDAC cell lines, *in vitro* and in a xenograft model. These findings highlight covalent PFKFB3 inhibition as a strategic approach to exploit metabolic vulnerabilities in PDAC. The prolonged cytotoxicity following transient exposure to compound **6** highlights the importance of irreversible target engagement expected for a covalent inhibitor, although we acknowledge that washout experiments alone are insufficient to definitively demonstrate on-target activity. The selective vulnerability of PFKFB3-positive tumor cells, alongside minimal effects in nontransformed fibroblasts, reinforces an on-target mechanism. The rapid suppression of glycolytic ATP production and depletion of glycolytic reserve further suggest that irreversible inhibition of PFKFB3 constrains the adaptive metabolic capacity of cancer cells, providing a mechanistic basis for its synergy with chemotherapeutic agents. Indeed, the observed synergy with chemotherapeutics reinforces the concept that acute metabolic disruption can potentiate traditional cytotoxic stress, providing a mechanistic rationale for integrating covalent metabolic inhibitors into multidrug regimens. Importantly, the zebrafish xenograft studies offer a physiologically relevant *in vivo* validation: direct exposure of implanted PDAC cells to compound **6** in a small aquatic volume allowed efficient drug uptake and rapid pharmacological effects, confirming that the covalent inhibitor can elicit antitumor activity in a complex multicellular environment. Overall, our work provides the first proof-of-concept that Cys154 is druggable and can be exploited for the development of covalent PFKFB3 inhibitors. Further SAR studies aimed at optimizing the cellular potency of compound **6** could lead to high-quality chemical probes required to deconvolute the role of PFKFB3 in oncology and beyond.

## Experimental Section

### General Information

Reagents were purchased at the highest commercial quality from Sigma-Aldrich or Fluorochem and used without further purification. Thin layer chromatography (TLC) plates were purchased from Sigma-Aldrich (silica gel 60 F254 aluminum sheets, with fluorescence indicator 254 nm) and were analyzed with UV detection (254 nm) and/or staining with potassium permanganate alkaline solution or ninhydrin stain. Flash chromatography was performed using silica gel, pore size 60 Å, 230–400 mesh particle size, or Interchim automatic flash chromatography instrument. ^1^H NMR and ^13^C NMR spectra were recorded with a Varian Mercury 300 (300 MHz) spectrometer. NMR spectra were obtained in deuterated solvents. The chemical shifts are reported in ppm and corrected to the signal of the deuterated solvents. Chemical shifts (δ) are expressed in ppm and coupling constants (*J*) in Hertz (Hz). High-resolution mass spectrometry (HRMS) analyses were performed using the Ultimate 3000 HPLC (Dionex), set to automatically inject into the LTQ Orbitrap XL mass spectrometer (Thermo Fisher Scientific, USA), operating in full scan mode. Analyses were carried out in positive ion mode. The chromatographic purity of final compounds was determined by high performance liquid chromatography (HPLC) analysis on a Waters 1525 Binary HPLC Pump, equipped with a Waters 2489 UV–vis detector (Waters, Milford, MA), using a Symmetry C18 Column (4.6 × 75 mm, 3.5 μm particle size). Solvents A and B (A = Millipore water with 0.1% TFA, B = ACN with 0.1% TFA) and three different methods were used. Method: gradient elution (85:15 for 0.2 min, 85:15 → 5:95 over 14 min) of the mobile phase consisting of solvents A and B at a flow rate of 1 mL/min at room temperature. The purity of all final compounds was >95%.

#### 5-(4-Nitrophenoxy)-1*H*-indole (**9**)

To a stirred solution of 1-*H*-indol-5-ol **8** (1.00 g, 7.51 mmol, 1.0 equiv) in dry DMF (13 mL) under nitrogen atmosphere, 1-fluoro-4-nitro-benzene (1.10 g, 7.51 mmol, 1.0 equiv), and Cs_2_CO_3_ (2.90 g, 9.01 mmol, 1.2 equiv) were added. The reaction mixture was stirred at room temperature overnight. After completion of the reaction, AcOEt was added, and the organic phase was washed with water (×3) and brine (×1). The organic phase was dried over anhydrous Na_2_SO_4_, filtered and evaporated under reduced pressure. Purification by column chromatography on silica gel (cyclohexane/AcOEt 8:2) afforded compound **9** as yellow solid (1.70 g, 6.81 mmol, 75% yield). Characterization data in accordance with the literature.^21^ ^1^H NMR (300 MHz, DMSO-*d*_6_) δ 11.26 (br, 1H), 8.24–8.16 (m, 2H), 7.48 (d, *J* = 8.7 Hz, 1H), 7.43 (dd, *J* = 2.4, 2.4 Hz, 1H), 7.34 (d, *J* = 2.3 Hz, 1H), 7.06–6.99 (m, 2H), 6.88 (dd, *J* = 8.7, 2.3 Hz, 1H), 6.46–6.42 (m, 1H).

#### 3-Bromo-5-(4-nitrophenoxy)-1*H*-indole (**10**)

To a stirred solution of **9** (1.70 g, 6.81 mmol, 1.0 equiv) in dry DMF (20 mL) under nitrogen atmosphere, *N*-bromosuccinimide (1.21 g, 6.81 mmol, 1.0 equiv) was added. The reaction mixture was stirred at room temperature for 2 h. After completion of the reaction, AcOEt was added, and the reaction mixture was washed with water (×3) and brine (×1). The organic phase was dried over anhydrous Na_2_SO_4_, filtered and evaporated under reduced pressure. Purification by column chromatography on silica gel (cyclohexane/AcOEt 9:1) afforded compound **10** as a brown solid (2.26 g, 6.81 mmol, quantitative yield). ^1^H NMR (300 MHz, DMSO-*d*_6_) δ 11.66 (br, 1H), 8.24–8.17 (m, 2H), 7.65 (d, *J* = 2.4 Hz, 1H), 7.53 (d, *J* = 8.7 Hz, 1H), 7.15 (d, *J* = 2.4 Hz, 1H), 7.09–7.03 (m, 2H), 7.00 (dd, *J* = 8.7, 2.4 Hz, 1H). ^13^C NMR (75 MHz, DMSO-d6) δ 164.74, 148.36, 142.21, 133.55, 127.36, 127.25, 126.59, 117.10, 116.53, 114.47, 109.71, 89.12.

#### 3-(1-Methyl-1*H*-pyrazol-4-yl)-5-(4-nitrophenoxy)-1*H*-indole (**11**)

To a stirred solution of **10** (2.00 g, 6.00 mmol, 1.0 equiv) in dry dioxane (30 mL) under nitrogen atmosphere, 1-methyl-4-(4,4,5,5-tetramethyl-1,3,2- dioxaborolan-2-yl)­pyrazole (2.50 g, 12.00 mmol, 2.0 equiv), palladium tetrakistriphenylphosphine (556 mg, 0.48 mmol, 0.08 equiv) and a 1 M K_3_PO_4_ degassed solution (12 mL, 12.00 mmol, 2.0 equiv) were added. The reaction mixture was stirred at 80 °C overnight. After completion of the reaction, the aqueous phase was extracted with AcOEt (×3). The organic phase was dried over anhydrous Na_2_SO_4_, filtered and evaporated under reduced pressure. Purification by column chromatography on silica gel (cyclohexane/AcOEt from 7:3 to 4:6) afforded compound **11** as a yellow solid (863 mg, 2.58 mmol, 43% yield). ^1^H NMR (300 MHz, DMSO-*d*_6_) δ 11.32 (br, 1H), 8.24–8.17 (m, 2H), 8.08 (s, 1H), 7.74 (s, 1H), 7.66 (d, *J* = 2.0 Hz, 1H), 7.58 (d, *J* = 2.3 Hz, 1H), 7.50 (d, *J* = 8.7 Hz, 1H), 7.08–7.01 (m, 2H), 6.94 (dd, *J* = 8.7, 2.3 Hz, 1H), 3.83 (s, 3H). ^13^C NMR (75 MHz, DMSO-*d*_6_) δ 165.24, 147.47, 141.89, 136.44, 134.65, 127.17, 126.54, 126.18, 123.92, 116.69, 115.82, 115.38, 113.65, 111.32, 108.50, 38.83.

#### 4-((3-(1-Methyl-1*H*-pyrazol-4-yl)-1*H*-indol-5-yl)­oxy)­aniline (**12**)

To a stirred solution of **11** (863 mg, 2.58 mmol, 1.0 equiv) in MeOH (18 mL) under nitrogen atmosphere, 10% palladium on charcoal (200 mg, 0.7 equiv) was added. The reaction mixture was stirred at room temperature for 2 h under hydrogen atmosphere (1 atm). After completion of the reaction, the suspension was filtered on Celite. The filtrate was evaporated under reduced pressure to afford compound **12** as a brown solid (785 mg, 2.58 mmol, quantitative yield). ^1^H NMR (300 MHz, DMSO-*d*_6_) δ 11.15 (br, 1H), 7.99 (s, 1H), 7.67 (s, 1H), 7.55 (d, *J* = 2.5 Hz, 1H), 7.37 (d, *J* = 8.8 Hz, 1H), 7.33 (d, *J* = 2.3 Hz, 1H), 6.80 (dd, *J* = 8.8, 2.3 Hz, 1H), 3.84 (s, 3H), 2.49 (br, 2H). ^13^C NMR (75 MHz, DMSO-*d*_6_) δ 151.78, 149.22, 144.54, 136.35, 133.21, 127.01, 126.07, 123.29, 119.40, 116.22, 115.25, 114.11, 112.82, 108.24, 107.85, 38.86.

#### *tert*-Butyl-2-((4-((3-(1-methyl-1*H*-pyrazol-4-yl)-1*H*-indol-5-yl)­oxy)­phenyl)­carbamoyl)­pyrrolidine-1-carboxylate (**13**)

To a stirred suspension of **12** (785 mg, 2.58 mmol, 1.0 equiv) in dry CH_2_Cl_2_ (25 mL) under nitrogen atmosphere, (*tert*-butoxycarbonyl)-l-proline (722 mg, 3.35 mmol, 1.3 equiv), *N*,*N*,*N*,*N*-tetramethyl-*O*-(1*H*-benzotriazol-1-yl)­uronium hexafluorophosphate (HBTU, 1.50 g, 3.87 mmol, 1.5 equiv), and DIPEA (667 mg, 0.9 mL, 5.16 mmol, 2.0 equiv) were added. The reaction mixture was stirred at room temperature for 4 h. Then, the organic solvent was evaporated under reduced pressure, and the crude was directly purified by column chromatography on silica gel (cyclohexane/AcOEt from 3:7 to 2:8) to afford compound **13** as a yellow solid (1.29 g, 2.58 mmol, quantitative yield). Characterization data in accordance with the literature.^21^ ^1^H NMR (300 MHz, DMSO-*d*_6_) δ 9.35 (s, 1H), 8.31 (s, 1H), 7.69 (s, 1H), 7.58 (s, 1H), 7.44–7.39 (m, 3H), 7.36 (d, *J* = 8.7 Hz, 1H), 7.31 (d, *J* = 2.4 Hz, 1H), 6.97 (d, *J* = 8.7, 2.4 Hz, 1H), 6.95–6.89 (m, 2H), 4.51–4.36 (m, 1H), 3.94 (s, 3H), 3.57–3.30 (m, 2H), 2.01–1.87 (m, 2H), 1.67–1.58 (m, 2H), 1.48 (s, 9H).

#### *tert*-Butyl (*S*)-2-((4-((1-Acryloyl-3-(1-methyl-1*H*-pyrazol-4-yl)-1*H*-indol-5-yl)­oxy)­phenyl)­carbamoyl)­pyrrolidine-1-carboxylate) (**14**)

To a stirred solution of compound **13** (250 mg, 0.50 mmol, 1.0 equiv) in dry DMF (4 mL) under nitrogen atmosphere, acryloyl chloride (68 mg, 60 μL, 0.75 mmol, 1.5 equiv) and Cs_2_CO_3_ (487 mg, 1.50 mmol, 3.0 equiv) were added. The reaction mixture was stirred at room temperature overnight. Afterward, AcOEt was added, and the reaction mixture was washed with water (×3) and brine (×1). The organic phase was dried over anhydrous Na_2_SO_4_, filtered and evaporated under reduced pressure. Purification by preparative TLC (CH_2_Cl_2_/MeOH 98:2) afforded compound **14** as a yellow solid (222 mg, 0.25 mmol, 50% yield). ^1^H NMR (300 MHz, CDCl_3_) δ 9.44 (br, 1H), 8.50 (d, *J* = 9.0 Hz, 1H), 7.72 (s, 1H), 7.63 (s, 1H), 7.57 (s, 1H), 7.50–7.42 (m, 2H), 7.31 (d, *J* = 2.4 Hz, 1H), 7.07 (dd, *J* = 9.0, 2.4 Hz, 1H), 7.01–6.90 (m, 3H), 6.68 (dd, *J* = 16.7, 1.5 Hz, 1H), 6.05 (dd, *J* = 10.4, 1.5 Hz, 1H), 4.51–4.36 (m, 1H), 3.96 (s, 3H), 3.56–3.32 (m, 2H), 2.56–2.42 (m, 1H), 1.98–1.83 (m, 3H), 1.48 (s, 9H).

#### *tert*-Butyl-(*S*,*E*)-2-((4-((1-(but-2-enoyl)-3-(1-methyl-1*H*-pyrazol-4-yl)-1*H*-indol-5-yl)­oxy)­phenyl)­carbamoyl)­pyrrolidine-1-carboxylate (**15**)

To a stirred solution of **13** (100 mg, 0.20 mmol, 1.0 equiv) in dry CH_2_Cl_2_ (3 mL) under nitrogen atmosphere, tetrabutylammonium hydrogen sulfate (0.1 equiv) and finely powdered sodium hydroxide (5.0 equiv) were added. The mixture was stirred for 15 min and then crotonyl chloride (57 μL, 0.6 mmol, 3.0 equiv) was added. The reaction mixture was stirred for 3 h at room temperature. Then, water was added, and the aqueous phase was extracted with CH_2_Cl_2_ (×3). The combined organic layers were washed with 1 M NaOH solution (×1), and then dried over anhydrous Na_2_SO_4_, filtered and evaporated under reduced pressure. Purification by flash column chromatography (CH_2_Cl_2_ 100% and then CH_2_Cl_2_/MeOH 99:1) on silica gel afforded compound **15** as a yellow solid (21 mg, 0.037 mmol, 18% yield). ^1^H NMR (300 MHz, CDCl_3_) δ 9.43 (s, 1H), 8.50 (d, *J* = 9.0 Hz, 1H), 7.73 (s, 1H), 7.64 (s, 1H), 7.60 (s, 1H), 7.50–7.42 (m, 2H), 7.34–7.25 (m, 2H), 7.08 (dd, *J* = 8.9, 2.4 Hz, 1H), 7.00–6.92 (m, 2H), 6.71 (dq, *J* = 15.0, 1.7 Hz, 1H), 4.51–4.40 (m, 1H), 3.97 (s, 3H), 3.54–3.30 (m, 2H), 2.60–2.46 (m, 1H), 2.07 (dd, *J* = 6.9, 1.7 Hz, 3H), 2.01–1.86 (m, 3H), 1.49 (s, 9H).^13^C NMR (75 MHz, CDCl_3_) δ 169.78, 163.64, 153.73, 146.88, 137.59, 132.69, 132.15, 132.01, 130.50, 128.59, 128.42, 127.67, 122.04, 121.22, 118.51, 118.14, 117.38, 114.66, 113.91, 109.59, 80.92, 77.24, 60.34, 47.23, 39.13, 29.69, 28.40, 18.65.

#### *tert*-Butyl (*S*)-2-((4-((1-(3-Bromopropanoyl)-3-(1-methyl-1*H*-pyrazol-4-yl)-1*H*-indol-5-yl)­oxy)­phenyl)­carbamoyl)­pyrrolidine-1-carboxylate (**16**)

To a stirred solution of **13** (328 mg, 0.65 mmol, 1.0 equiv) in dry CH_2_Cl_2_ (15 mL) under nitrogen atmosphere, 8-diazabiciclo[5.4.0]­undec-7-ene (DBU, 496 mg, 490 μL, 3.27 mmol, 5.0 equiv) was added. The reaction mixture was cooled at 0 °C and bromo acetyl bromide (396 mg, 170 μL, 1.96 mmol, 3.0 equiv) was added. The reaction mixture was stirred at room temperature for 2 h. After completion of the reaction, the solvent was evaporated under reduced pressure. Purification by column chromatography on silica gel (cyclohexane/AcOEt from 4:6 to 1:9) afforded compound **16** as a yellow solid (190 mg, 0.31 mmol, 47% yield). ^1^H NMR (300 MHz, DMSO-d6) δ 9.93 (s, 1H), 8.36–8.30 (m, 1H), 8.26 (s, 1H), 8.20 (s, 1H), 7.84 (s, 1H), 7.58–7.52 (m, 3H), 7.07–7.04 (m, 1H), 6.95 (d, *J* = 12.5 Hz, 1H), 4.82 (s, 2H), 4.24–4.13 (m, 1H), 3.87 (s, 3H), 3.38 −3.33 (m, 2H), 2.21–2.12 (m, 1H), 1.87–1.74 (m, 3H), 1.33 (s, 9H).

#### *tert*-Butyl-(*S*)-2-((4-((3-(1-methyl-1*H*-pyrazol-4-yl)-1-propionyl-1*H*-indol-5-yl)­oxy)­phenyl)­carbamoyl)­pyrrolidine-1-carboxylate (**17**)

To a stirred solution of **13** (375 mg, 0.75 mmol, 1.0 equiv) in dry DMF (7 mL) under nitrogen atmosphere, Cs_2_CO_3_ (0.73 g, 2.25 mmol, 3.0 equiv) and propionyl chloride (98 μL, 1.12 mmol, 1.5 equiv) were added. The mixture was stirred overnight at room temperature. Afterward, AcOEt was added, and the reaction mixture was washed with brine (×1). The organic phase was dried over anhydrous Na_2_SO_4_, filtered and evaporated under reduced pressure. Purification by column chromatography (cyclohexane/AcOEt 9:1, then 6:4 until 100% AcOEt) allowed to obtain compound **17** as a yellow solid (138 mg, 0.25 mmol, 33% yield). ^1^H NMR (300 MHz, CDCl_3_) δ 9.44 (br, 1H), 8.48 (d, *J* = 9.0 Hz, 1H), 7.72 (s, 1H), 7.64 (s, 1H), 7.53 (s, 1H), 7.48–7.43 (m, 2H), 7.31 (d, *J* = 2.4 Hz, 1H), 7.08 (dd, *J* = 9.0, 2.4 Hz, 1H), 6.98–6.92 (m, 2H), 4.51–4.39 (m, 1H), 3.97 (s, 3H), 3.54–3.31 (m, 2H), 2.98 (q, *J* = 7.4 Hz, 2H), 2.64–2.45 (m, 1H), 2.01–1.87 (m, 3H), 1.49 (s, 9H), 1.37 (t, *J* = 7.4 Hz, 3H). ^13^C NMR (75 MHz, CDCl_3_) δ 171.69, 169.94, 156.41, 154.18, 153.57, 137.56, 133.64, 132.48, 130.20, 127.68, 121.24, 121.04, 118.43, 117.84, 117.49, 114.72, 113.86, 109.60, 80.85, 77.27, 60.42, 47.23, 39.11, 29.02, 28.40, 24.60, 8.71.

### General Procedure 1 for Boc-Deprotection

To a stirred solution of dry MeOH (2.5 mL) at 0 °C, acetyl chloride was added dropwise (0.4 mL) under nitrogen atmosphere. The mixture was stirred at room temperature for 5 min. After this time, the solution (6.0 equiv) was added to the appropriate *N*-Boc-protected compound (1.0 equiv). The reaction mixture was stirred at room temperature under nitrogen atmosphere for 1 h. After completion of the reaction, the mixture was evaporated under reduced pressure to obtain the desired final compounds.

#### (*S*)-*N*-(4-((1-Acryloyl-3-(1-methyl-1*H*-pyrazol-4-yl)-1*H*-indol-5-yl)­oxy)­phenyl)­pyrrolidine-2-carboxamide Hydrochloride (**5**)

Compound **5** was prepared according to general procedure 1 from compound **14** (70 mg, 0.13 mmol, 1.0 equiv), using 362 μL of dry methanol/acetyl chloride solution. The desired compound was afforded as a yellow solid (48 mg, 0.09 mmol, 70% yield). ^1^H NMR (300 MHz, CD_3_OD) δ 8.52 (d, *J* = 9.0 Hz, 1H), 8.08 (s, 1H), 8.04 (s, 1H), 7.86 (s, 1H), 7.60–7.54 (m, 2H), 7.41 (d, *J* = 2.3 Hz, 1H), 7.29 (dd, *J* = 16.6, 10.5 Hz, 1H), 7.05 (dd, *J* = 9.0, 2.4 Hz, 1H), 7.02–6.97 (m, 2H), 6.65 (dd, *J* = 16.6, 1.5 Hz, 1H), 6.09 (dd, *J* = 10.5, 1.5 Hz, 1H), 4.43–4.31 (m, 1H), 3.95 (s, 3H), 2.60–2.42 (m, 2H), 2.21–2.01 (m, 4H). ^13^C NMR (75 MHz, CD_3_OD) δ 166.27, 163.74, 154.88, 153.94, 134.63, 132.87, 132.46, 131.66, 131.15, 129.84, 127.77, 123.41, 121.58, 118.21, 117.79, 116.86, 114.97, 112.62, 109.34, 60.36, 46.25, 37.87, 29.89, 23.85. HRMS (ESI) calcd for C_26_H_25_N_5_O_3_ [M + H]^+^ *m*/*z*: 456.2036; found: 456.2045. HPLC: *t*_R_ = 6.366 min.

#### (*S,E*)-1-(3-(1-Methyl-1*H*-pyrazol-4-yl)-5-(4-((pyrrolidin-2-ylmethyl)­amino)­phenoxy)-1*H*-indol-1-yl)­but-2-en-1-one Hydrochloride (**6**)

Compound **6** was prepared according to general procedure 1 from compound **15** (31 mg, 0.05 mmol, 1.0 equiv), using 158 μL of dry methanol/acetyl chloride solution. The desired compound was afforded as a yellow solid (27 mg, 0.05 mmol, quantitative yield). ^1^H NMR (300 MHz, CD_3_OD) δ 8.46 (d, *J* = 9.0 Hz, 1H), 8.04 (s, 1H), 8.01 (s, 1H), 7.85 (s, 1H), 7.60–7.54 (m, 2H), 7.36 (d, *J* = 2.4 Hz, 1H), 7.31–7.18 (m, 1H), 7.04–6.92 (m, 4H), 4.46–4.37 (m, 1H), 3.94 (s, 3H), 3.51–3.33 (m, 2H), 2.60–2.47 (m, 1H), 2.16–2.08 (m, 3H), 2.05 (dd, *J* = 6.9, 1.3 Hz, 3H). ^13^C NMR (75 MHz, CD_3_OD) δ 166.22, 164.11, 155.06, 153.72, 146.84, 136.62, 132.76, 132.63, 130.32, 128.72, 122.21, 121.84, 121.54, 118.18, 117.70, 116.53, 114.15, 113.95, 109.27, 60.31, 46.12, 37.57, 29.72, 23.74, 17.17. HRMS (ESI) calcd for C_27_H_27_N_5_O_3_ [M + H]^+^ *m*/*z*: 470.2192; found: 470.2203. HPLC: *t*_R_ = 6.163 min.

#### (*S*)-*N*-(4-((1-(3-Chloropropanoyl)-3-(1-methyl-1*H*-pyrazol-4-yl)-1*H*-indol-5-yl)­oxy)­phenyl)­pyrrolidine-2-carboxamide Hydrochloride (**7**)

Compounds **7** was prepared according to general procedure 1 from **16** (50 mg, 0.08 mmol, 1.0 equiv), using 230 μL of the solution dry methanol/acetyl chloride. The desired compound was afforded as a yellow solid (27 mg, 0.05 mmol, 61% yield). ^1^H NMR (300 MHz, CD_3_OD) δ 8.42 (d, *J* = 9.0 Hz, 1H), 8.22 (s, 1H), 8.08 (s, 1H), 8.00 (s, 1H), 7.61–7.55 (m, 2H), 7.42 (d, *J* = 2.3 Hz, 1H), 7.05 (dd, *J* = 9.0, 2.4 Hz, 1H), 7.01–6.95 (m, 2H), 4.90 (s, 2H), 4.47–4.38 (m, 1H), 4.02 (s, 3H), 3.49–3.37 (m, 2H), 2.18–2.05 (m, 4H). ^13^C NMR (101 MHz, CD_3_OD) δ 166.36, 165.03, 154.94, 154.19, 135.52, 132.92, 132.46, 130.18, 129.82, 122.57, 121.57, 118.27, 117.45, 117.06, 114.40, 113.98, 109.47, 60.32, 46.12, 42.25, 37.56, 29.74, 23.74. HRMS (ESI) calcd for C_25_H_25_ClN_5_O_3_ [M + H]^+^ *m*/*z*: 478.1646; found: 478.1649. HPLC: *t*_R_ = 6.014 min.

#### (*S*)-*N*-(4-((3-(1-Methyl-1*H*-pyrazol-4-yl)-1-propionyl-1*H*-indol-5-yl)­oxy)­phenyl)­pyrrolidine-2-carboxamide Hydrochloride (**5r**)

Compound **5r** was prepared according to general procedure 1 from compound **17** (70 mg, 0.13 mmol, 1.0 equiv), using 362 μL of dry methanol/acetyl chloride solution. The desired compound was afforded as a light-yellow solid (52 mg, 0.10 mmol, 77% yield). ^1^H NMR (300 MHz, CD_3_OD) δ 8.37 (d, *J* = 8.9 Hz, 1H), 7.90 (s, 1H), 7.82 (s, 1H), 7.75 (s, 1H), 7.54 (d, *J* = 7.2 Hz, 2H), 7.30 (d, *J* = 2.1 Hz, 1H), 6.95 (m, 3H), 4.28–4.10 (m, 1H), 3.24 (m, 1H), 2.99 (q, *J* = 7.3 Hz, 2H), 2.42 (m, 1H), 2.00 (br s, 3H), 1.27 (t, *J* = 7.3 Hz, 4H). ^13^C NMR (75 MHz, CD_3_OD) δ 172.58, 154.95, 153.48, 149.85, 136.88, 132.83, 132.40, 130.01, 128.33, 121.97, 121.53, 118.11, 117.31, 117.01, 116.55, 114.02, 112.09, 109.19, 60.43, 37.56, 30.14, 28.27, 7.61. HRMS (ESI) calcd for C_26_H_27_N_5_O_3_ [M + H]^+^ *m/z:* 458.2192; found: 458.2183. HPLC: *t*_R_ = 5.956 min.

### Continuous-Flow Acylation of Indole Intermediate **13**

Continuous flow biotransformations were performed using a R2^+^/R4 Vaportec flow reactor equipped with an Omnifit glass column (10 mm i.d. × 150 mm length). The temperature sensor sits on the wall of the reactors. Pressure was controlled by using a 20 psi back-pressure regulator. For the in-line extraction, an additional HPLC pump (ThalesNano) was used. In-line liquid/liquid separation was performed using a Zaiput separator. Amberlite 900 (^−^OH) was prepared from commercially available Amberlite 900 (Cl^–^) by ion exchange. The chloride-form resin was placed in a beaker and treated with 1 M NaOH for 15 min under stirring; the basic solution was decanted and replaced with fresh 1 M NaOH and stirred for an additional 15 min. The resin was then filtered under vacuum, washed with water followed by THF.

General procedure for the synthesis of *tert*-butyl (*S,E*)-2-((4-((1-(but-2-enoyl)-3-(1-methyl-1*H*-pyrazol-4-yl)-1*H*-indol-5-yl)­oxy)­phenyl)­carbamoyl)­pyrrolidine-1-carboxylate (**15**) and *tert*-butyl (*S*)-2-((4-((3-(1-methyl-1*H*-pyrazol-4-yl)-1-propionyl-1*H*-indol-5-yl)­oxy)­phenyl)­carbamoyl)­pyrrolidine-1-carboxylate (**17**).

An Omnifit glass column (10 mm i.d. × 150 mm length) was packed with Amberlite 900 (−OH) ion-exchange resin (volume: 3.2 mL) and washed with dry CH_2_Cl_2_ (flow rate: 0.50 mL min^–1^ for 15 min). A 0.10 M solution of starting material (0.40 mmol, 1.0 equiv) was prepared in dry CH_2_Cl_2_ (4.0 mL). To this solution tetrabutylammonium hydrogen sulfate (13.5 mg, 0.04 mmol, 0.10 equiv) was added. A 0.15 M solution of the appropriate acyl chloride (0.60 mmol, 1.5 equiv) was prepared in dry CH_2_Cl_2_ (4.0 mL). The reaction was performed at room temperature with a residence time of 15 min and a total flow rate of 0.21 mL min^–1^. The exiting flow stream was extracted in-line by adding an inlet of NaOH pumped with an external HPLC pump at 0.21 mL min^–1^. The outlet flow was directed to a liquid/liquid separator. The organic phase was dried over anhydrous Na_2_SO_4_, filtered, and concentrated under reduced pressure. Crude material was purified by column chromatography (CH_2_Cl_2_/MeOH, from 99:1 to 98:2) to obtain the desired compound (**15**: 56% yield; **17**: 45% yield).

### Molecular Protein–Ligand Docking

Docking simulations were conducted using AutoDock 4.2.6.^49^ The atomic charges, torsional flexibility, and protonation states of the enzyme and of the ligands were set up using, respectively, the AutoDock Tools graphical interface and Meeko (https://meeko.readthedocs.io). The ligand binding site for performing the docking calculation was identified on the basis of the ligand’s position in the original PDB structure, i.e., its geometric center has been used to center the docking box for all the systems considered in the present work. The docking grid was set to 52 × 52 × 52 Å^3^ along the *x*, *y*, and *z* axes, with a grid spacing of 0.375 Å. Docking simulations employed a genetic algorithm, with each configuration file corresponding to a single docking run, generating up to 100 possible binding poses. For each pose the binding free energy has been computed as a sum of five contributions, i.e., van der Waals (a 12–6 Lennard-Jones potential), hydrogen bonding (a 12–10 potential), electrostatics Coulomb potential, desolvation energy (associated with solvent displacement) and torsional entropy (a penalty based on the number of rotatable bonds).^49^ These poses were then statistically analyzed based on their predicted free binding energy and the corresponding binding affinity expressed in terms of dissociation constant (*K*_i_).

### MD Simulations

The best docking poses in terms of *K*_*i*_ and free binding energy for the complex of PFKFB3 with the ligands were considered as starting structures for MD simulations. For each ligand, two poses were selected, i.e., pose 1, where the ligand overlaps with the compound in the crystal structure; pose 2, where the ligand is approximately rotated by 180° compared to the reference conformation. The parametrization of all the ligands for the MD simulations was carried out through Self Consistent Field (SCF) Restricted Hartree–Fock (RHF) calculations performed in NWChem^50^ as implemented in the BiKi suite.^51^ The molecular wave function and the corresponding electrostatic potential of the molecules were computed using a 6-31G* basis set. The RESP (Restrained ElectroStatic Potential) algorithm was applied to the QM-calculated electrostatic potential (ESP) at molecular surfaces using an atom-centered point charge model. The molecular charges and the interatomic forces have been translated using the Generalized Amber Force field (GAFF) into an AMBER99SB-ILDN^52^ compatible molecular mechanics topology for the GROMACS package.^53^ Covalently bound ligands have been quantum-chemically parametrized with the same methodology reported above, i.e., in the form of a ligand and a cysteine with a close proximity between, respectively, β-carbon and γ-sulfur atoms and a protonation state compatible with the formation of the covalent S–C bond. The corresponding molecular mechanics topology has been integrated in the AMBER99SB-ILDN topology for the GROMACS package. A cubic solvent box was generated around all the systems, solvated with water TIP3P molecules and the electrostatics treated with the Particle Mesh Ewald (PME) scheme. After minimization with the steepest descent method (convergence: 100 kJ mol^–1^ nm^–1^), the system was equilibrated with isotropic positional restraints on protein heavy atoms (*k* = 1000 kJ mol^–1^ nm^–2^) for 2 ns in the NPT ensemble with *p* = 1 atm and *T* = 310 K, then for 2 ns in the NVT ensemble at *T* = 310 K. The equilibrated system configurations were then used for running 100 ns MD simulations at 310 K.

### Intrinsic Warhead Reactivity Using βME

The reactivity of **5**–**7** and ibrutinib with the model thiol βME^34^ was evaluated by mass spectrometry using an Agilent LC/MSD XT mass spectrometer (Agilent Technologies, Santa Clara, CA, USA). Stock solutions (5 mM) of each compound were prepared in DMSO. Reaction mixtures were assembled by combining 2 μL of compound stock solutions (final concentration: 100 μM), 95 μL of 10 mM ammonium acetate buffer at pH 7.0, and 3 μL of 2 M βME (final concentration: 60 mM) in HPLC vials. The mixtures were incubated at 25 °C, and aliquots were withdrawn over time. For analysis, 2 μL of each reaction mixture was injected into the mass spectrometer using a mobile phase consisting of H_2_O:acetonitrile:methanol (40:30:30, v/v/v), at a flow rate of 0.2 mL/min and a column temperature of 25 °C. The extent of adduct formation and compound depletion was monitored over time (5–20 min) by detecting the corresponding [M + H]^+^ signals. The relative intensities of the unmodified compound and its βME adduct were used to estimate the percentage of each species at each time point. Assuming pseudo-first-order kinetics with respect to the electrophile, the time-dependent formation of the adduct and disappearance of the parent compound were fitted to single-exponential decay. Reaction rate constants were extracted from the fits using nonlinear regression.

### Expression and Purification of PFKFB Isoforms and C154S PFKFB3

The gene encoding human PFKFB3 (EC 2.7.1.105), isoform 1 (NCBI RefSeq: NP_004557.1, UniProt: Q16875), PFKFB1 (UniProt: P16118), PFKFB2 (UniProt. O60825), and PFKFB4 (UniProt: Q16877), in frame with an *N*-terminal hexa-histidine (His_6_) tag, were subcloned into the pET28a­(+) expression vector between the NcoI and *Bam*HI restriction sites (GenScript, Piscataway, NJ, USA). The C154S variant of PFKFB3 was generated from the same construct by site-directed mutagenesis. All plasmids were transformed into *Escherichia coli* BL21­(DE3) cells (Thermo Fisher Scientific, Waltham, MA, USA). Expression was induced with 1 mM isopropyl-β-D-1-thiogalactopyranoside (IPTG) at 20 °C for 20 h. The cell paste was recovered by centrifugation, extensively washed and resuspended in 25 mM Tris, 300 mM NaCl, 1 mM dithiothreitol (DTT), 1.5 μM pepstatine A, 0.2 mM benzamidine, 0.2 mM phenylmethylsulfonyl fluoride (PMSF), 1 mg/mL lysozyme, pH 7.8. Cells were lysed by sonication. Proteins were purified by standard immobilized metal affinity chromatography (IMAC) using Talon Superflow resin (Cytiva, Marlborough, MA, USA), followed by dialysis against 50 mM HEPES, 200 mM NaCl, 0.2 mM EDTA, pH 7.5. Purified proteins were concentrated, flash-frozen in liquid nitrogen, and stored at −80 °C in 50 μL aliquots at concentrations of 120–150 μM. Protein purity was assessed by 12% SDS-PAGE followed by densitometric analysis using a ChemiDoc system (Bio-Rad, Hercules, CA, USA). Protein concentrations were estimated by UV–visible absorbance at 280 nm with a Cary 4000 UV–vis spectrophotometer (Agilent Technologies, Santa Clara, CA, USA), using the extinction coefficients 60,125 M^–1^·cm^–1^ for PFKFB3, 65,210 M^–1^·cm^–1^ for PFKFB1, 68,230 M^–1^·cm^–1^ for PFKFB2, and 57,760 M^–1^·cm^–1^ for PFKFB4 (calculated with ProtParam, *ExPASy Bioinformatics Resource Portal*). Before each experiment, the aliquots were thawed, extensively centrifuged and the concentration of protein was redetermined.

The oligomerization state of PFKFB3 was analyzed by size-exclusion chromatography (SEC) using an Agilent 1260 Infinity HPLC system (Agilent Technologies, Santa Clara, CA, USA) equipped with a Superdex 200 Increase 3.2/300 GL column (Cytiva, Marlborough, MA, USA). The column was equilibrated at 30 °C and developed in phosphate-buffered saline (PBS) at a constant flow rate of 0.07 mL/min. A calibration curve was generated using the following molecular weight standards: apoferritin (440 kDa), aldolase (140 kDa), conalbumin (75 kDa), and myoglobin (17 kDa). PFKFB3 was loaded at 33 μM concentration.

### Enzyme-Coupled Assay

PFKFB1–4 and C154S PFKFB3 enzyme activities were measured using a coupled assay in which the ADP generated by the phosphofructokinase reaction in the presence of fructose 6-phosphate (F6P) and ATP is converted back to ATP by PK in the presence of phosphoenolpyruvate (PEP). The resulting pyruvate is then reduced by LDH, with concomitant oxidation of NADH to NAD^+^, causing a decrease in absorbance at 340 nm (PMID: 31475972). The reaction mixture (150 μL) was prepared in a buffer containing 10 mM sodium phosphate and 50 mM NaCl, pH 7.5, with the following components at their final concentrations: either PFKFB isoforms or C154S PFKFB3 at 2.5 μM, F6P at 2 mM, PEP at 1 mM, NADH at 300 μM, LDH (L2625-12.5 KU, Merck, Darmstadt, Germany) at 53 U/mL and PK (S-P7768-2.5 KU, Merck, Darmstadt, Germany) at 25 U/mL. Detergents—either 0.2% Tween 20 or 0.03% Brij-30— were added as specified for selected experiment. The reaction was initiated by the addition of 1 mM ATP. Absorbance changes were monitored at 340 nm using a Varian Cary 4000 UV–vis spectrophotometer (Agilent Technologies, Santa Clara, CA, USA). To determine the enzymatic kinetics of PFKFB3 and C154S PFKFB3, the ATP concentration was varied at a fixed F6P concentration (1 mM), and F6P concentration was varied at a fixed ATP concentration (0.6 mM). *K*_m_ and *V*_max_ were obtained by fitting the data points to the Michaelis–Menten equation. To assess inhibition in a cell lysate, Mia Paca-2 cell lysates (5·10^6^ cells per batch) were obtained by 5 freeze–thaw cycles in a buffer containing 20 mM sodium phosphate, 100 mM NaCl, 0.2% Tween 20, pH 7.5. Upon centrifugation, the protein content was assessed by Bradford assay. All activity assays were performed at least in triplicate and at 37 °C.

### Enzyme Inhibition Evaluated by Enzyme Assays

Stock solutions of **4**–**7** and **5r** in DMSO were prepared at 50 mM concentration. The coupled assay was conducted on PFKFB3 and C154S PFKFB3 in the presence or absence of test compounds at final concentrations ranging from 0.5 to 10 μM, as specified per experiment. Control assays contained equivalent DMSO concentrations. Since **5** and **7** caused precipitation of PFKFB3, Tween 20 or Brij-30 were added to the assay mixture when necessary. To determine the binding stoichiometry of irreversible inhibitors, concentrations from 20 to 200% of that of the protein (2.5 μM) were tested. For the determination of IC_50_ values of reversible inhibitor **4**, the assay was adapted to a HaloLED 96-well microplate reader (Control Tecnica, Padova, Italy). The enzyme concentration was adjusted to 50 nM, and inhibitor concentrations varied from 50 nM to 10 μM.

The IC_50_s of reversible inhibitors were determined by fitting the activity data collected at different concentrations of inhibitors by nonlinear regression using a logistic model, with the sigmoidal dose–response expressed as a function of the logarithm of the concentration curve. Assuming competitive inhibition of the ligand (L) at the ATP binding site, the Cheng-Prusoff equation^54^ (eq 1) was applied to estimate the corresponding *K_i_*.

1 $$K_{i}^{L}=\frac{{\mathrm{IC}}_{50}^{L}}{1+\frac{\left[\mathrm{ATP}\right]}{1+K_{m}^{\mathrm{ATP}}}}$$

To determine the binding parameters of irreversible inhibitors, the residual enzyme activities measured at different concentrations of **6** and at different time points were analyzed with exponential decays to yield the observed rate constants *k*_obs_, which were then analyzed with the Kitz–Wilson model (eq 2) to yield the inactivation rate constant *k*_inact_ and the inhibition constant *K*_*I*_.

2 $$k_{\mathrm{obs}}=\frac{k_{\mathrm{inact}}\left[I\right]}{K_{I}+\left[I\right]}$$

When *k*_inact_ is not negligible relative to *k*_off_—the dissociation rate constant of the enzyme–inhibitor complex—*K*_*I*_ must be treated as an apparent equilibrium constant and is formulated as in eq 3.

3 $$K_{I}=\frac{k_{\mathrm{off}}+k_{\mathrm{inact}}}{k_{\mathrm{on}}}$$

Under these conditions, *K*_*I*_ deviates from the true equilibrium constant *K*_*i*_ as derived from equilibrium rather than kinetic measurements (eq 4).

4 $$K_{i}=\frac{k_{\mathrm{off}}}{k_{\mathrm{on}}}$$

### Evaluation of Irreversible Binding by TFA-mediated Co-Precipitation Assays and Dialysis

To assess the binding between **5**–**7** with PFKFB3 and C154S PFKFB3, a trifluoroacetic acid (TFA)-induced coprecipitation assay^55^ was performed, taking advantage of an absorption band of 323 nm for **6** (ε = 6976 M^–1^ cm^–1^), 314 nm for **5** (ε = 12211 M^–1^ cm^–1^), 312 nm for **7** (ε = 9478 M^–1^ cm^–1^). Either PFKFB3 or C154S PFKFB3 (25 μM) were incubated with 16.5 μM of the compounds in a buffer containing 10 mM sodium phosphate, 50 mM NaCl, pH 7.5 for 30 min at 25 °C. For **5** and **7**, 0.03% Brij-30 was added. TFA was then added to a final concentration of 10% (v/v) to induce protein denaturation and the samples were centrifuged at 16,000*g* for 15 min to remove protein precipitate. The residual concentration of the compounds in solution was assessed by UV–visible absorption spectra. The residual protein concentration was measured by UV–vis absorbance at 280 nm. The reaction kinetics were estimated using the same approach, by sampling the reaction mixture for up to 30 min. To further assess irreversible binding through an activity-based approach, a dialysis experiment was conducted. PFKFB3 (2.5 μM) was incubated with the test compounds at 10 μM for 30 min at 25 °C, followed by dialysis for 3 h against 10 mM sodium phosphate, 50 mM NaCl, and 0.2% Tween 20, pH 7.5 to reduce free inhibitor concentration to below 0.15 μM (or 6% of the enzyme concentration). The residual enzyme activity was then assayed as described above.

### Evaluation of the Kinase Activity Through an NMR Assay

The ATP consumption and ADP formation due to the enzymatic activity of PFKFB3 were monitored acquiring ^1^H NMR spectra and evaluating the area under the peak related to the purine H8 proton.^56^ Spectra were acquired on a JEOL 600 MHz spectrometer at 25 °C. The reaction mixture (600 μL) contained 2.5 μM PFKFB3, 200 μM ATP, 2 mM F6P, 10 mM MgCl_2_, 50 mM NaCl, 10 mM sodium phosphate buffer, pH 7.5 with 10% D_2_O. The ATP concentration was optimized to achieve a sufficient signal-to-noise ratio while minimizing peak overlap. For the experiment in the presence of **6**, a final concentration of 12.5 μM was added. A water suppression excitation sculpting pulse sequence was applied to remove the water signal. A total of 64 scans with a spectral width of 14 ppm were acquired for each experiment. The first spectrum was acquired containing all components but the substrate F6P. Then the substrate was added and, after proper shimming, spectra were acquired every 5 min. Peaks related to ATP and ADP purine H8 were integrated to monitor the changes over time. Fixed integration limits were applied consistently across samples. Spectra were analyzed using the Reaction monitoring wizard of MestReNova, version 14.2.0 (Mestrelab Research S.L., Santiago de Compostela, Spain).

### Evaluation of Cysteine Selectivity by LC-MS/MS

To evaluate covalent binding of specific cysteine residues, PFKFB3 was reacted with **6** for 30 min at 25 °C. The reaction was then quenched with 1 mM cysteine, and the mixture was precipitated overnight in acetone at −20 °C. The pellet was resuspended in 50 mM Tris buffer with 8 M urea, pH 8.0, reduced with 5.5 mM Tris­(2-carboxyethyl)­phosphine (TCEP) for 2 h at 30 °C, and alkylated with 10 mM iodoacetamide for 30 min at 25 °C in the dark. The solution was diluted to 2 M urea with 50 mM Tris, pH 8.0, and digested with trypsin overnight at 30 °C. The reaction was stopped by adding 0.1% trifluoroacetic acid (TFA). A control sample without **6** underwent the same procedure. LC-MS/MS analysis was performed using an Exploris 480 Hybrid Quadrupole-Orbitrap Mass Spectrometer (Thermo Fisher Scientific, USA) coupled to a Thermo Ultimate 3000 Nano UHPLC. The sample was analyzed in triplicates, using 1 μg per injection. Chromatographic separation was performed on a 75 μm × 500 mm Easy Nano C18 column (ES903, Thermo Scientific, USA). An Easy-Spray source was operated in positive mode with the following parameters: spray voltage 1.8 kV; ion transfer tube temperature 275 °C; sheath gas 3; auxiliary gas 8; sweep gas 0. Chromatographic separation was performed at 45 °C using a flow rate of 0.25 mL/min. The system was equilibrated with 96% solvent A (0.1% formic acid in water with 3% acetonitrile) and 4% solvent B (acetonitrile with 0.1% formic acid and 3% water). Two minutes postinjection, solvent B was linearly increased from 4% to 26% over 50 min, then to 50% from 52–60 min, to 95% in 2 min, held for 3.9 min, and returned to 4% in 0.1 min for re-equilibration. Total run time was 120 min per sample. MS and MS/MS spectra were acquired in Full MS/dd-MS^2^ (TOPN) mode (*m*/*z* 200–3000), with resolution set at 120,000 (MS1) and 15,000 (MS2). The 20 most intense ions were selected for nitrogen-promoted HCD fragmentation (NCE = 28). Dynamic exclusion was set to 55 s; charge inclusion range: 2–6. The instrument was automatically calibrated before analysis. Raw data were analyzed using MaxQuant v2.6.7.0 ^57^ with the Andromeda search engine, searching against the sequence of PFKFB3, decoy sequences and known contaminants. Peptides were identified with a minimum length of 7 residues, applying a 1% FDR at the PSM, protein, and site levels. Variable modifications included oxidation (M), N-terminal acetylation, carbamidomethylation (C) and a custom modification corresponding to the predicted adduct with **6**, (+469.21 Da). To confidently identify peptides covalently modified by **6**, MaxQuant output data were filtered using the following criteria: Posterior Error Probability (PEP) < 0.01, site localization probability > 0.75, localization score > 40, delta score > 10, and precursor ion intensity > 1·10^8^. Peptides harboring multiple potential modification sites were excluded. The same protocol was applied to PFKFB3 nonincubated with **6**.

### Analysis of PFKFB3 Expression in Public RNA Sequencing Data Sets

PFKFB3 expression was evaluated using both bulk and single-cell RNA sequencing data. For single-cell analysis, expression levels were assessed in healthy and tumor pancreatic samples using the Pancreatic Tissue Single Cell Atlas (https://pascadimaglianolab.shinyapps.io/SC_Pancreas_Atlas/, accessed November 2025). Data from six healthy donors were integrated with previously published tumor (*n* = 16) samples. Feature matrices of scRNA-seq data are publicly available from the NIH Gene Expression Omnibus (GEO) under accession numbers GSE229413 and GSE155698.

For bulk RNA-seq analysis, PFKFB3 expression in pancreatic adenocarcinoma (PDAC) was examined using the GEPIA2 database (http://gepia.cancer-pku.cn/detail.php?gene=PFKFB3, accessed November 2025), which integrates TCGA and GTEx data sets. Expression levels were calculated as log_2_ (TPM + 1), and comparisons were made between tumor and normal pancreatic tissues. Log_2_ fold-change and p-value thresholds were set according to GEPIA default settings.

The disease-free survival (*n* = 150) and overall survival of PDAC patients (*n* = 375) were analyzed using KM plotter database (https://pancreas.kmplot.com/, accessed in November 2025). The patients were split by trichotomization. Then, the overall survival Kaplan–Meier plot was plotted also after stratifying PDAC patient for tumor grade (G2, G3, G4). The statistical significance between groups was evaluated using the log-rank test. Hazard ratios (HRs) with 95% confidence intervals were calculated and reported where applicable.

### Cell Culture

Human PDAC cell lines (PANC-1, MIA PaCa-2, AsPC-1, Hs 766T, PaCa-3, and SUIT-2), together with human pancreatic ductal epithelial cells (HPDE) and human fibroblasts, were cultured in RPMI 1640 medium (Gibco, Cat. No. 52400025) supplemented with 10% fetal bovine serum (FBS; Gibco, Cat. No. A5256701) and 50 μg/mL gentamicin (Gibco, Cat. No. 15750037). AsPC-1, PANC-1 and human fibroblast were purchased from the American Type Culture Collection (ATCC). SUIT-2 and CFPAC were kindly provided by Prof. S. Ugel (University of Verona, Italy), while PaCa3, Hs776t and HPDE1 cells were obtained from Prof. A. Scarpa (University of Verona, Italy). Additional human cancer and normal cell lines were maintained in their appropriate basal media with standard penicillin/streptomycin supplementation. A549 lung cancer cells, WI-38 lung fibroblasts and 293T embryonic kidney cells were grown in DMEM, while CALU-3 lung cancer cells, ACHN kidney cancer cells, U251 glioblastoma cells, BJ foreskin fibroblasts and SH-SY5Y neuroblastoma cells were maintained in MEM; SH-SY5Y cells were grown in a 1:1 mixture of MEM and Ham’s F-12. MEM-based cultures were supplemented with 10% FBS (Euroclone, Cat. No. ECS0165L or CHA1115L), 2 mM l-Glutamine (Euroclone, Cat. No. ECB3000D), 1 mM sodium pyruvate (Euroclone, Cat. No. ECM0542D) and 0.1 mM nonessential amino acids (Euroclone, Cat. No. ECB3054D). BJ cell line was obtained from America Type Culture Collection (ATCC); ACHN cell line was obtained from Cell Line service (CLS); U251 cell line was obtained from Interlab Cell Line Collection (ICLC); SH-SY5Y cell line was obtained from European Collection of Authenticated Cell Cultures (ECACC); 293T cell line was provided by Prof. L. Naldini (TIGET San Raffaele Milano, Italy); A549 and WI38 cell lines were imported from Prof. P.G. Pelicci (from University of Perugia, Italy).

All cells were incubated at 37 °C in a humidified atmosphere with 5% CO_2_, the medium was replaced every 2–3 days, and subculturing was performed at 70–80% confluence using 0.25% trypsin-EDTA (Thermo Fisher Scientific or Euroclone). Cell lines were routinely tested to ensure absence of mycoplasma contamination.

### Viability Assay

For viability assays, PDAC cell lines were seeded in 96-well plates at a density of 5,000 cells/well, while fibroblasts and HPDE cells were seeded at 8,000 cells/well. After overnight attachment, cells were treated with the test compounds at final concentrations of 1, 10, 25, and 50 μM. Vehicle controls received an equivalent volume of DMSO, used as the compound solvent. After 48 h of treatment, cell viability was assessed using the MTT assay (3-(4,5-dimethylthiazol-2-yl)-2,5-diphenyltetrazolium bromide; Invitrogen, Cat. No. M6494) at a final concentration of 5.5 mg/mL. MTT reagent was added to the culture medium and incubated for 3 h at 37 °C. The medium was then aspirated, and the resulting formazan crystals were solubilized by adding 25 μL RPMI and 50 μL DMSO (Sigma-Aldrich, Cat. No. D2650) per well. Plates were incubated for an additional 40 min at 37 °C, and absorbance was measured at 540 nm using an Infinite M Nano+ microplate reader (Tecan, Cat. No. 30190087).

Absorbance values were normalized to the DMSO control, and viability was expressed as the percentage of viable cells relative to untreated controls. Data represent the mean ± standard deviation (SD) of technical triplicates. Compound concentrations were log_10_-transformed prior to curve fitting, and IC_50_ values were calculated using nonlinear regression with bottom and top constraints set to 0 and 100, respectively.

For viability assessment of non-PDAC cell lines, A549 (2,500 cells/well), ACHN (5000 cells/well), U251 (2500 cells/well), SH-SY5Y (7000 cells/well), BJ (1500 cells/well), and WI-38 (2500 cells/well) were seeded in 96-well plates. After overnight attachment, cells were treated with compound **6** and incubated for 48 h. Plates were then washed twice with PBS and fixed with 100 μL ice-cold methanol for 10 min on ice. After removal of methanol, 100 μL of crystal violet solution (1% w/v aqueous crystal violet; Sigma, V5265, supplemented with 20% ethanol) was added and incubated for 20 min at room temperature with gentle shaking. Wells were washed twice with water and air-dried. The retained dye was solubilized in 100 μL methanol with shaking for 30 min at room temperature. Absorbance was measured at 600 nm using a GloMax plate reader. Viability was calculated relative to vehicle-treated controls, and data represent mean ± SD of technical triplicates.

### Caco-2 Permeability Assay (Bidirectional)

For permeability studies, CACO-2 cells (NCCS) were seeded on cell inserts (cellQART) in a 24 wells plate at a density of 50,000 cells per well. The cells were maintained for 18–21 days in culture medium to enable differentiation, and the culture medium was changed every alternate day. HBSS buffered with 10 mM HEPES (pH 7.4) (HIMEDIA) was used on apical side as well as basolateral side. A stock solution of compounds was prepared in DMSO (Sigma) at a concentration of 50 mM followed by an intermediate stock solution of 500 μM in DMSO. This stock solution was spiked in the HBSS/HEPES buffer (pH 7.4) to get final test item concentration of 10 μM. The organic content of the final drug preparation was less than 1% v/v. On the day of the experiment, the cultured monolayer was washed twice with HBSS/HEPES buffer (0.25 mL and 0.6 mM was added to the apical and basolateral sides respectively, of the culture plate). Subsequently buffers from both the compartments were discarded. For apical to basal (A2B) experiment, aliquots of 0.25 mL donor solution (HBSS/HEPES buffer containing test compound) and 0.6 mL of HBSS/HEPES buffers were added to the apical and basolateral compartments, respectively. The plate was then kept in an incubator at 37 °C for 2 h. Control studies with propranolol (Sigma) (high permeable) and erythromycin (low permeable) in both the direction (AP > BL and BL > AP) were performed in separate wells in the same experiment. The bidirectional study was performed with compounds to ascertain the apical to basal permeability and basal to apical permeability. The study samples (collected from apical and basolateral compartments after 120 min incubation) were analyzed in LC-MS/MS instrument (SCIEX 4500 QTrap), subsequent to which the *P*_app_ of the compound(s) was calculated.

The apparent permeability (*P*_app_), in units of centimeters per second, was calculated for Caco-2 drug permeability assay using the following equation:

$$P_{\mathrm{app}}=\left(\mathrm{VA}/\mathrm{Area}\times \mathrm{Time}\right)\times \left({\left[\mathrm{drug}\right]}_{\mathrm{acceptor}}/{\left[\mathrm{drug}\right]}_{\mathrm{initial},\mathrm{donor}}\right)$$

*V*_A_ = Volume in the acceptor well

Area = Surface area of the membrane

Time = Total transport time in seconds

*P*_app_ = Apparent Permeability

Prior to initiating the experiment, the integrity of cellular monolayer was assessed by measuring the TEER value. Of note, the TEER value was measured in Ω·cm^2^ by volt ohm meter (EVOM^2^).(world precision instruments), measured TEER values were 168 Ω·cm^2^ (A → B) and 169 Ω·cm^2^ (B → A), confirming acceptable monolayer integrity.

At the end of permeability experiment, buffer was removed from both apical and basolateral compartments following which 250 μL of Lucifer Yellow (LY) (Sigma) at a concentration of 5 μM were added to each well in the filter plate and 600 μL HBSS buffer were assed to the basolateral compartments. The incubation was carried out for 1 h at 37 °C. Following the incubation, the samples were collected from the basolateral compartments and the LY the incubation, the samples were collected from the basolateral compartments, and the LY fluorescence was measured using an excitation wavelength of 485 nm and an emission wavelength of 530 nm. The percentage of LY rejection across the cell monolayer was calculated by measuring fluorescence in the receiver plate as compartment to equilibrium standard.

%LYPassage=[(RFU(test)−RFU(blank)/RFU(equilibrium)−RFU(blank)]×100

Where,

RFU (test) = Relative fluorescence units of the test sample

RFU (blank) = Relative fluorescence units of blank i.e., HBSS samples only without Lucifer yellow

RFU (equilibrium) = Relative fluorescence units of HBSS buffer containing 5 μM Lucifer yellow

These experiments were performed at o2h discovery Pvt Ltd., Ahmedabad, India.

### Washout Experiments

To assess the prolonged cellular effects of covalent inhibitors, washout experiments were performed using PANC-1 and MIA PaCa-2 cells. Cells were seeded in 48-well plates at a density of 5·10^3^ cells per well and allowed to adhere for 24 h. Subsequently, cells were treated with either DMSO (vehicle control) or 25 μM of **5r** or **6**. Treatments were conducted for defined time intervals of 15 min, 1, 2, 3, or 4 h, after which the treatment medium was aspirated, and cells were washed twice with PBS to remove residual compound. Fresh complete medium was then added to each well, and cells were incubated for an additional 72 h. At the end of the incubation period, cell viability was assessed using the MTT assay as previously described.

### Western Blot Analysis

For the first set of experiments, performed on PDAC cell lines, protein lysates (30 μg) were prepared in 4× sample buffer supplemented with β-mercaptoethanol, boiled at 98 °C for 2 min, and separated on 10–12% SDS-PAGE gels. Proteins were transferred to PVDF membranes (preactivated in methanol) using standard Tris-Glycine transfer buffer at 75 V for 90 min. Membranes were stained with Ponceau S, blocked in 5% nonfat milk in TBS-T for 1 h, and incubated overnight at 4 °C with primary antibodies against PFKFB3 (CST #13123, 1:1000), PFK (CST # #8164, 1:1000) and vinculin (CST #4650 and Sigma #V9131 CloneH, 1:1000). After washing, membranes were incubated for 1 h with HRP-conjugated secondary antibodies (CST #7074, 1:2000), washed, and developed using Pierce ECL substrate (Thermo Fisher Scientific #32106). Signals were imaged using a ChemiDoc imaging system (Bio-Rad) or an iBright1500 (Thermo Fisher Scientific).

In a second set of experiments, performed on other tumor cell lines (Figure S9a), protein lysates were prepared using 8 M urea lysis buffer supplemented with protease inhibitors, and 50 μg of protein per sample were loaded on Mini-PROTEAN TGX precast gels (Bio-Rad). Proteins were transferred using the Trans-Blot Turbo Transfer System (Bio-Rad). Membranes were incubated with primary antibodies against PFKFB3 (CST #13123, 1:1000 in TBS-T + 5% BSA, overnight at 4 °C) and vinculin (CST #4650, 1:10,000 in TBS-T + 5% BSA, 1 h at room temperature). After incubation with HRP-conjugated secondary antibodies (CST, 1:2000), blots were developed with Pierce ECL substrate and imaged using an iBright1500 system (Thermo Fisher Scientific). Uncropped Western blot data are provided in Figure S10.

### Zebrafish Experiments

*In-vivo* experiments were performed in the zebrafish (*Danio rerio*) model, which was maintained at the Interdepartmental Centre for Experimental Research (CIRSAL) of the University of Verona, in compliance with the European Directive 2010/63/EU on the protection of animals used for scientific purposes and in accordance to Italian law on animal experimentation (D.L. Four March 2014, n.26). Adult zebrafish were kept at 28 °C under a 14:10 h light:dark cycle with a 1 h phased sunrise and sunset transition. Embryos were obtained from the natural spawning of Nacre adults^58^ and maintained at 28.5 °C in fish water (0.114 mM NaH_2_PO_4_, 0.126 mM Na_2_HPO_4_, 0.3 g/L instant ocean) in Petri dishes.

For the dose-titration study, 3 days postfertilization (3 dpf) zebrafish larvae were randomly assigned to untreated (*n* = 17), vehicle-treated (0.1% DMSO, *n* = 29), or compound **6**-treated groups at 7, 10, 15, 25, and 50 μM (*n* = 30 per group). Larvae were exposed for 48 h, with compound refreshment at 24 h. Viability and locomotor activity were monitored throughout the treatment. Based on the findings, 7 μM was selected as the highest nontoxic and well-tolerated concentration for subsequent antitumor experiments. PANC-1 and MIA PaCa-2 cells (1 × 10^6^) were washed with 1× PBS and labeled with Vybrant Cell-Labeling Solution. Labeling was performed by adding 5 μL of dye to 1 mL PBS containing 2 mM EDTA, followed by incubation for 10 min at 37 °C in the dark, with gentle mixing at 5 min. Labeled cells were washed with serum-free RPMI 1640, centrifuged, and resuspended in 10 μL of serum-free RPMI containing 2 mM EDTA. Cell suspensions were kept on ice and protected from light until injection. At 2 dpf, embryos were manually dechorionated, anesthetized with tricaine (0.16 g/L; Sigma-Aldrich) and placed in a grooved agarose mold. Stained cells were loaded into a borosilicate glass capillary needle and microinjected into the zebrafish perivitelline space using a WPI PicoPump apparatus under a Leica M80 stereomicroscope. Injected embryos were then transferred to fish water and kept at 33 °C. At 1-day dpi, larvae were screened for injection success and morphological integrity; noninjected embryos and xenografts presenting cells in the yolk sac and/or edema were discarded. Correctly injected animals were randomly divided into two experimental groups: (i) untreated controls and larvae treated with 7 μM of compound **6**. After randomization, the animals were individually housed in 48-well plates with a final volume of 400 μL of medium per well. Treatments were refreshed daily throughout the experimental period. From 1 to 3 dpi (experimental end point), animals were imaged daily. Briefly, xenografts were anesthetized as described above, placed on a depression slide, and imaged by using a Leica MZ16F fluorescence microscope equipped with a DFC7000T camera. Sample sizes as follows: PANC-1 CTRL (T0 *n* = 11, T1 *n* = 10, T2 *n* = 8), PANC-1 compound **6** (T0 *n* = 12, T1 *n* = 11, T2 *n* = 9); MIA PaCa-2 CTRL (T0 *n* = 15, T1 *n* = 11, T2 *n* = 8), MIA PaCa-2 compound **6** (T0 *n* = 16, T1 *n* = 14, T2 *n* = 12). Data analysis was performed by measuring the integrated density of each embryo offline using the ImageJ software package Fiji (v. 1.54p).

### Liver Microsomal Stability

A 50 mM stock solution (in DMSO) was prepared for the compound. From the stock solution, a working solution of 0.05 mM was prepared by diluting the compound in buffer (note that this concentration of working solution was prepared considering a final concentration of 1 and 10 μM with 1% DMSO). A mixture containing 470 μL of buffer, 55 μL of NADPH (10 mM, SRL chemicals) and 11 μL of compounds (50 μM, 50% DMSO) was prewarmed at 37 °C in master mix tube for 15 min After incubation, 13.75 μL (20 mg/mL) of microsome were added in master mix tube. For the 0 min sample, 75 μL of sample was drawn from the master mix tube, and 150 μL of chilled acetonitrile (Finar) containing internal standard (IS, Carbamazepine) was added. The master mix tube was then incubated in a water bath at 37 °C for subsequent time points. Aliquots of samples were withdrawn at 0, 15, 30, 60, 90, and 120 min Add the time points that were used for the study. The reaction was stopped using chilled acetonitrile containing internal standard. The samples were centrifuged, and the supernatants collected and subsequently analyzed by LC-MS/MS. The percentage of remaining compound at each time point was calculated with respect to the 0 min sample. The data were then analyzed to determine the half-life and intrinsic clearance (CL_int_).

Control samples were run without NADPH, and blank samples were prepared using DMSO (without the test compound). These experiments were performed at o2h discovery Pvt Ltd., Ahmedabad, India.

### Metabolic Analysis

To evaluate residual phosphofructokinase (PFK) activity in cell lysates after treatment with the inhibitors, MIA PaCa-2 cells were seeded and allowed to adhere overnight. The following day, cells were treated for 2 h under three different conditions: DMSO (control), 25 μM compound **6**, or 25 μM compound **5r**. At the end of the incubation, cells were washed twice with PBS, and the resulting cell pellets were immediately flash-frozen. Finally, the cell pellets were flash-frozen. The cell paste was thawed and resuspended in 100 μL of lysis buffer (200 mM NaCl, 1 mM EDTA, 20 mM CHAPS, 10% sucrose, pH 7.0), then subjected to three freeze–thaw cycles. The resulting cell lysate was centrifuged at 16,000*g* for 45 min. Total protein concentration in the supernatant was determined by Bradford assay. PFK activity was measured using the coupled assay described above. The assay was performed at 37 °C in a microplate by adding either 20 μL of cell lysate or 20 μL of lysis buffer as a control. Readings at 340 nm were collected over time with a HaloLED 96-well microplate reader (Control Tecnica, Padova, Italy). The initial velocities (*V*_0_) were obtained by linear fitting of the time points and normalized to the protein content of each sample.

Metabolic profiling was performed using the Seahorse XFe24 Analyzer (Agilent Technologies). One day prior to the assays, sensor cartridges were hydrated overnight at 37 °C in a non-CO_2_ incubator. XF24 microplates were precoated with poly-d-lysine (100 μg/mL; Thermo Fisher Scientific, A3890401) and seeded with 20,000 cells per well (PANC-1 or MIA-PaCa2). Cells were allowed to adhere overnight.

For the ATP Rate Assay, MIA-PaCa2 cells were treated with 25 μM compound **6** for 1 or 2 h. Cells were then incubated in Seahorse XF RPMI supplemented with 10 mM glucose, 2 mM glutamine, and 1 mM sodium pyruvate for 1 h prior to measurement. Oligomycin (1.5 μM) and rotenone/antimycin A (0.5 μM) were sequentially injected, and all conditions were run in technical triplicate.

For the Glycolysis Stress Test, PANC-1 and MIA-PaCa2 cells were treated with compound **6** for 2 or 6 h. After treatment, culture medium was replaced with Seahorse XF RPMI supplemented with 2 mM glutamine and incubated for 1 h at 37 °C in a non-CO_2_ incubator. Glucose (10 mM), oligomycin (1 μM), and 2-deoxyglucose (50 mM) were sequentially injected, with all conditions performed in biological triplicate.

At the end of each assay, medium was discarded and plates stored at −80 °C overnight. Total DNA was quantified the following day using the CyQUANT Cell Proliferation Assay (Thermo Fisher Scientific, C7026) for normalization. Briefly, 200 μL of CyQUANT working solution (dye 1:400, lysis buffer 1:20 in sterile water) was added per well. After lysis, samples were transferred to a 96-well half a rea plate (Greiner, 675096), and fluorescence measured at 480/520 nm using a Tecan Infinite M Nano Plus. DNA content (μg) was determined from a standard curve and used to normalize metabolic rates in Wave software (Agilent Technologies).

### Drug Combination Studies

Drug combination experiments were conducted in PANC-1 and MIA PaCa-2 cells seeded in 96-well plates at a density of 5·10^3^ cells/well. After 24 h, cells were treated with compound **6** in combination with either gemcitabine or a FOLFIRINOX-like combination consisting of 5-fluorouracil (5-FU), irinotecan, and oxaliplatin, prepared at a clinically relevant molar ratio. Treatments were administered at a fixed molar ratio of [**6**]/[partner drug] = 1:10. Cell viability was assessed after 48 h using the MTT assay, as previously described.

The combination index (CI) was calculated using the Chou–Talalay method via Compusyn software (Biosoft, Cambridge, UK), following the approach described in Fiorini et al. CI values were interpreted as follows: CI < 0.3 (strong synergism), 0.3 < CI < 0.7 (synergism), 0.7 < CI < 1.0 (moderate synergism), CI = 1.0 (additive effect), and CI > 1.0 (antagonism). CI-effect curves were generated by plotting CI values against the fractional effect (Fa), representing the fraction of cells affected by the drug combination.

Isobologram plots were constructed using IC_25_, IC_50_, and IC_75_ values to evaluate drug interactions. The dose-reduction index at 50% effect (DRI_50_) was also calculated to determine the fold reduction in drug dose required to achieve 50% inhibition in combination relative to each agent alone. All analyses yielded linear correlation coefficients (*r*) > 0.90.

### Statistical Analysis

GraphPad Prism 10.3.1 was used for the statistical analysis. Statistical significance is described in the figure legends as * *p* < 0.05, ** *p* < 0.01, *** *p* < 0.001, **** *p* < 0.0001.

### Ethics Statement

All animal experiments were approved by Animal Welfare Organization (OPBA) and conducted according to the guidelines of Federation of European Laboratory Animal Science Association (FELASA).

## Supplementary Material





## Notes

Computational evaluation of ligand-PFKFB3 interaction (Figure S1); reactivity with model thiol βME (Figure S2); expression and characterization of PFKFB3 and its C154S variant (Figure S3); characterization of inhibitors of PFKFB3 and C154S PFKFB3 (Figure S4); evaluation of irreversible inhibition (Figure S5); MS/MS spectrum of peptide AFFIESVCDDPTVVASNIMEVK from PFKFB3 not treated with **6** (Figure S6); TREEspot data visualization of Eurofins DiscoverX of compound **6** (Figure S7); bioinformatic analyses of PFKFB3 expression and its prognostic relevance in PDAC (Figure S8); broad applicability of compound **6** across human cancer (Figure S9); full, uncropped Western Blots (Figure S10); PFKFB3 activity in the presence of compound **6** and excess MIA PaCa-2 protein extract (Figure S11); measured *m*/*z* values of **5–7** and their βME adducts (Table S1); kinetic parameters of PFKFB3 and its C154S variant (Table S2); summary of MaxQuant-identified peptides and associated spectral and confidence metrics (Table S3); kinase interactions (KINOMEscan Data, Table S4); Caco-2 permeability assay (Bidirectional, Table S5); dose–response and viability of zebrafish larvae exposed to compound **6** (Table S6); stability of compound **6** in human, rat, mouse and dog liver microsomes (Tables S7–S10); ^1^H NMR and ^13^C NMR spectra; and HPLC chromatograms for compounds **5–7** and **5r** (PDF)
Molecular formula strings (CSV)

## Abbreviations Used

βME: β-mercaptoethanol
CI: combination index
CTRL: control
DIPEA: *N*,*N*-diisopropylethylamine
F2,6BP: fructose-2,6-bisphosphate
F6P: fructose-6-phosphate
Fa: fraction affected
FOI: FOLFIRINOX-like chemotherapy cocktail excluding folinic acid
HBTU: *N,N,N′,N′*-Tetramethyl-*O-*(1*H*-benzotriazol-1-yl)­uronium hexafluorophosphate
HPDE: Human Pancreatic Duct Epithelial cells
IC_50_: half-maximal inhibitory concentration
*k*_cat_: turnover number
LDH: lactate dehydrogenase
NHDF: normal human dermal fibroblasts
OXPHOS: oxidative phosphorylation
PDAC: pancreatic ductal adenocarcinoma
PDF: probability distribution functions
PEP: phosphoenolpyruvate
PFK-1: phosphofructokinase-1
PFKFB: 6-phosphofructo-2-kinase/fructose-2,6-bisphosphatase
PK: pyruvate kinase
scRNA-seq: single-cell RNA sequencing
SEC: size-exclusion chromatography
TBAHS: tetrabutylammonium hydrogen sulfate
