# Supplementary material for: First-in-Class Covalent Inhibitors of PFKFB3: Discovery and Characterization in PDAC Models
Source: J Med Chem. 2026 May 26;69(11):13248–71. doi: 10.1021/acs.jmedchem.6c00235 (PMC13266998; doi:10.1021/acs.jmedchem.6c00235)
Supplement: Supplementary file 1 [file jm6c00235_si_001.pdf]

SUPPORTING INFORMATION

First-in-Class Covalent Inhibitors of PFKFB3: Discovery and Characterization in PDAC Models

Alessandra Fiore[1,10], Antonio Scarano[2,10], Giulia Antonini[3], Alexandra Ioana Corfù[3], Lea Sicuro[4,5], Serena Faggiano[2,6], Adriana Celesia[1,3], Chiara Tesoriero[7], Raffaella Pacchiana[1], Andrea Vettori[7], Liaisan Arslanbaeva[8], Saverio Minucci[8,9], Isabella Pallavicini[8], Luca Mollica[4], Lucia Tamborini[3], Massimo Donadelli[1], Paola Conti[3], Stefano Bruno[2,*], Chiara Borsari[3,*]

[1]Department of Neurosciences, Biomedicine and Movement Sciences, Section of Biochemistry, University of Verona, 37134 Verona, Italy

[2]Department of Food and Drug, University of Parma, Parco Area delle Scienze 23/a, 43124, Parma, Italy

[3]Department of Pharmaceutical Sciences, University of Milan, Via Mangiagalli 25, 20133 Milan, Italy

[4]Department of Medical Biotechnologies and Translational Medicine c/o L.I.T.A / University of Milan, Via F.lli Cervi 93, 20090 Segrate (MI), Italy

[5]Fondazione IRCCS Ca'Granda Ospedale Maggiore Policlinico, Angelo Bianchi Bonomi Hemophilia and Thrombosis Center, Milan, Italy

[6]Institute of Biophysics, National Research Council (CNR), Via G. Moruzzi 1, 56124 Pisa, Italy

[7]Department of Biotechnology, University of Verona, 37134 Verona, Italy

[8]Department of Experimental Oncology, IEO European Institute of Oncology IRCSS, 20139 Milan, Italy

[9]Department of Oncology and Hematology-Oncology, University of Milan, 20122 Milan, Italy

[10]These authors contributed equally: Alessandra Fiore, Antonio Scarano

*Correspondence to: chiara.borsari@unimi.it, Dept. of Pharmaceutical Sciences, University of Milan; Via Mangiagalli 25, 20133 Milan, Italy. Tel. +39 02503 19309. stefano.bruno@unipr.it, Dept. of Food and Drug, Parco Area delle Scienze 23\a, Parma, Italy. Tel. +39 0521906613

Table of Contents

Figure S1. Computational Evaluation of Ligand-PFKFB3 Interaction.. S4

Figure S2. Reactivity with Model Thiol βME. S5

Figure S3. Expression and Characterization of PFKFB3 and its C154S Variant. S6

Figure S4. Characterization of Inhibitors of PFKFB3 and C154S PFKFB3...................................... S7

Figure S5. Evaluation of Irreversible Inhibition. S8

Figure S6. MS/MS spectrum of peptide AFFIESVCDDPTVVASNIMEVK from PFKFB3 not treated with **6**. S9

Figure S7. TREEspot Data Visualization of Eurofins DiscoverX of Compound **6**. S10

Figure S8. Bioinformatic Analyses of PFKFB3 Expression and its Prognostic Relevance in PDAC. S11

Figure S9. Broad Applicability of Compound **6** across Human Cancer. S12

Figure S10. Full, Uncropped Western Blots. S13

Figure S11. PFKFB3 Activity in the Presence of Compound **6** and Excess MiaPaCa-2 Protein Extract S14

Table S1. Measured m/z Values of Compounds **5-7** and their βME Adducts.................................. S15

Table S2. Kinetic Parameters of PFKFB3 and its C154S Variant. S15

Table S3. Summary of MaxQuant-Identified Peptides and Associated Spectral and Confidence Metrics. S15

Table S4. Kinase Interactions (KINOMEscan Data). S16

Table S5. Caco-2 Permeability Assay (Bidirectional). S19

Table S6. Dose-Response and Viability of Zebrafish Larvae Exposed to Compound **6**.................... S20

Table S7. Stability of Compound **6** in Human Liver Microsomes.. S21

Table S8. Stability of Compound **6** in Rat Liver Microsomes.. S21

Table S9. Stability of Compound **6** in Mouse Liver Microsomes.. S22

Table S10. Stability of Compound **6** in Dog Liver Microsomes.. S22

^{1}H and ^{13}C NMR Spectra.. S23

HPLC Chromatograms for Compounds 5-7 and 5r.. S34

Figure S1. Computational Evaluation of Ligand-PFKFB3 Interaction.

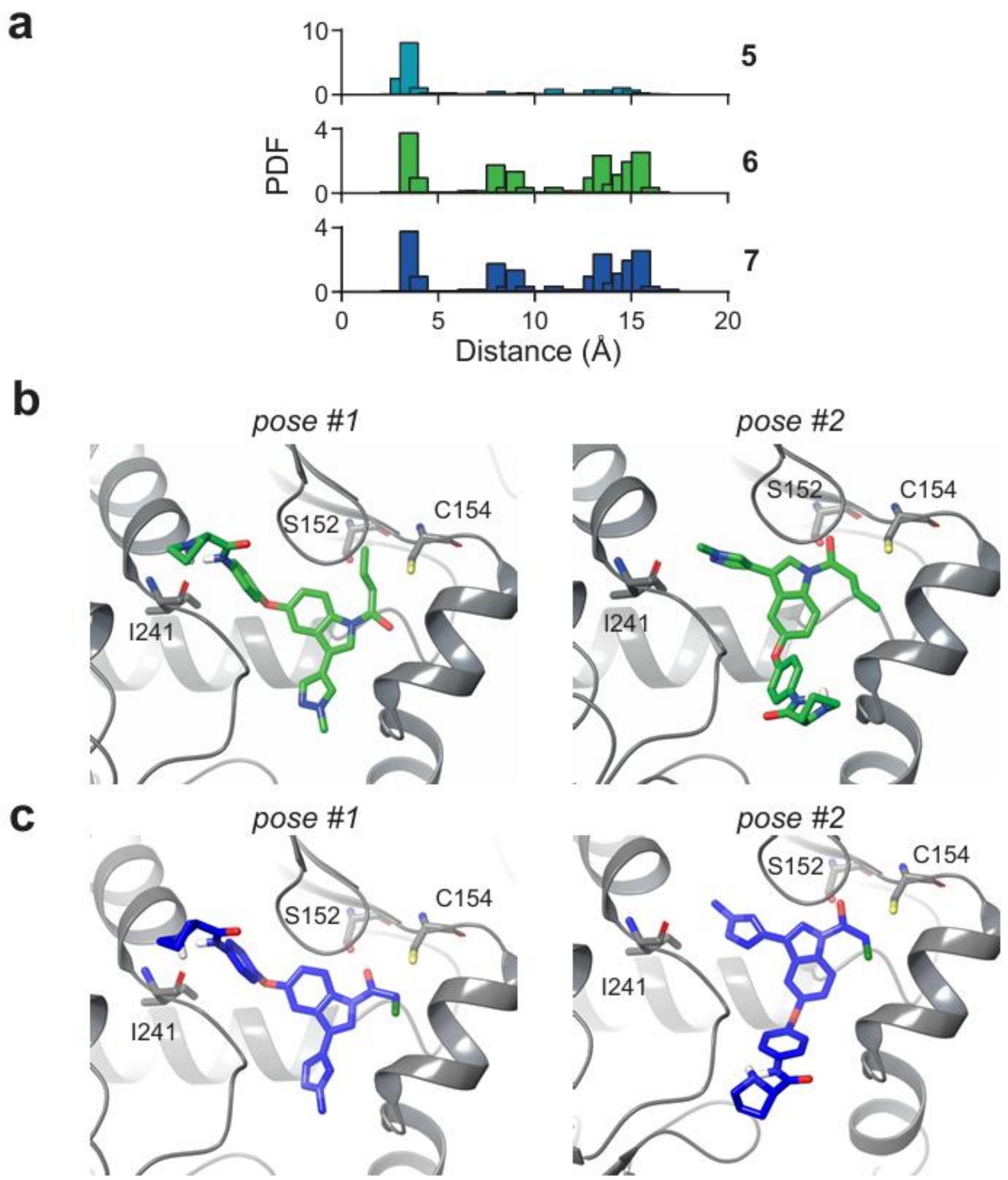

Figure S1. (**a**) Statics of the distance between the Cys154 Sγ atom and the ligand carbon atom which act as donor and acceptor during the covalent bond formation, calculated during docking calculations and expressed in angstrom (Å) for **5**, **6**, and **7**. Probability distribution functions (PDF) are expressed in arbitrary units. (**b-c**) Representative structures of *pose #1* and *pose #2* for **6** (b) and for **7** (c).

Figure S2. Reactivity with Model Thiol βME.

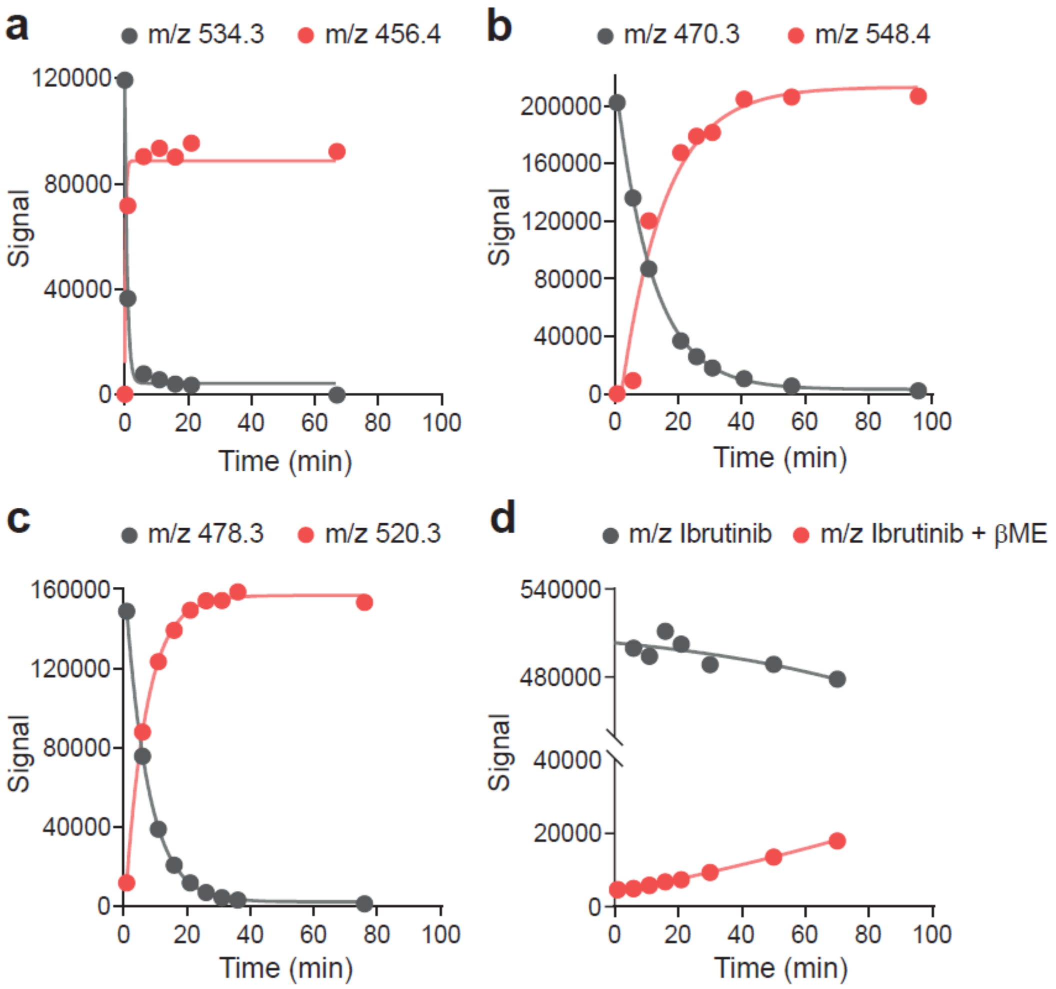

Figure S2. Reaction of βME (60 mM) with **5** (a), **6** (b), **7** (c) and ibrutinib (d), each at 100 µM, incubated at 25 °C. The m/z values corresponding to the adduct between the compounds and βME (red circles) and the compounds alone (black circles) were monitored over time by ESI-MS. The intensities were normalized to the end-point value and fitted using a shifted exponential function (solid lines).

Figure S3. Expression and Characterization of PFKFB3 and its C154S Variant.

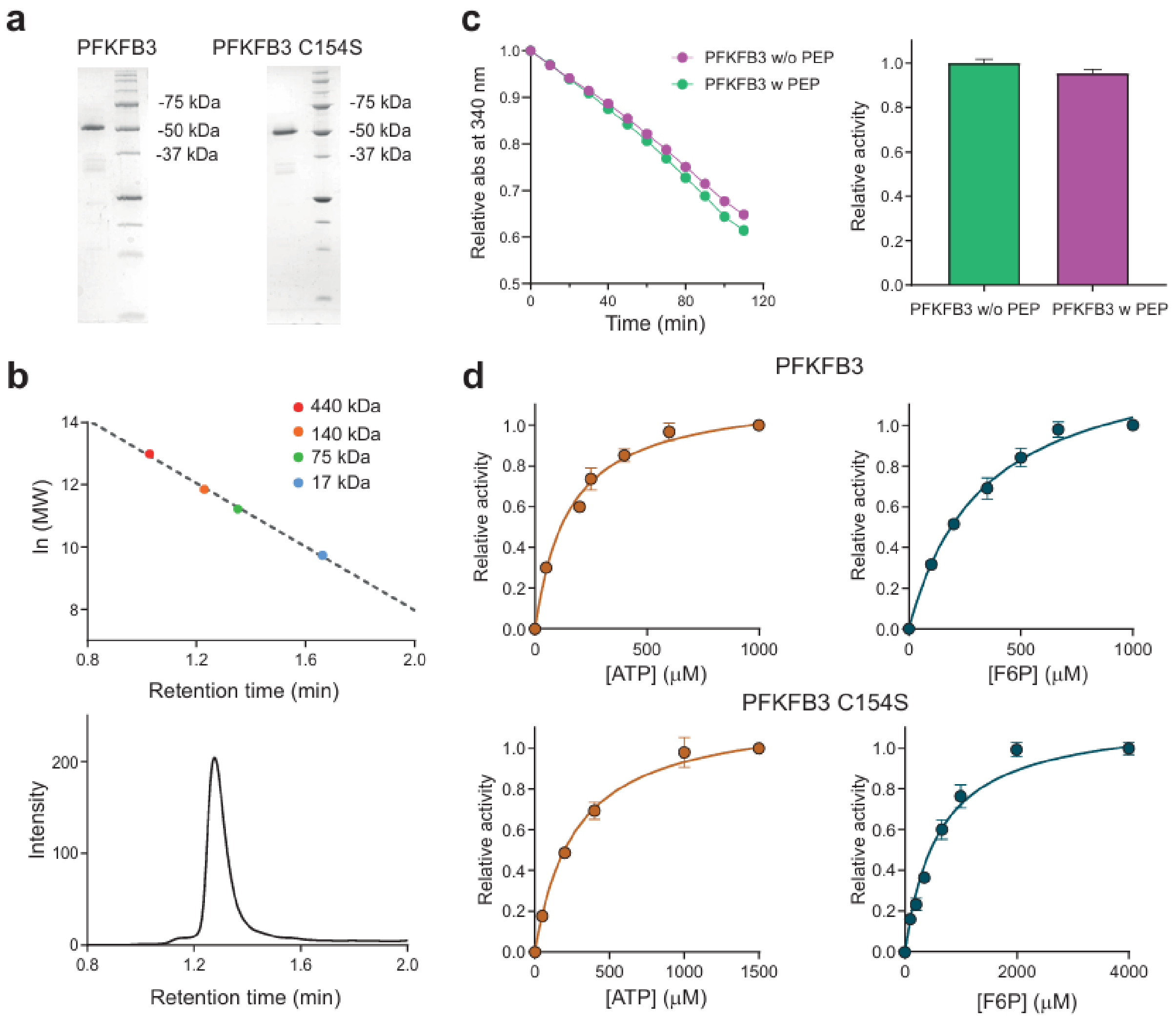

Figure S3. (**a**) SDS–PAGE (12%) of purified PFKFB3 (left) and C154S PFKFB3 (right). Densitometry (ChemiDoc, Bio-Rad) showed purities of 97% and 87%. Typical yields were 2–4 mg/L of culture. (**b**) SEC analysis of purified PFKFB3 and calibration curve (upper panel) using apoferritin (440 kDa), aldolase (140 kDa), conalbumin (75 kDa), and myoglobin (17 kDa). PFKFB3 was loaded at 33 μM, yielding an apparent MW of 114600 Da. (**c**) Coupled enzyme assay (discontinuous) in the presence or absence of PEP (1 mM) at 37 °C in a buffer containing 10 mM sodium phosphate, 50 mM NaCl, and 10 mM $MgCl_2$, pH 7.5 (left). Normalized activities are shown on the right. (**d**) Michaelis–Menten kinetics for PFKFB3 and C154S PFKFB3 using F6P (blue; ATP fixed at 600 μM) or ATP (orange; F6P fixed at 1 mM). Activities were normalized to V_{max}, and data were fitted with the Michaelis–Menten equation. Assays were performed at 37 °C in 10 mM sodium phosphate, 50 mM NaCl, and 10 mM $MgCl_2$, pH 7.5. Data shown are mean ± SEM (n = 3; independent measurements). Error bars are not shown when smaller than symbols.

Figure S4. Characterization of Inhibitors of PFKFB3 and C154S PFKFB3.

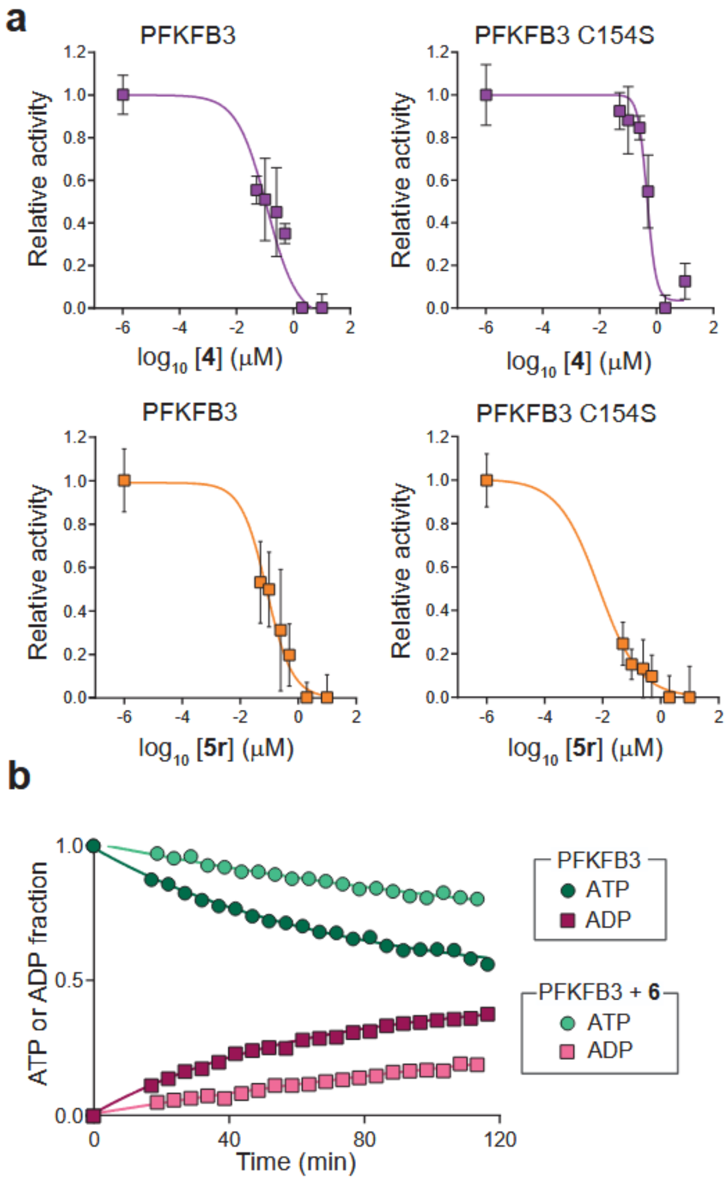

Figure S4. (**a**) Dependence of enzyme activity on the concentration of the reversible inhibitors **4** and **5r** at concentrations ranging from 0.05 to 10 μM. The enzymes were at 50 nM concentration. Data shown are mean ± SEM (n = 3; independent measurements). Error bars are not shown when smaller than symbols. (**b**) Kinetics of ATP consumption (circles) and ADP formation (squares) as monitored by integrating the peak related to the purine H8 proton of ^{1}H NMR spectra acquired over time in the absence (dark green and dark purple) and presence (light green and light purple) of **6**.

Figure S5. Evaluation of Irreversible Inhibition.

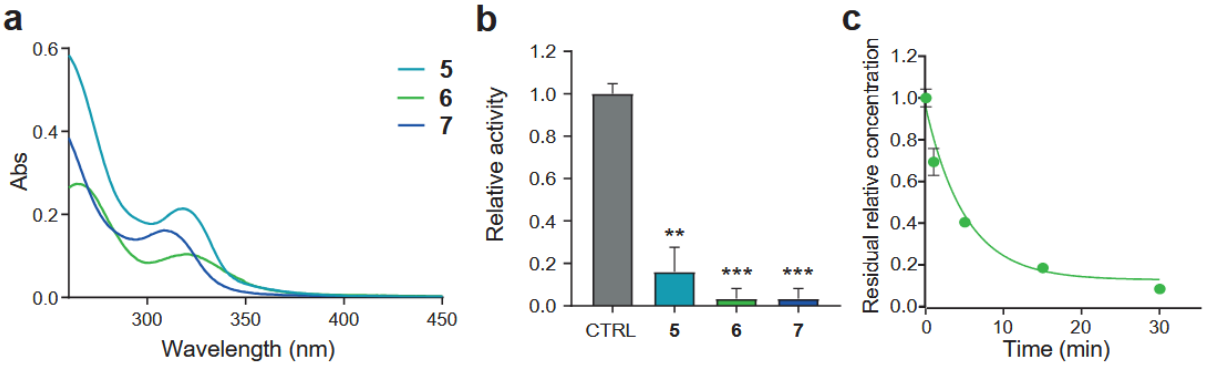

Figure S5. (**a**) Absorption spectra of compounds **5-7** in a buffer solution containing 50 mM HEPES, 200 mM NaCl, 0.2 mM EDTA, 10% (v/v) TFA. (**b**) Residual activity of PFKFB3 (2.5 µM) upon incubation at 25 °C with **5-7** at 10 µM concentration, followed by dialysis for 3 h against a solution containing 10 mM sodium phosphate, 50 mM NaCl, and 0.2% Tween 20, pH 7.5. Residual enzyme activity is expressed as fraction of the activity of the untreated control. (c) TFA-mediated co-precipitation assays of PFKFB3 (25 µM) incubated with **6** (16.5 µM) followed over time. Absorbance at 323 nm at different timepoints (1, 5, 15 and 30 minutes) was monitored until complete protein precipitation was achieved. The obtained points were fitted to an exponential decay. Data shown are mean ± SEM (n = 3; independent measurements). Error bars are not shown when smaller than symbols.

Figure S6. MS/MS spectrum of peptide AFFIESVCDDPTVVASNIMEVK from PFKFB3 not treated with **6**.

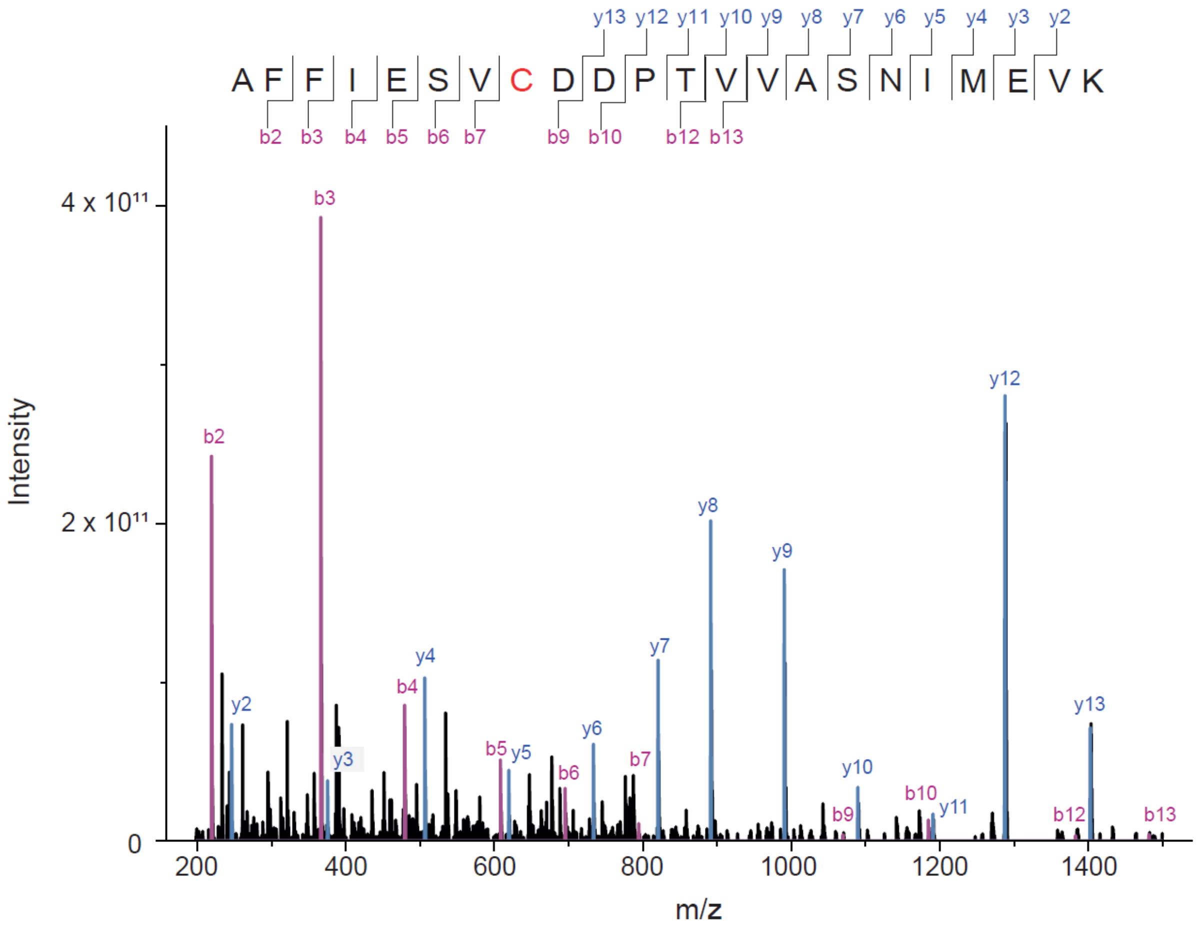

Figure S6. MS/MS spectrum of the peptide AFFIESVCDDPTVVASNIMEVK obtained by HCD fragmentation under the same experimental conditions described for PFKFB3 treated with **6**, in the absence of the compound. The peptide was identified with high confidence (score up to ~284, PEP in the 10^{-84} range), exhibiting sub-ppm mass accuracy. The fragmentation pattern yields b- and y-ion series consistent with cysteine residues being carbamidomethylated (+57.02 Da), with extensive sequence coverage supporting the assignment. Fragment annotation and scoring were performed using Skyline.

Figure S7. TREEspot Data Visualization of Eurofins DiscoverX of Compound **6**.

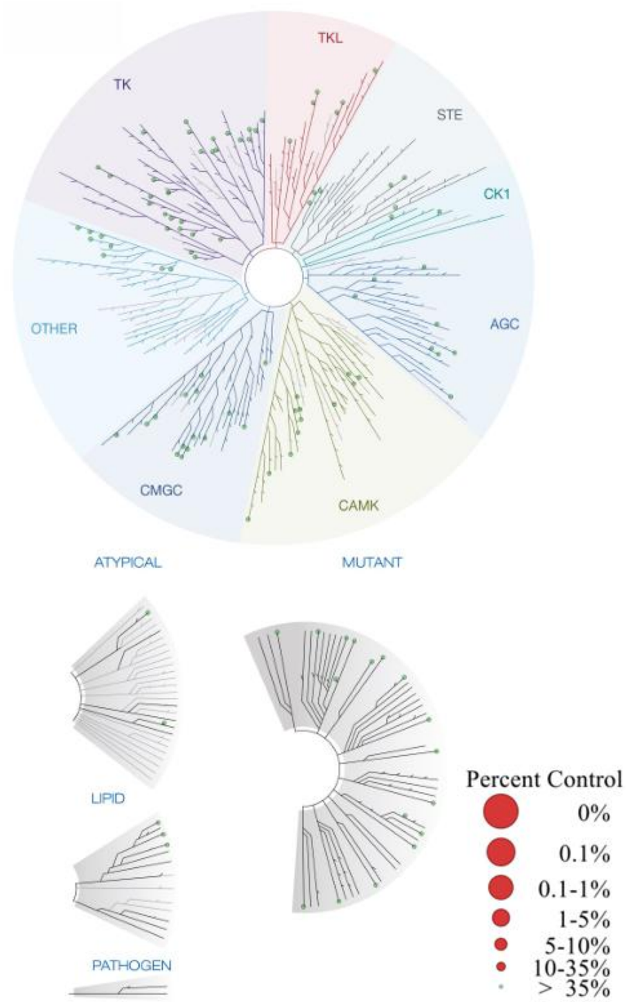

Figure S7. Kinases targeted by indicated compounds are marked with red circles, where increasing circle size indicates a higher affinity. Remaining unbound kinase is depicted as % of vehicle control at 10 μM of added compound. Kinases are organized according to phylogeny and kinase families, including atypical kinases, lipid kinases, mutated kinases and some pathogen-derived kinases. The raw data used for the TREEspot™ representation is shown in Table S4.

Figure S8. Bioinformatic Analyses of PFKFB3 Expression and its Prognostic Relevance in PDAC.

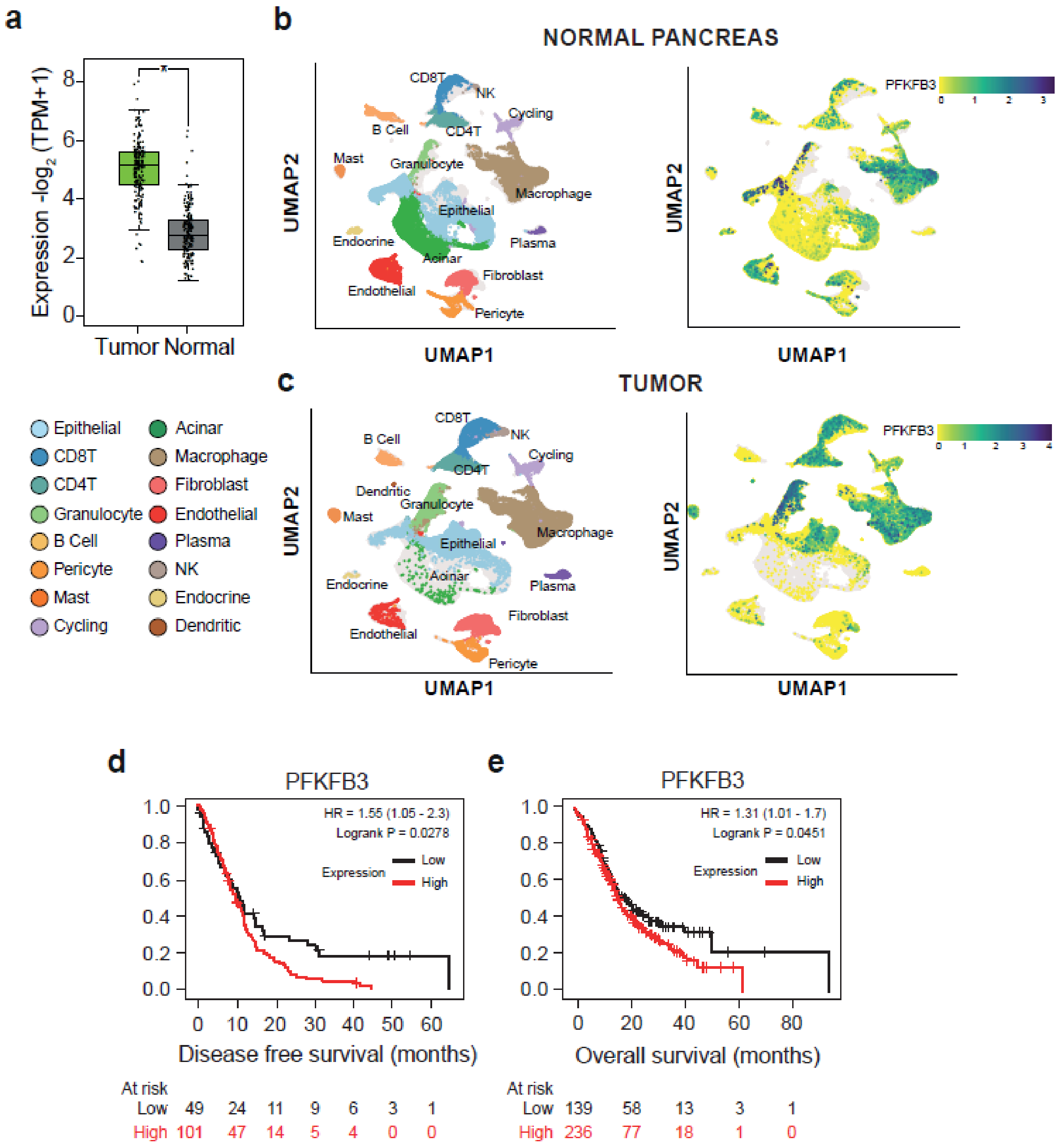

Figure S8. (**a**) Gene Expression Profiling Interactive Analysis (GEPIA) analysis of PFKFB3 expression levels in tumors isolated from PDAC patients compared with healthy pancreatic tissues (Tumor n = 179 and Normal n = 171; ± SD; significance was determined using one-way ANOVA; P ≤ 0.05). (**b,c**) Uniform manifold approximation and projection (UMAP) visualization of all identified cell types present in the pancreatic microenvironment subsets from the Pancreatic Tissue Single Cell Atlas, and UMAP visualizations showing PFKFB3 expression across the major cell populations subset by disease state (Normal pancreas in b and Tumor in c). (**d**) Kaplan–Meier (KM) plot of disease-free survival (log-rank test) for PDAC patients obtained from the KM-plotter database using default parameters, showing

that high PFKFB3 expression is associated with reduced disease-free survival. (**e**) Kaplan–Meier survival plot after stratification of PDAC patients by tumor grade (≥ G2) in the KM-plotter database, indicating that elevated PFKFB3 expression is a negative prognostic factor for overall survival in advanced pancreatic cancer.

Figure S9. Broad Applicability of Compound **6** across Human Cancer.

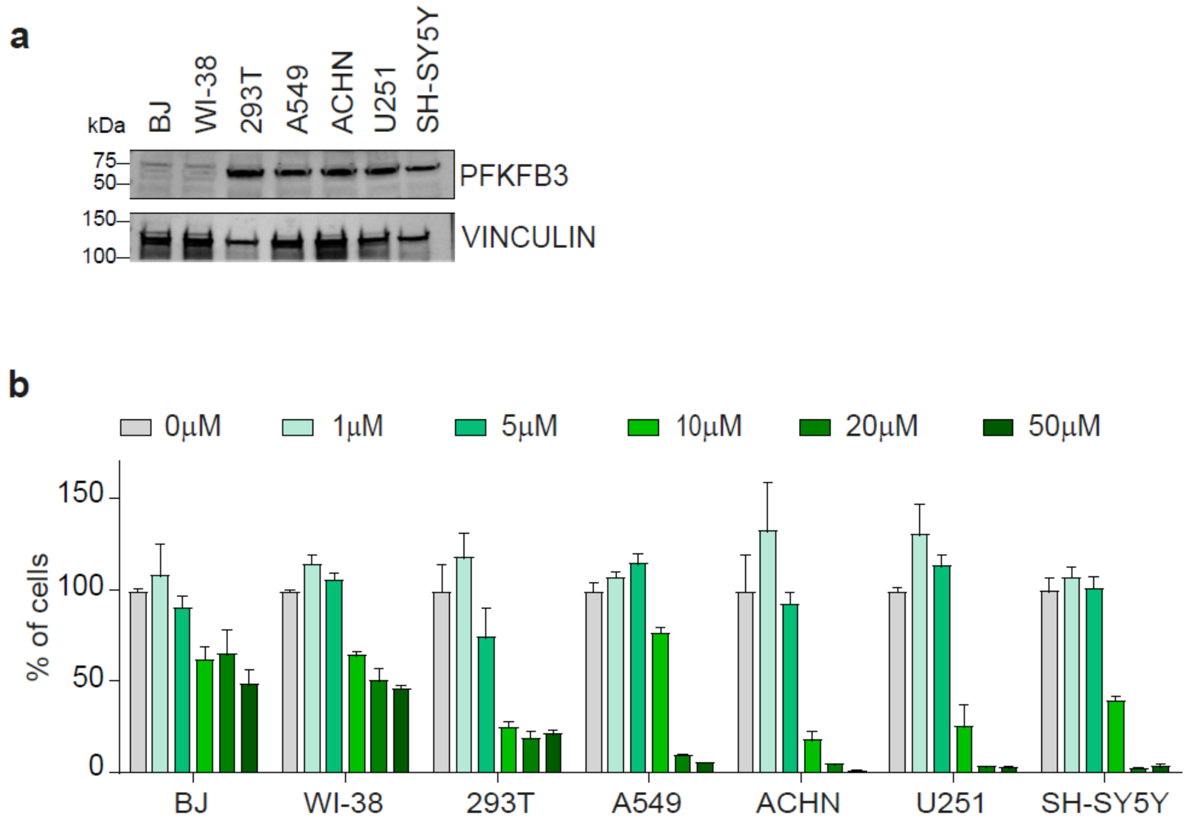

Figure S9. (**a**) Immunoblot analysis of PFKFB3 expression in BJ, WI-38, 293T, A549, ACHN, U251 and SH-SY5Y cell lines. Vinculin was used as a loading control. (**b**) Histogram showing cell viability in BJ, WI-38, 293T, A549, ACHN, U251 and SH-SY5Y cells after 48 h treatment with compound **6** at increasing concentrations (1, 5, 10, 20, 50 µM). Cell viability was measured by crystal violet assay and expressed as a percentage relative to the corresponding DMSO control for each cell line. Bars represent mean ± SD of three independent biological replicates. The Y-axis indicates cell viability (%).

Figure S10. Full, Uncropped Western Blots.

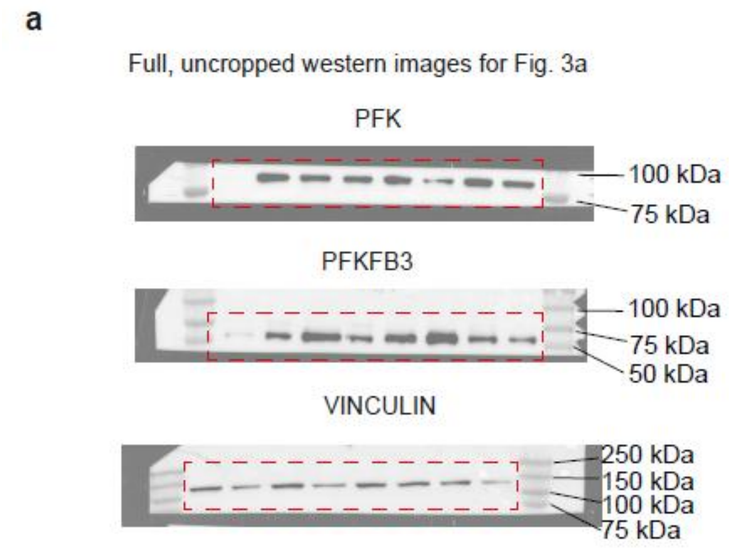

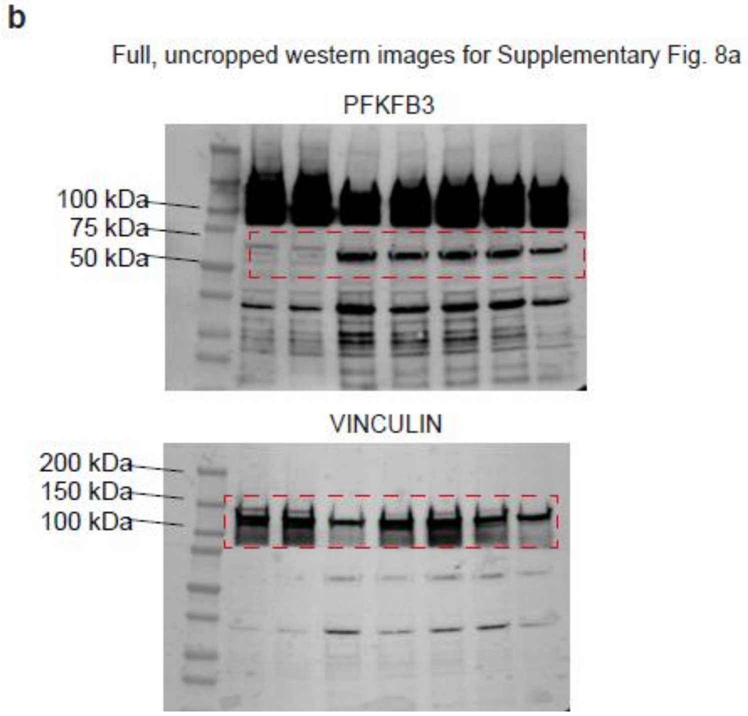

Figure S10. (**a**) Full, uncropped Western blot corresponding to Fig. 3a. (**b**) Full, uncropped Western blot corresponding to Figure S8a.

Figure S11. PFKFB3 Activity in the Presence of Compound **6** and Excess MiaPaCa-2 Protein Extract

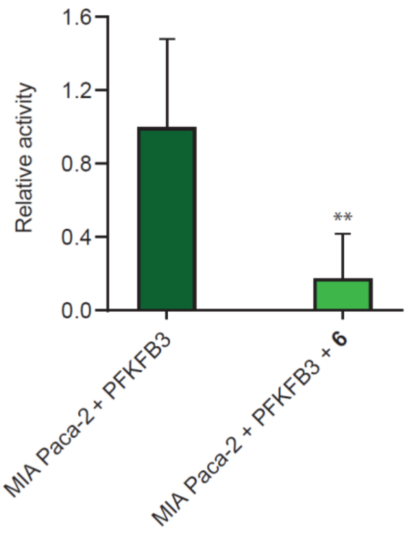

Figure S11. Activity of PFKFB3 (17 µM) following incubation with compound **6** (25 µM) for 30 min at 20 °C in buffer (20 mM sodium phosphate, 100 mM NaCl, 0.2% Tween 20, pH 7.5) in the presence of MIA PaCa-2 total protein extract (2.4 mg/mL, Bradford assay), corresponding to approximately a 10-fold excess of total protein relative to PFKFB3. The control corresponds to the reaction performed in the absence of compound **6**. For both control and treated samples, the measured velocities were corrected by subtracting the basal activity of the cell extract determined in the absence of exogenous PFKFB3. Bars represent the mean ± SD of five independent replicates. Statistical significance was assessed using a paired two-tailed t-test (**$p < 0.01$).

Table S1. Measured m/z Values of Compounds **5-7** and their βME Adducts.

Compound	m/z	Compound m/z + βME
**5**	456.4	534.3
**6**	470.3	548.4
**7**	478.3	520.3

Table S2. Kinetic Parameters of PFKFB3 and its C154S Variant.

	K_m^{ATP}	K_m^{F6P}	k_{cat}^{ATP}	k_{cat}^{F6P}	k_{cat}/K_m (ATP)	k_{cat}/K_m (F6P)
**PFKFB3**	151 ± 9 μM	331 ± 44 μM	0.1783 s^{-1}	0.1783 s^{-1}	1180 ± 67 $M^{-1}\cdot s^{-1}$	539 ± 71 $M^{-1}\cdot s^{-1}$
**PFKFB3 C154S**	272 ± 13 μM	643 ± 70 μM	0.3826 s^{-1}	0.3826 s^{-1}	1409 ± 67 $M^{-1}\cdot s^{-1}$	595 ± 65 $M^{-1}\cdot s^{-1}$

Table S3. Summary of MaxQuant-Identified Peptides and Associated Spectral and Confidence Metrics.

Peptide Sequence	# Spectra	Best Score	PEP
AFFIESVCDDPTVVASNIMEVK	6+	192.98	$2.77\cdot10^{-75}$
ISCYEASYQPLDPDKCDR	3	126.79	$4.72\cdot10^{-67}$
KQCALAALR	2	126.85	$3.91\cdot10^{-8}$
QCALAALR	1	84.20	$1.36\cdot10^{-6}$
CPLHTVLK	1	106.86	$5.67\cdot10^{-6}$
ISSPDYK	1	101.06	$3.46\cdot10^{-5}$

Table S4. Kinase Interactions (KINOMEscan Data).

DiscoveRx Gene Symbol	% remaining @ 10 µM for compound **6**
ABL1(E255K)-phosphorylated	100
ABL1(T315I)-phosphorylated	82
ABL1-nonphosphorylated	99
ABL1-phosphorylated	85
ACVR1B	91
ADCK3	84
AKT1	96
AKT2	100
ALK	76
AURKA	58
AURKB	92
AXL	87
BMPR2	87
BRAF	95
BRAF(V600E)	79
BTK	78
CDK11	93
CDK2	78
CDK3	99
CDK7	100
CDK9	100
CHEK1	74
CSF1R	35
CSNK1D	19
CSNK1G2	92
DCAMKL1	100
DYRK1B	100
EGFR	40
EGFR(L858R)	64
EPHA2	99
ERBB2	91
ERBB4	59
ERK1	79
FAK	94
FGFR2	95
FGFR3	95
FLT3	100
GSK3B	64
IGF1R	98

DiscoveRx Gene Symbol	% remaining @ 10 μM for compound **6**
IKK-alpha	82
IKK-beta	98
INSR	100
JAK2(JH1domain-catalytic)	91
JAK3(JH1domain-catalytic)	95
JNK1	82
JNK2	100
JNK3	95
KIT	100
KIT(D816V)	100
KIT(V559D,T670I)	100
LKB1	100
MAP3K4	100
MAPKAPK2	85
MARK3	66
MEK1	90
MEK2	100
MET	100
MKNK1	100
MKNK2	100
MLK1	100
p38-alpha	100
p38-beta	84
PAK1	88
PAK2	100
PAK4	100
PCTK1	94
PDGFRA	86
PDGFRB	84
PDPK1	83
PIK3C2B	98
PIK3CA	100
PIK3CG	91
PIM1	75
PIM2	100
PIM3	95
PKAC-alpha	94
PLK1	99
PLK3	99
PLK4	93

DiscoveRx Gene Symbol	% remaining @ 10 µM for compound **6**
PRKCE	67
RAF1	87
RET	100
RIOK2	100
ROCK2	99
RSK2(Kin.Dom.1-N-terminal)	100
SNARK	89
SRC	100
SRPK3	92
TGFBR1	76
TIE2	85
TRKA	91
TSSK1B	94
TYK2(JH1domain-catalytic)	90
ULK2	98
VEGFR2	90
YANK3	81
ZAP70	89

Table S5. Caco-2 Permeability Assay (Bidirectional).

Comp.	Average Values					
	Papp (10^{-6} cm/sec)	Papp (10^{-6} cm/sec)	Efflux Ratio	A to B % Recovery	B to A % Recovery	Classification
	Apical to Basal	Basal to Apical				
Propranolol	53.31	26.78	0.50	98.90	82.32	High
Erythromycin	0.30	8.36	27.57	82.63	93.53	Low
**6**	1.57	11.91	7.57	25.98	57.44	Medium

Result of permeability Papp (10^{-6}cm/s)	Range
<1.5	Low permeable
1.5 to 10	Medium permeable
>10	High permeable

Table S6. Dose-Response and Viability of Zebrafish Larvae Exposed to Compound **6**.

Compound 6 (µM)	Number of Larvae	Mortality	Locomotor alterations			Outcome
			Responsive	Low-responsive	No-responsive	
**0 (untreated ctrl)**	17	0 % (0/30)	100 % (17/17)	0 % (0/17)	0 % (0/17)	No toxicity
**0 (dmso ctrl)**	29	0 % (0/29)	100 % (29/29)	0 % (0/29)	0 % (0/29)	No toxicity
**7**	30	0 % (0/30)	100 % (30/30)	0 % (0/30)	0 % (0/30)	No toxicity
**10**	30	6.7 % (2/30)	60 % (18/30)	23.3 % (7/30)	10 % (3/30)	High mortality and toxicity
**15**	30	66.7 % (20/30)	0 % (0/30)	0 % (0/30)	33.3 % (10/30)	Severe mortality and toxicity
**25**	30	100 % (30/30)	---	---	---	Lethal
**50**	30	100 % (30/30)	---	---	---	Lethal

Table S7. Stability of Compound **6** in Human Liver Microsomes.

**Compound Name**	**Half life (min)**	**% Rem @ 30 min**	**% Rem @ 60 min**	**% Rem @ 120 min**	**% Rem @ 120 min W/O Cofactor**	**CL int protein (μL/min/mg protein)**	**CL invivo (mL/min/kg BW)-Well stirred model**	**%QH (Well stirred model)**	**Classification**
Verapamil	17,79	29,88	13,99	-	98,51	77,93	15,86	75,53	High
**6** (1μM)	62,68	67,29	49,84	27,21	32,11	22,24	9,82	46,77	Medium
**6** (10μM)	68,18	58,25	49,36	36,25	26,16	21,10	9,47	45,12	Medium

Classification criteria	%QH
Low Clearance	<30
Medium Clearance	30-70
High Clearance	>70

Table S8. Stability of Compound **6** in Rat Liver Microsomes.

**Compound Name**	**Half life (min)**	**% Rem @ 30 min**	**% Rem @ 60 min**	**% Rem @ 120 min**	**% Rem @ 120 min W/O Cofactor**	**CL int protein (μL/min/mg protein)**	**CL invivo (mL/min/kg BW)-Well stirred model**	**%QH (Well stirred model)**	**Classification**
Verapamil	15,35	26,14	10,28	-	108,18	90,44	48,94	69,91	High
**6** (1μM)	109,43	109,45	75,33	51,60	60,13	12,67	17,20	24,57	Low
**6** (10μM)	>120	87,35	77,91	61,13	56,40	9,08	13,24	18,92	Low

Classification criteria	%QH
Low Clearance	<30
Medium Clearance	30-70
High Clearance	>70

Table S9. Stability of Compound **6** in Mouse Liver Microsomes.

Compound Name	Half life (min)	% Rem @ 30 min	% Rem @ 60 min	% Rem @ 120 min	% Rem @ 120 min W/O Cofactor	CL int protein (µL/min/mg protein)	CL invivo (mL/min/kg BW)-Well stirred model	%QH (Well stirred model)	Classification
Verapamil	7,60	11,81	5,23	-	72,22	182,29	80,02	88,91	High
**6** (1µM)	15,53	26,20	14,21	8,24	6,30	89,28	71,74	79,71	High
**6** (10µM)	11,66	16,80	5,45	4,47	3,33	118,88	75,55	83,95	High

Classification criteria	%QH
Low Clearance	<30
Medium Clearance	30-70
High Clearance	>70

Table S10. Stability of Compound **6** in Dog Liver Microsomes.

Compound Name	Half life (min)	% Rem @ 30 min	% Rem @ 60 min	% Rem @ 120 min	% Rem @ 120 min W/O Cofactor	CL int protein (µL/min/mg protein)	CL invivo (mL/min/kg BW)-Well stirred model	%QH (Well stirred model)	Classification
Verapamil	18,32	31,87	13,55	-	112,79	75,67	26,63	85,90	High
**6** (1µM)	14,30	23,36	5,65	4,42	4,91	96,95	27,48	88,64	High
**6** (10µM)	12,24	18,28	4,38	1,71	5,73	113,61	27,94	90,12	High

Classification criteria	%QH
Low Clearance	<30
Medium Clearance	30-70
High Clearance	>70

^{1}H and ^{13}C NMR Spectra

5-(4-Nitrophenoxy)-1*H*-indole (**9**) – ^{1}H NMR

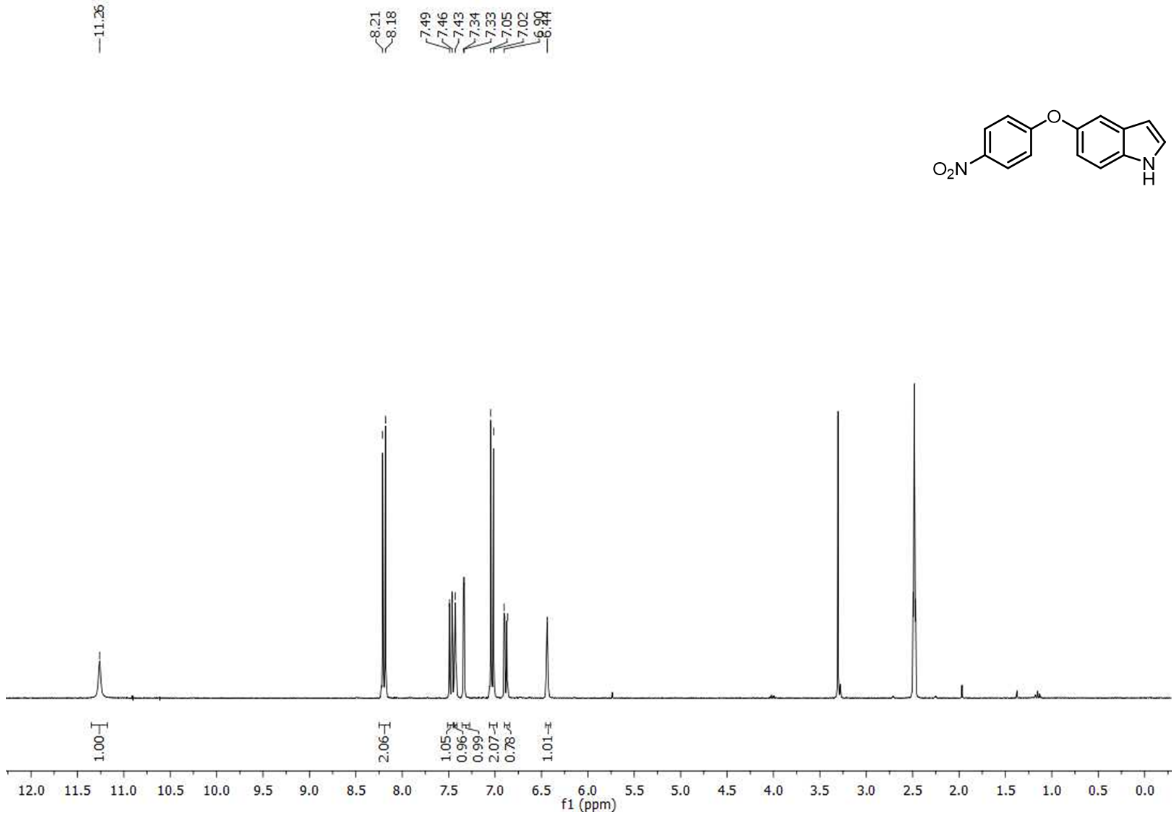

3-Bromo-5-(4-nitrophenoxy)-1*H*-indole (**10**) – ^{1}H NMR

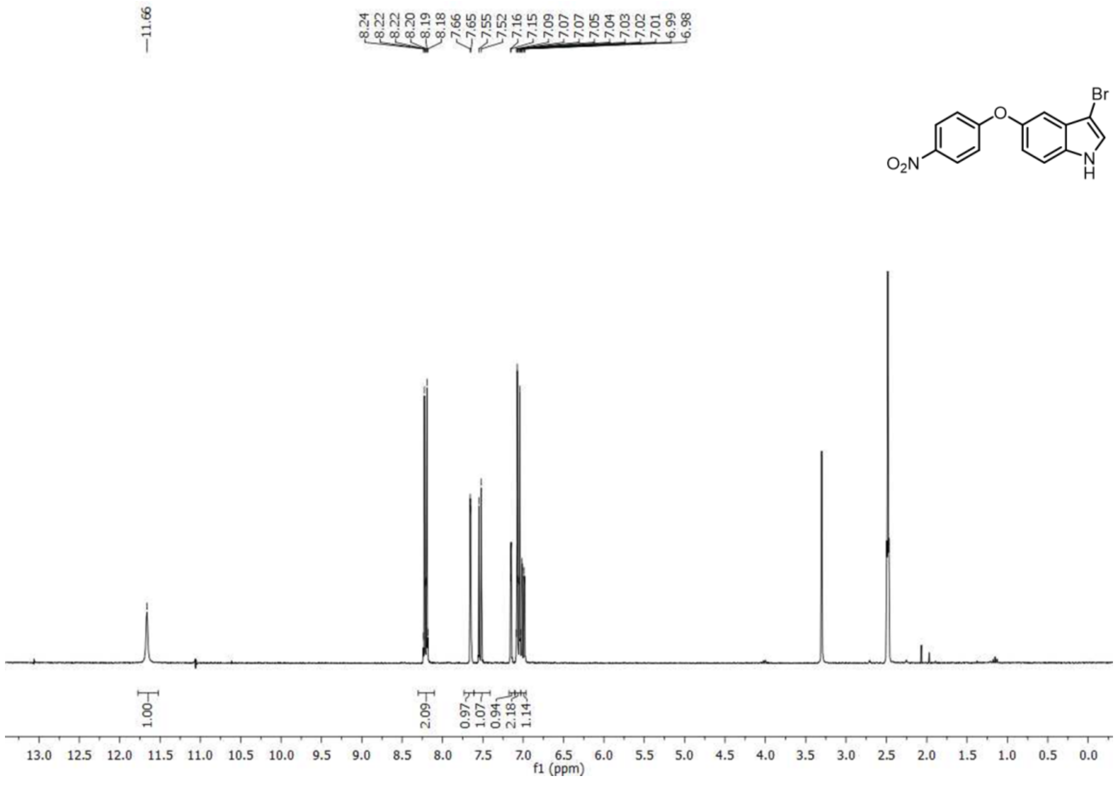

3-Bromo-5-(4-nitrophenoxy)-1*H*-indole (**10**) - ^{13}C NMR

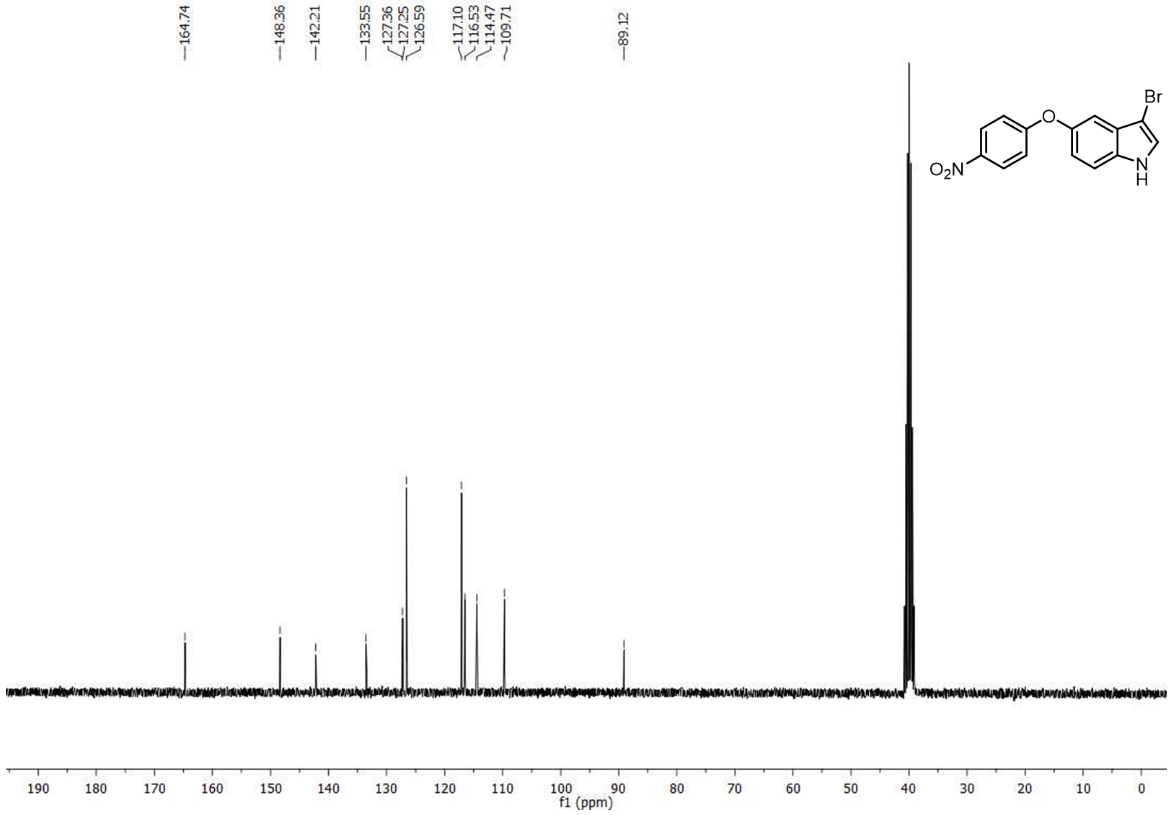

3-(1-Methyl-1*H*-pyrazol-4-yl)-5-(4-nitrophenoxy)-1*H*-indole (**11**) – ^{1}H NMR

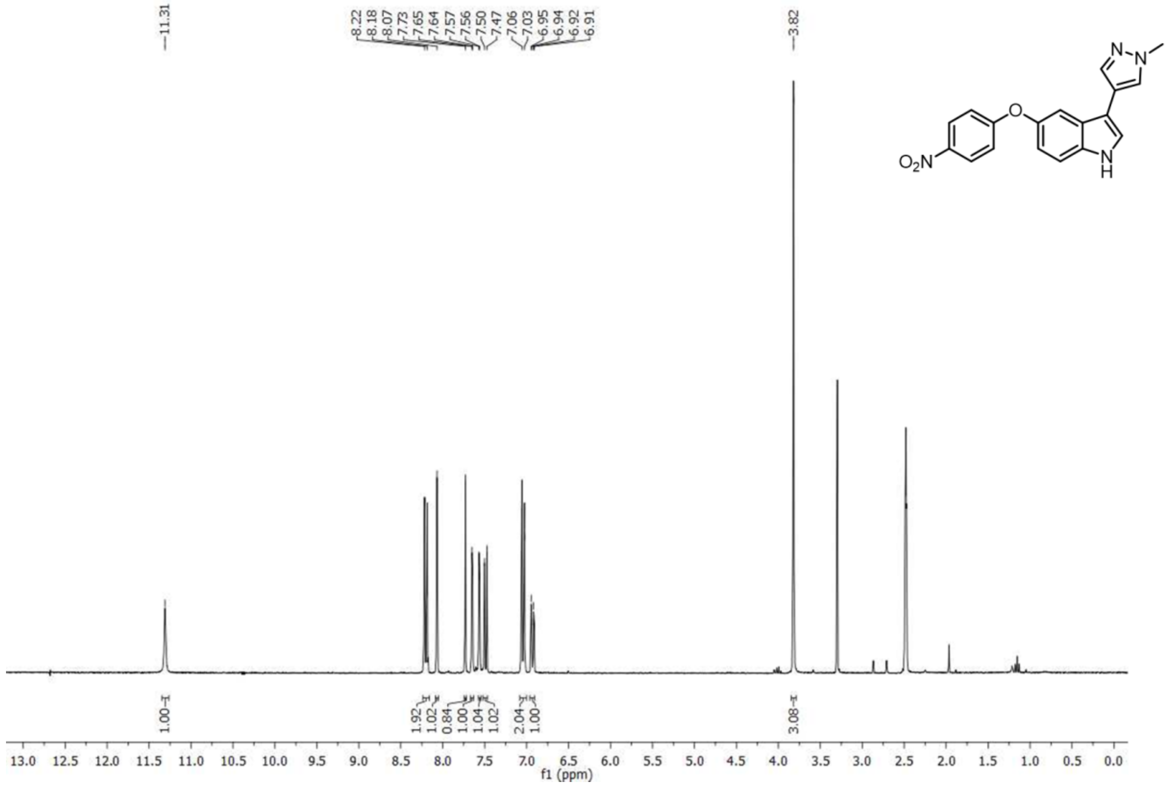

3-(1-Methyl-1*H*-pyrazol-4-yl)-5-(4-nitrophenoxy)-1*H*-indole (**11**) - ^{13}C NMR

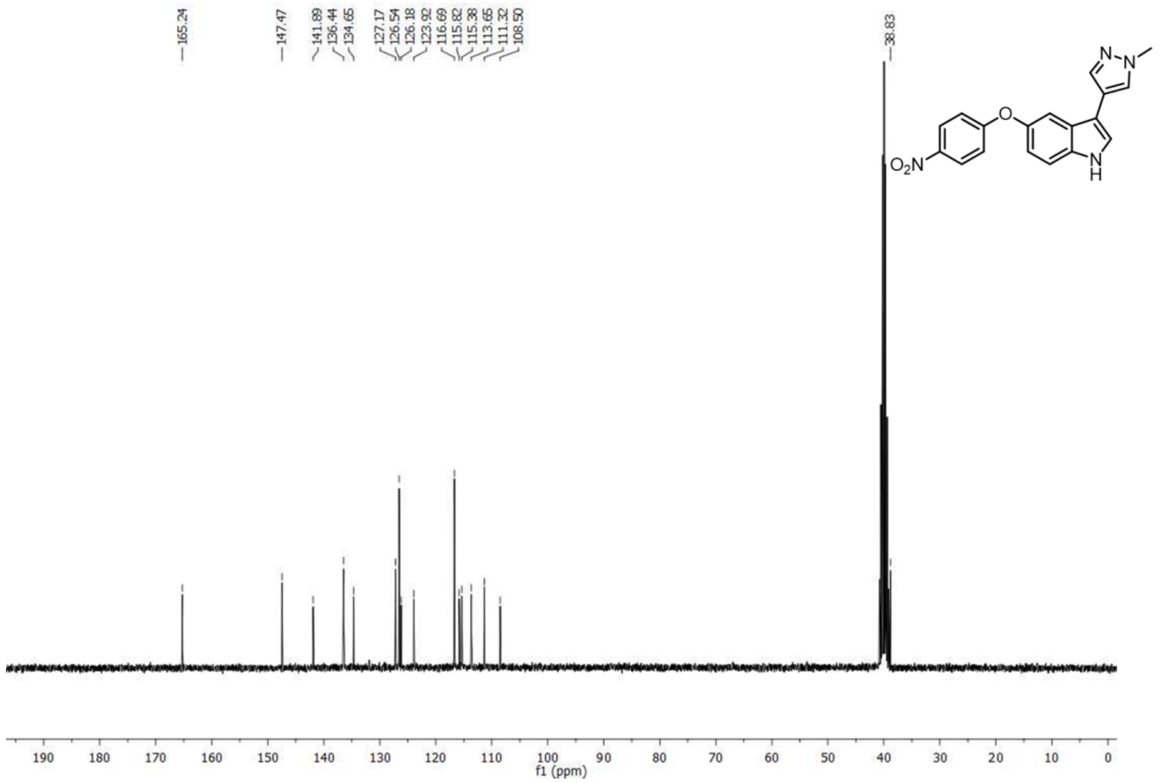

4-((3-(1-Methyl-1*H*-pyrazol-4-yl)-1*H*-indol-5-yl)oxy)aniline (**12**) – ^{1}H NMR

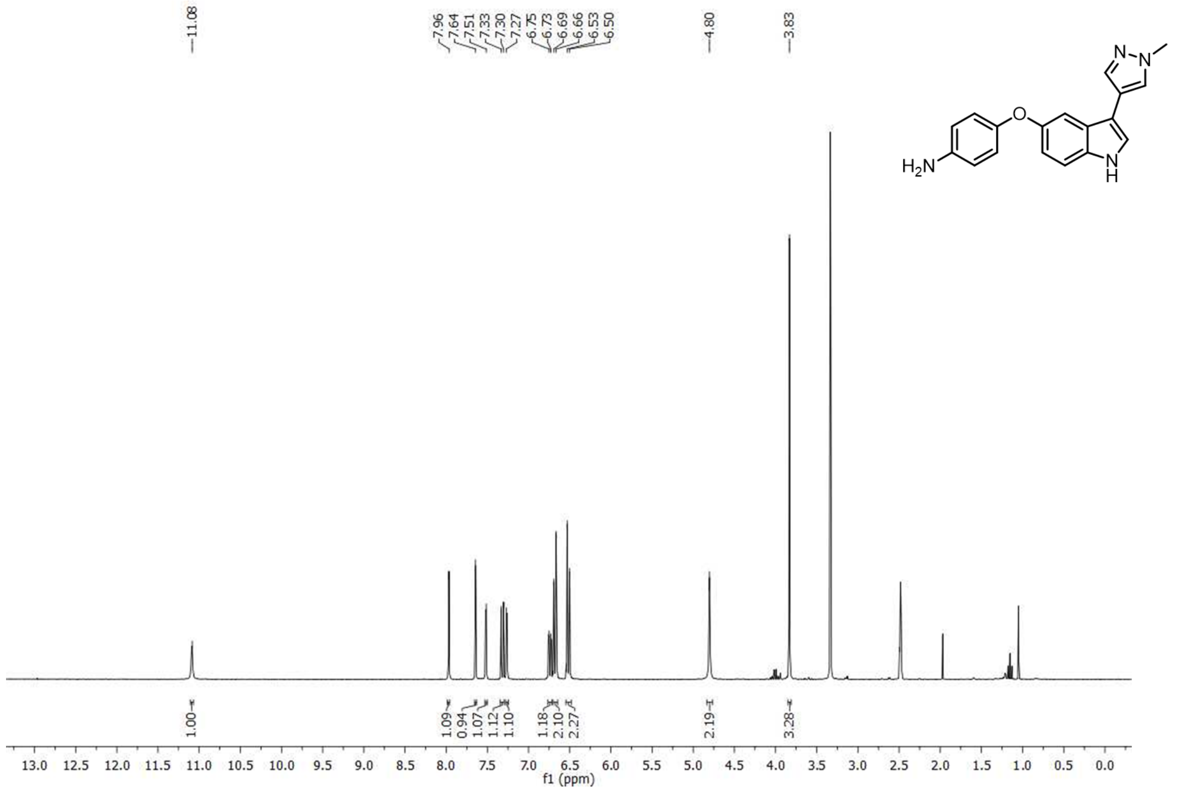

4-((3-(1-Methyl-1*H*-pyrazol-4-yl)-1*H*-indol-5-yl)oxy)aniline (**12**) - ^{13}C NMR

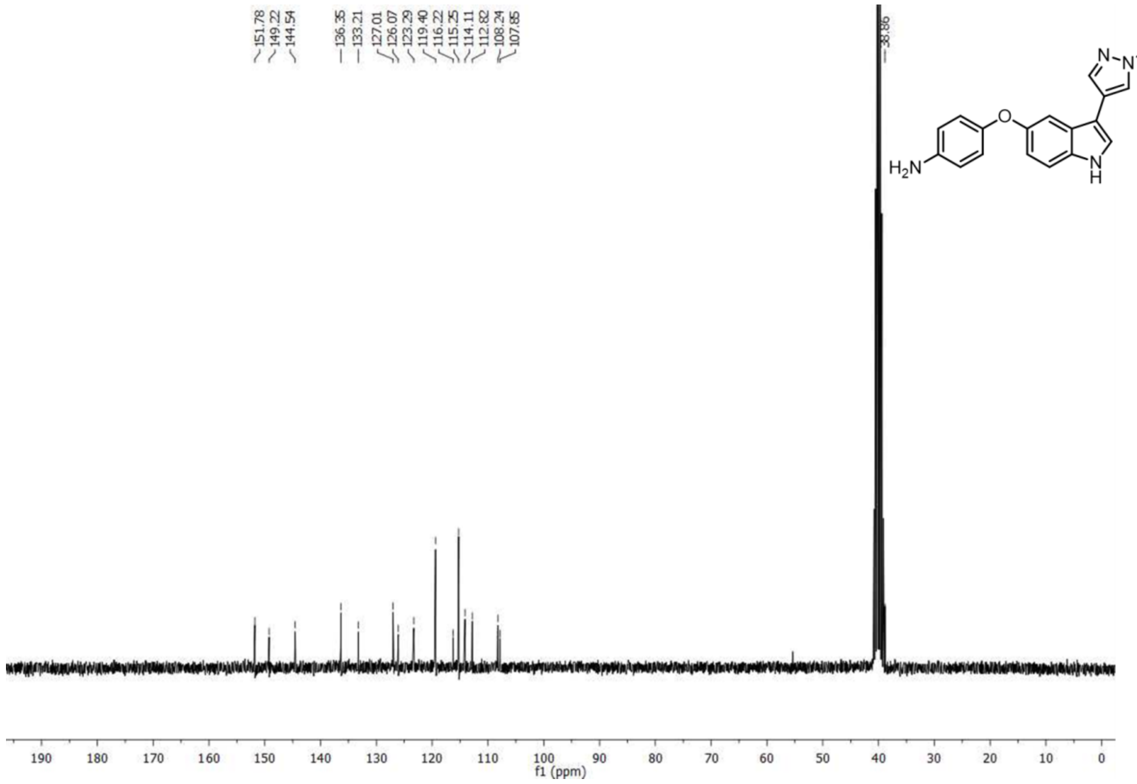

Tert-butyl 2-((4-((3-(1-methyl-1*H*-pyrazol-4-yl)-1*H*-indol-5- yl)oxy)phenyl)carbamoyl)pyrrolidine-1-carboxylate (**13**) – ^{1}H NMR

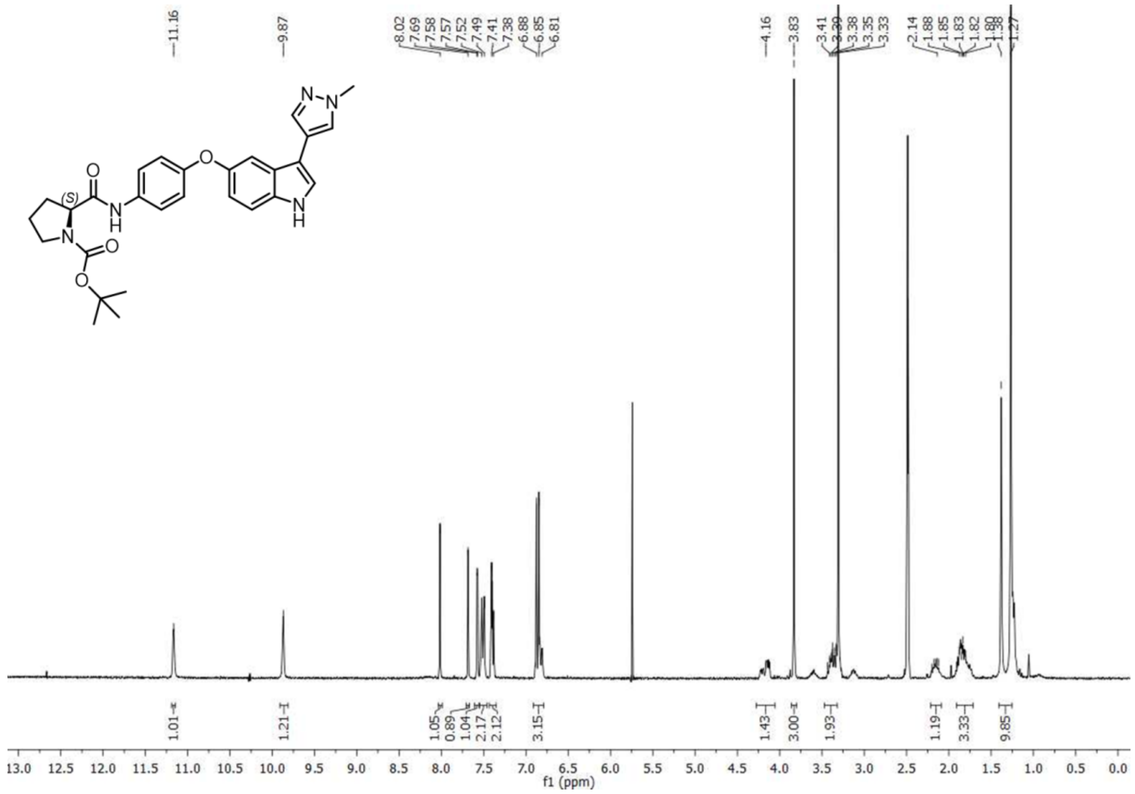

Tert-butyl-(*S*)-2-((4-((1-acryloyl-3-(1-methyl-1*H*-pyrazol-4-yl)-1*H*-indol-5-yl)oxy)phenyl)carbamoyl)pyrrolidine-1-carboxylate) (**14**) – ^{1}H NMR

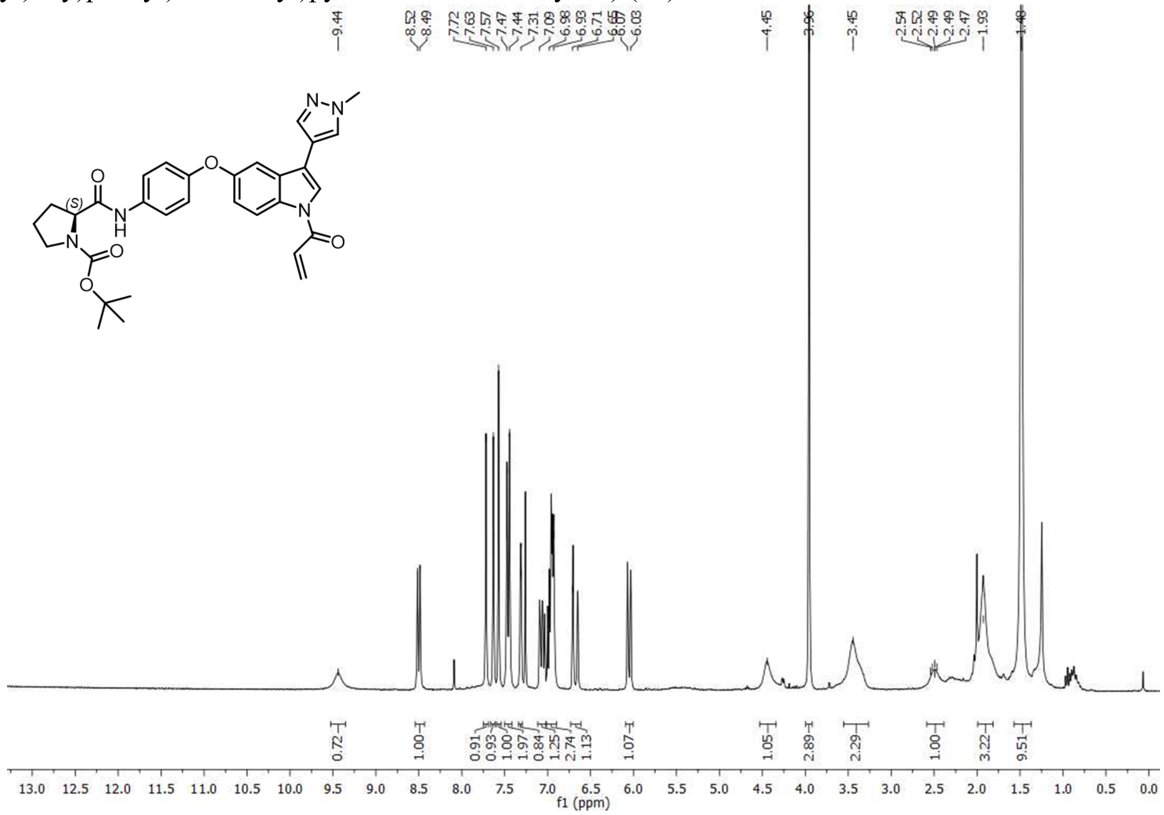

Tert-butyl-(*S*,*E*)-2-((4-((1-(but-2-enoyl)-3-(1-methyl-1*H*-pyrazol-4-yl)-1*H*-indol-5-yl)oxy)phenyl)carbamoyl)pyrrolidine-1-carboxylate (**15**) – ^{1}H NMR

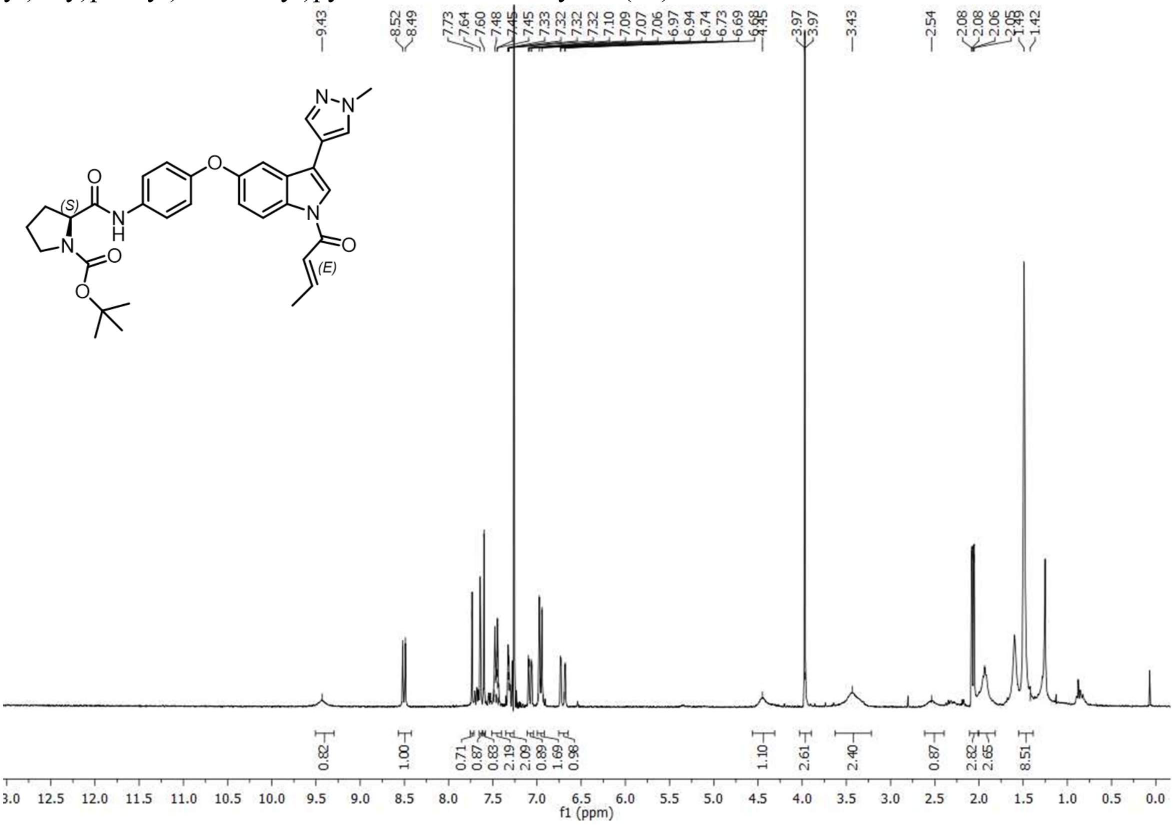

Tert-butyl-(*S*,*E*)-2-((4-((1-(but-2-enoyl)-3-(1-methyl-1*H*-pyrazol-4-yl)-1*H*-indol-5-yl)oxy)phenyl)carbamoyl)pyrrolidine-1-carboxylate (**15**) - ^{13}C NMR

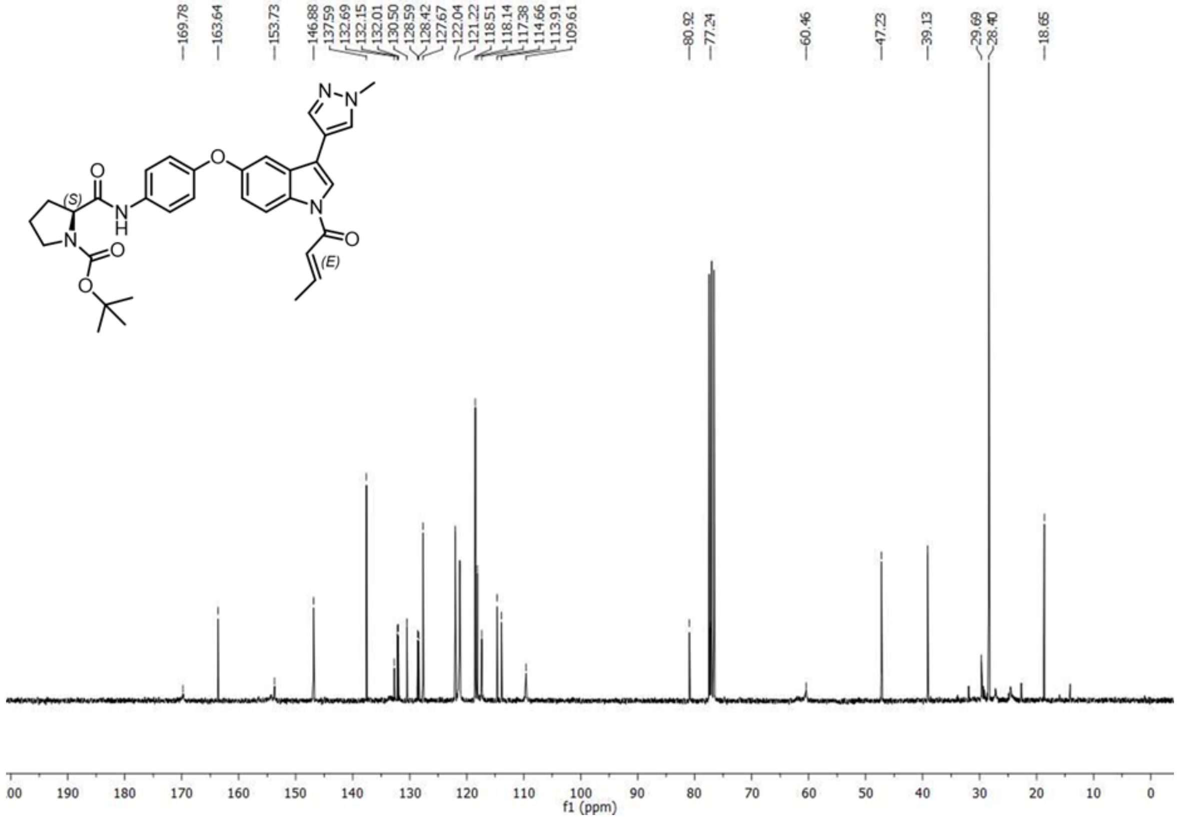

Tert-butyl-(*S*)-2-((4-((1-(3-bromopropanoyl)-3-(1-methyl-1*H*-pyrazol-4-yl)-1*H*-indol-5-yl)oxy)phenyl)carbamoyl)pyrrolidine-1-carboxylate (**16**) – ^{1}H NMR

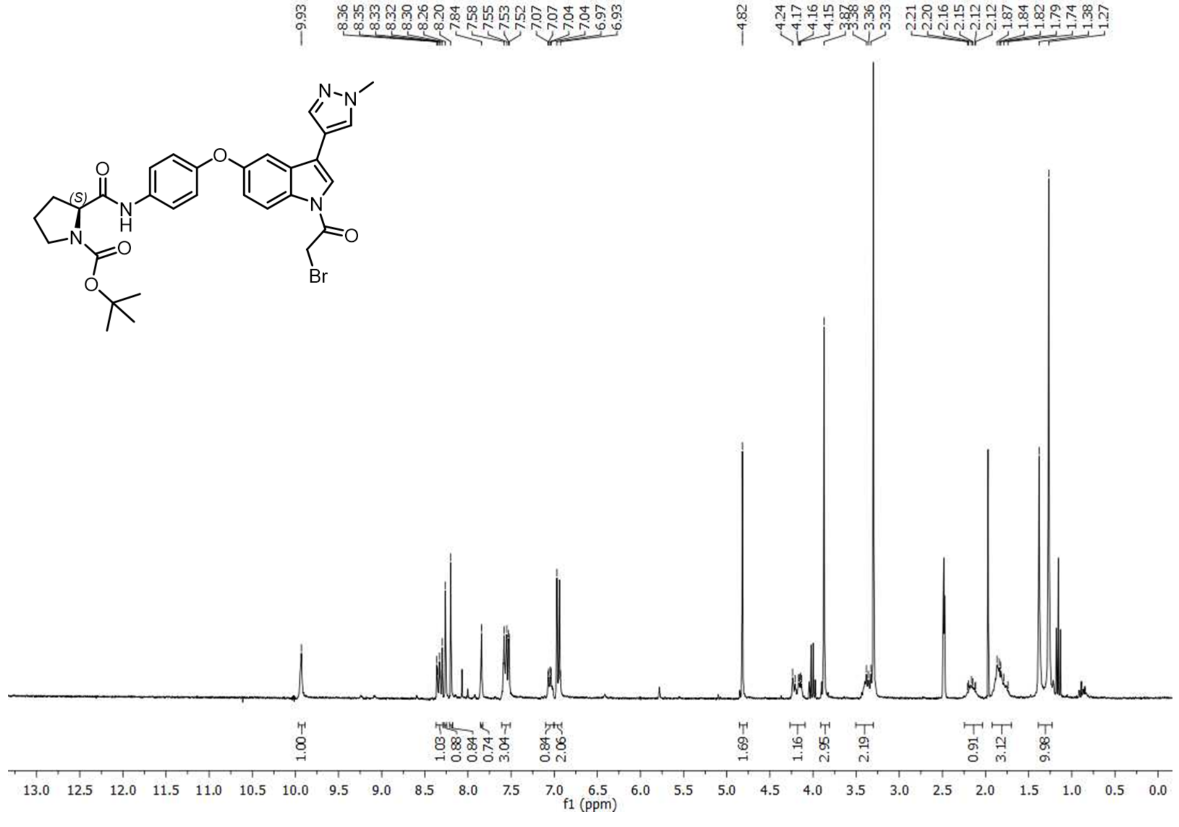

Tert-butyl-(*S*)-2-((4-((3-(1-methyl-1*H*-pyrazol-4-yl)-1-propionyl-1*H*-indol-5-yl)oxy)phenyl)carbamoyl)pyrrolidine-1-carboxylate (**17**) – ^{1}H NMR

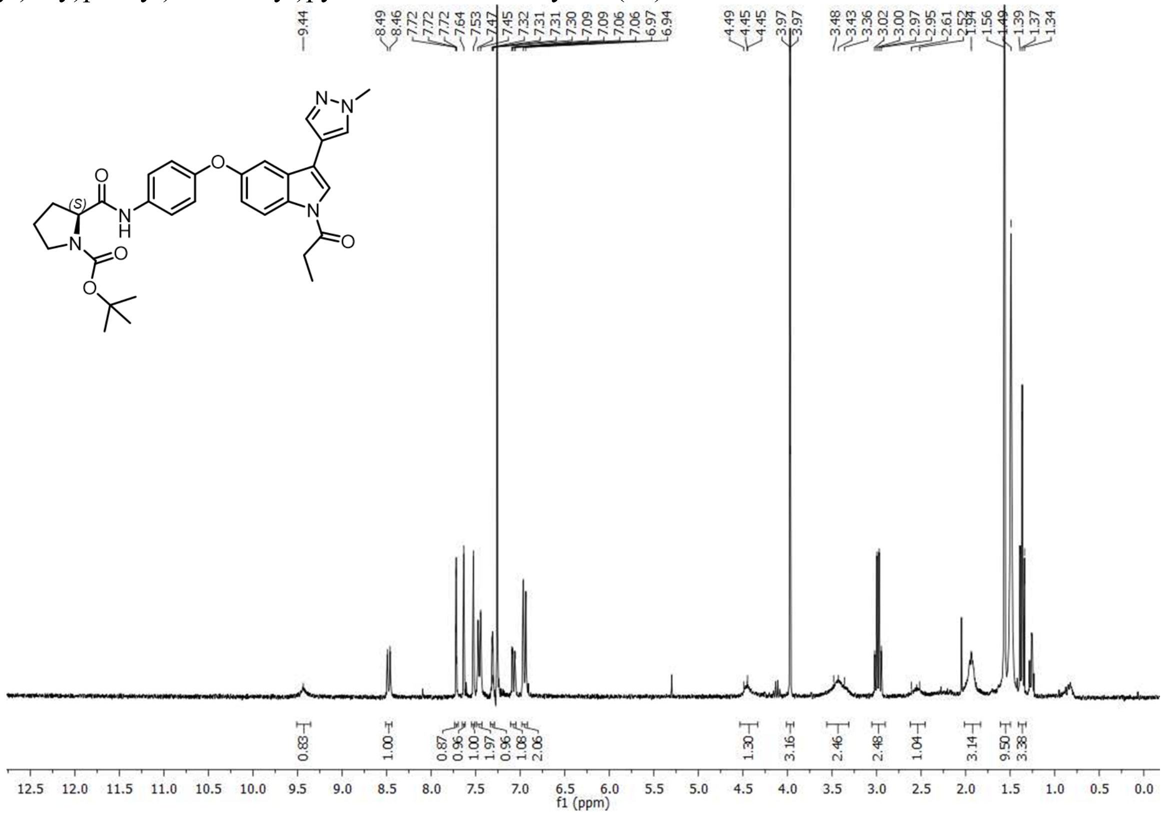

Tert-butyl-(*S*)-2-((4-((3-(1-methyl-1*H*-pyrazol-4-yl)-1-propionyl-1*H*-indol-5-yl)oxy)phenyl)carbamoyl)pyrrolidine-1-carboxylate (**17**) - ^{13}C NMR

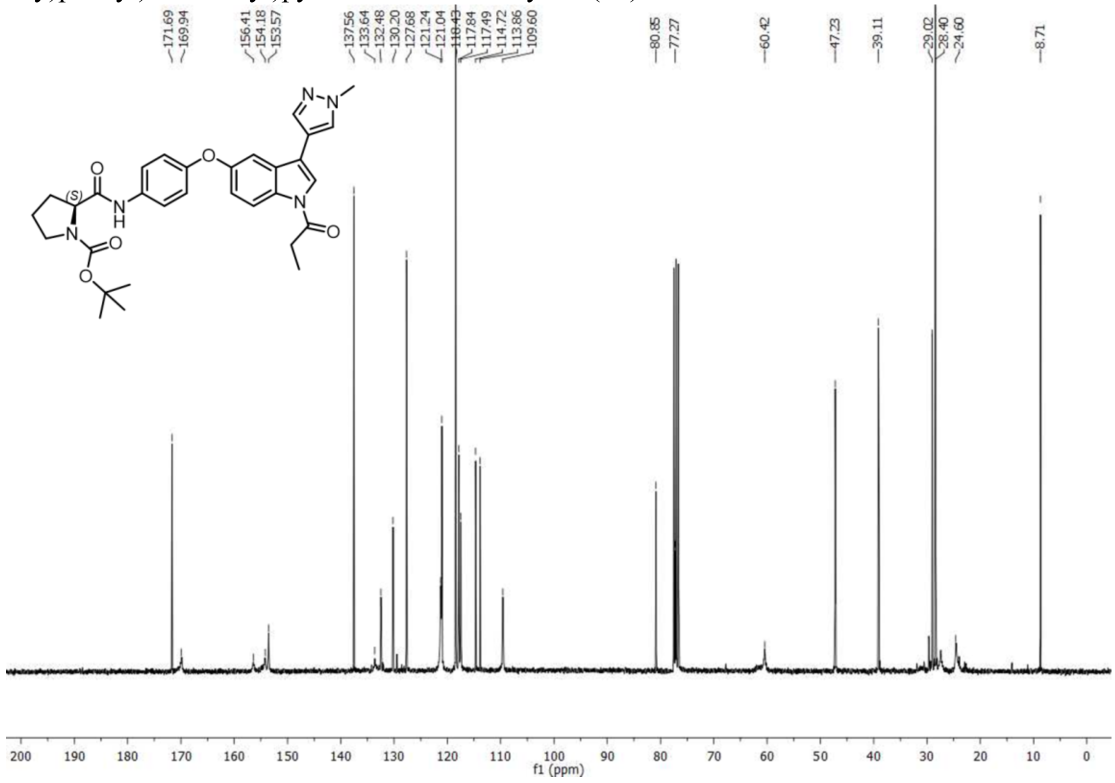

(*S*)-*N*-(4-((1-acryloyl-3-(1-methyl-1*H*-pyrazol-4-yl)-1*H*-indol-5-yl)oxy)phenyl)pyrrolidine-2-carboxamide hydrochloride (**5**) – ^{1}H NMR

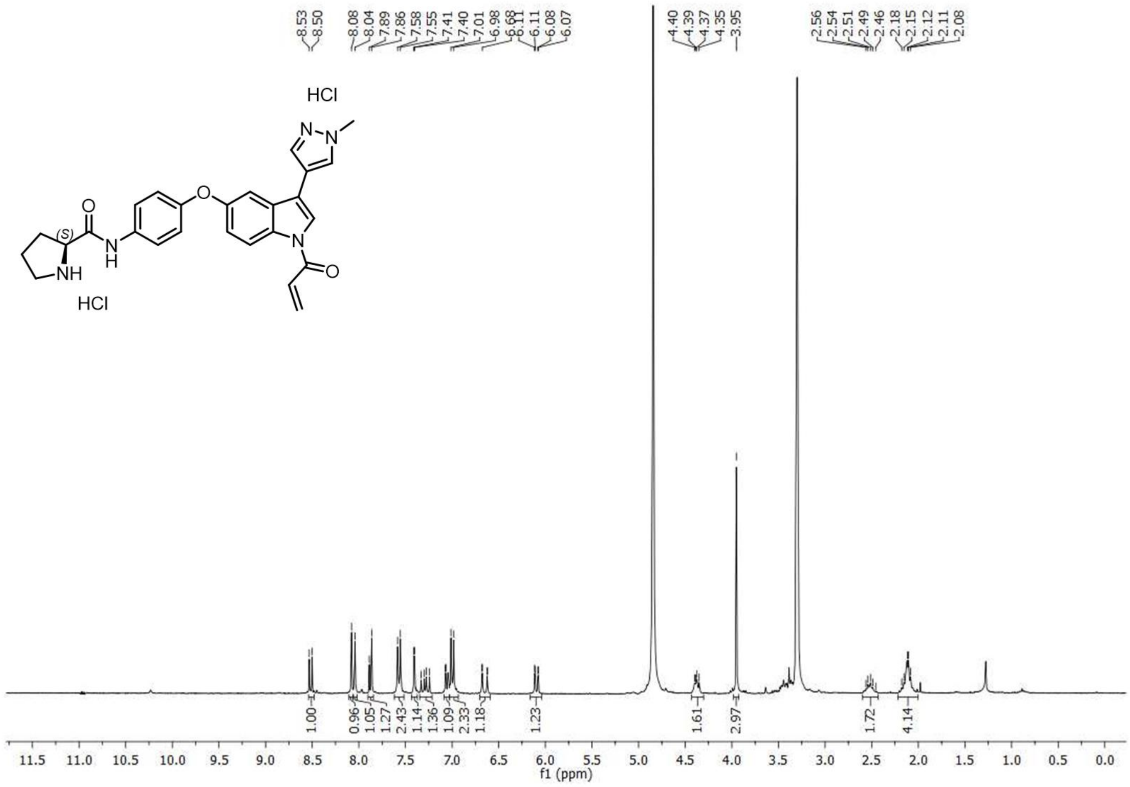

(*S*)-*N*-(4-((1-acryloyl-3-(1-methyl-1*H*-pyrazol-4-yl)-1*H*-indol-5-yl)oxy)phenyl)pyrrolidine-2-carboxamide hydrochloride (**5**) - ^{13}C NMR

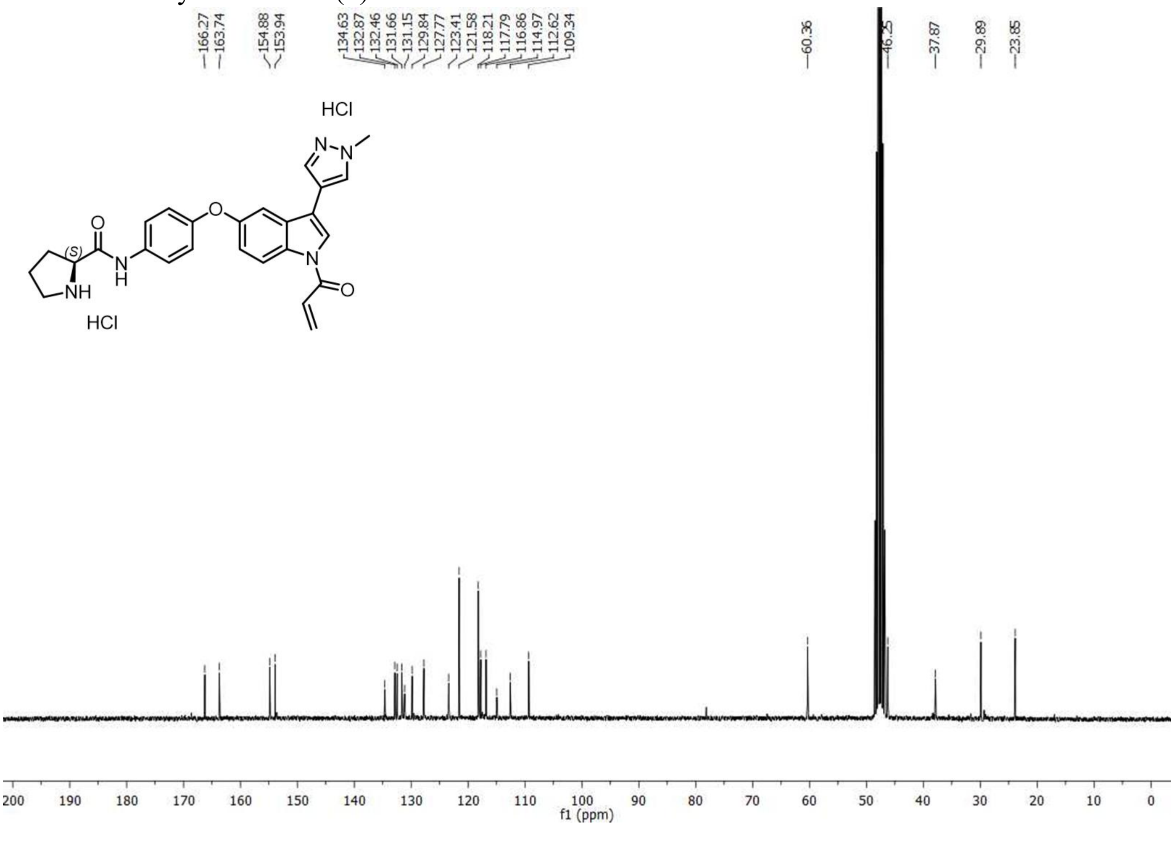

(*S,E*)-1-(3-(1-Methyl-1*H*-pyrazol-4-yl)-5-(4-((pyrrolidin-2-ylmethyl)amino)phenoxy)-1*H*-indol-1-yl)but-2-en-1-one hydrochloride (**6**) – ^{1}H NMR

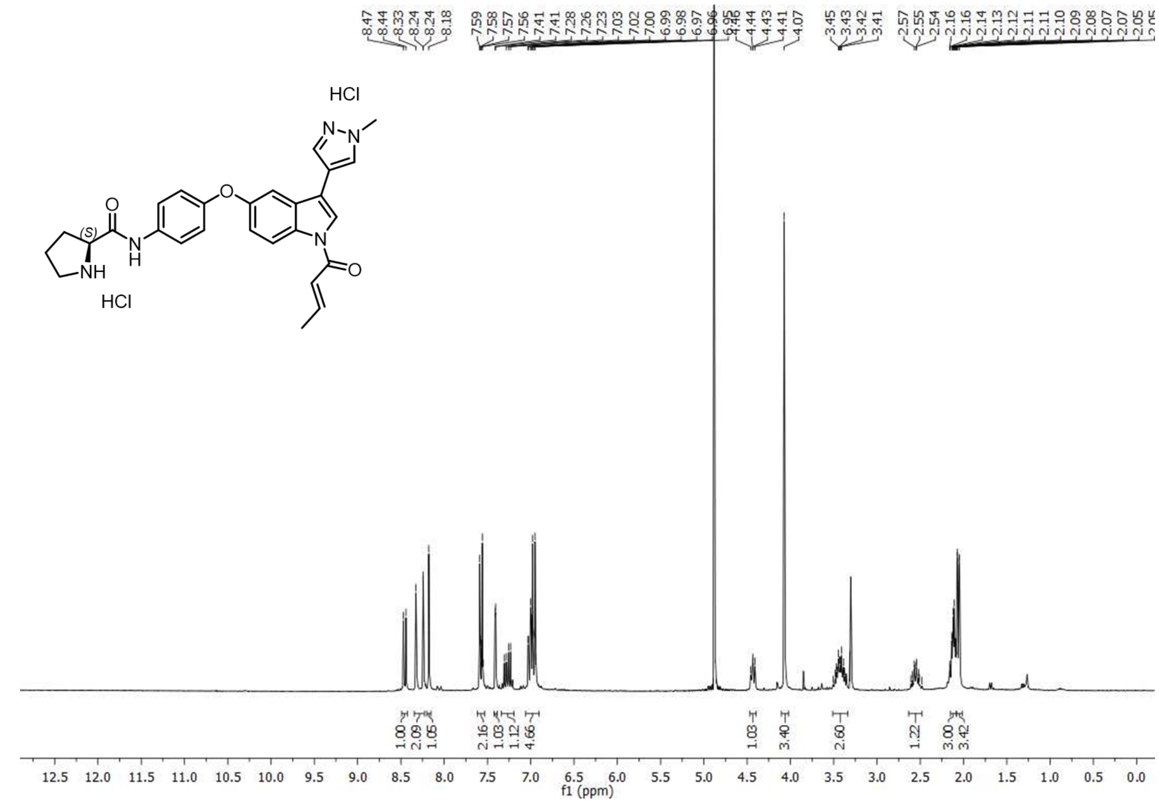

(*S,E*)-1-(3-(1-Methyl-1*H*-pyrazol-4-yl)-5-(4-((pyrrolidin-2-ylmethyl)amino)phenoxy)-1*H*-indol-1-yl)but-2-en-1-one hydrochloride (**6**) - ^{13}C NMR

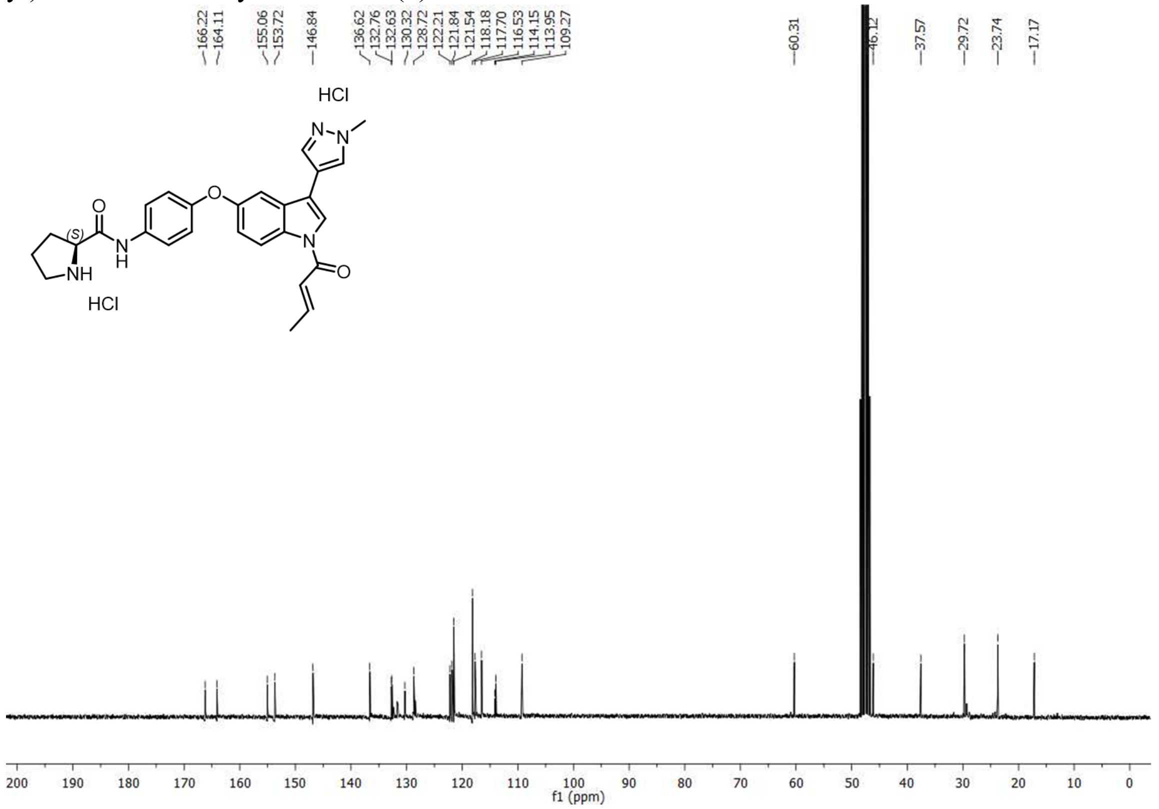

(*S*)-*N*-(4-((1-(3-chloropropanoyl)-3-(1-methyl-1*H*-pyrazol-4-yl)-1*H*-indol-5-yl)oxy)phenyl)pyrrolidine-2-carboxamide hydrochloride (**7**) – ^{1}H NMR

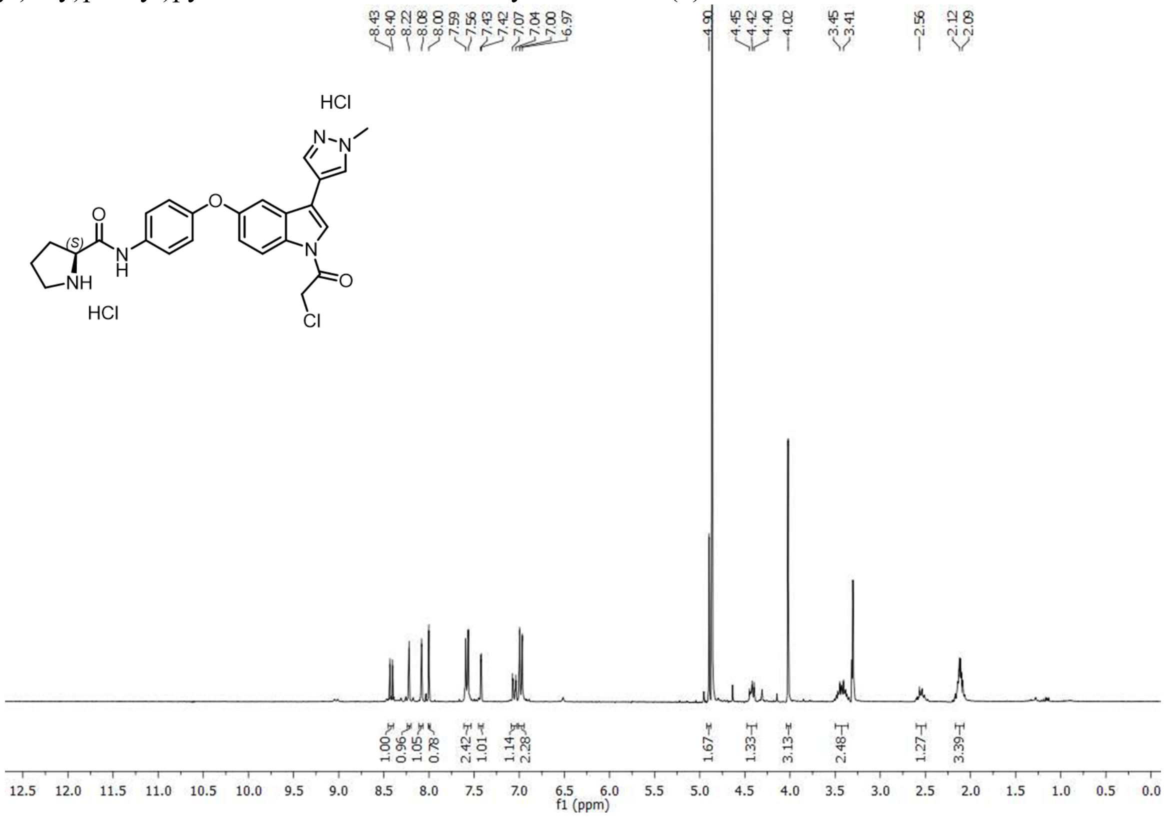

(*S*)-*N*-(4-((1-(3-chloropropanoyl)-3-(1-methyl-1*H*-pyrazol-4-yl)-1*H*-indol-5-yl)oxy)phenyl)pyrrolidine-2-carboxamide hydrochloride (**7**) - ^{13}C NMR

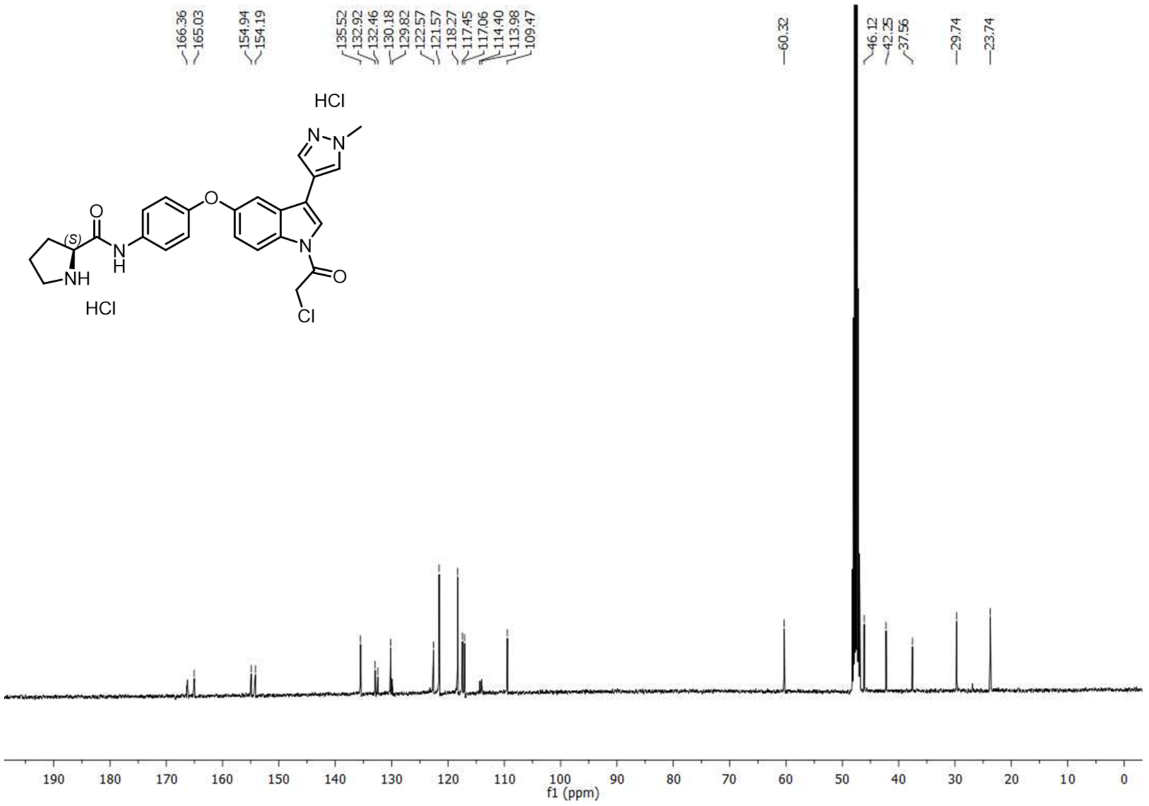

(*S*)-*N*-(4-((3-(1-Methyl-1*H*-pyrazol-4-yl)-1-propionyl-1*H*-indol-5-yl)oxy)phenyl)pyrrolidine-2-carboxamide hydrochloride (**5r**) – ^{1}H NMR

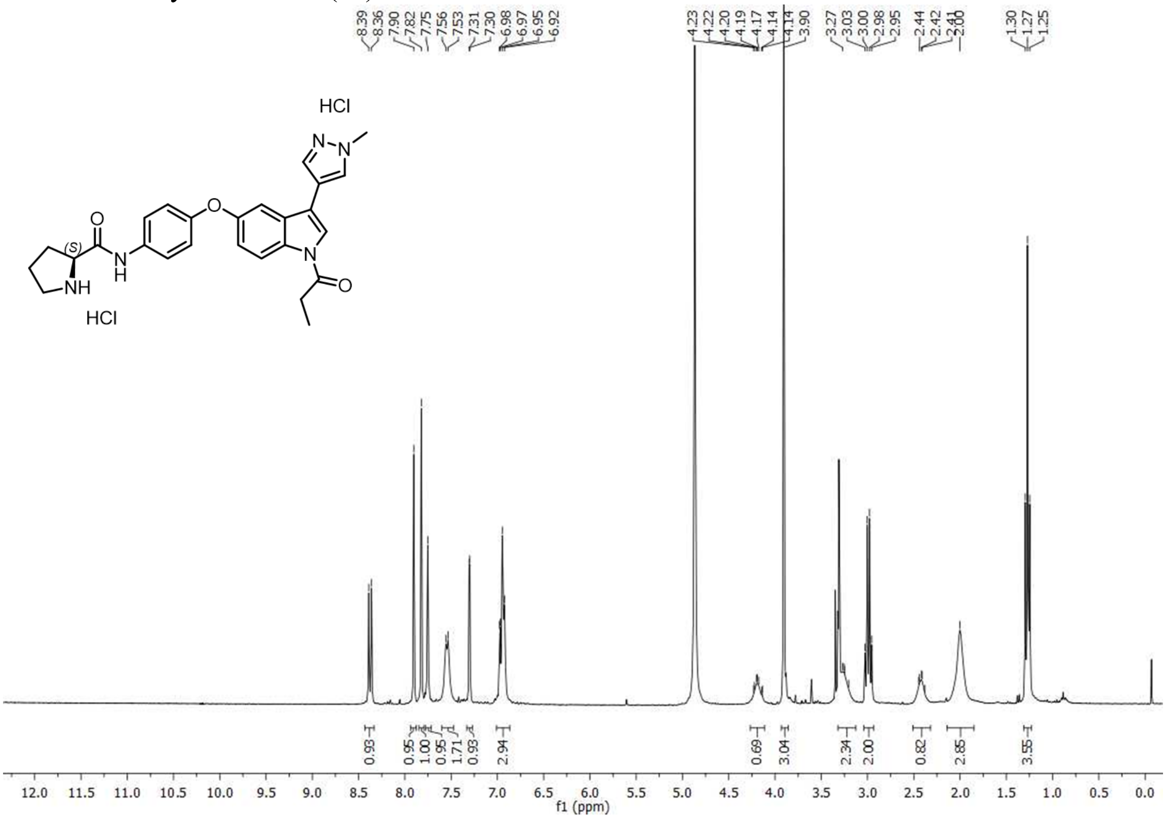

(*S*)-*N*-(4-((3-(1-Methyl-1*H*-pyrazol-4-yl)-1-propionyl-1*H*-indol-5-yl)oxy)phenyl)pyrrolidine-2-carboxamide hydrochloride (**5r**) - ^{13}C NMR

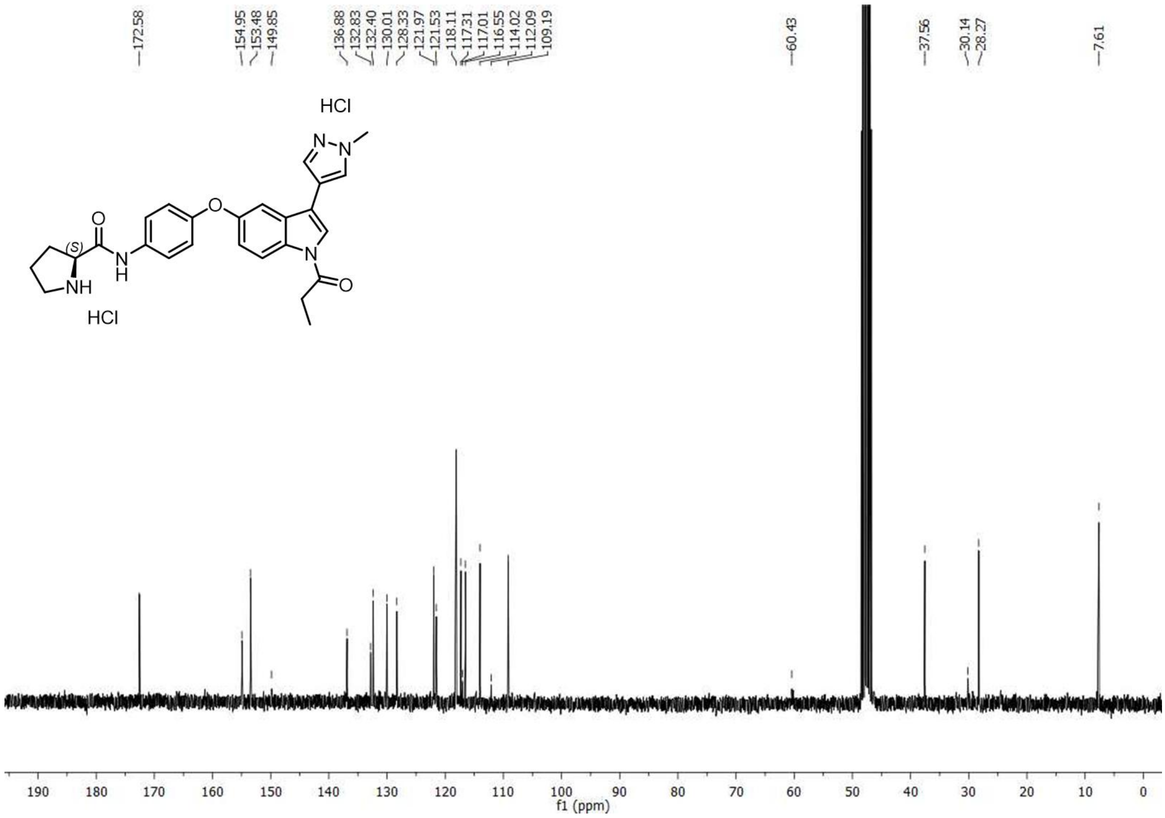

HPLC Chromatograms for Compounds 5-7 and 5r

(*S*)-*N*-(4-((1-acryloyl-3-(1-methyl-1*H*-pyrazol-4-yl)-1*H*-indol-5-yl)oxy)phenyl)pyrrolidine-2-carboxamide hydrochloride (**5**)

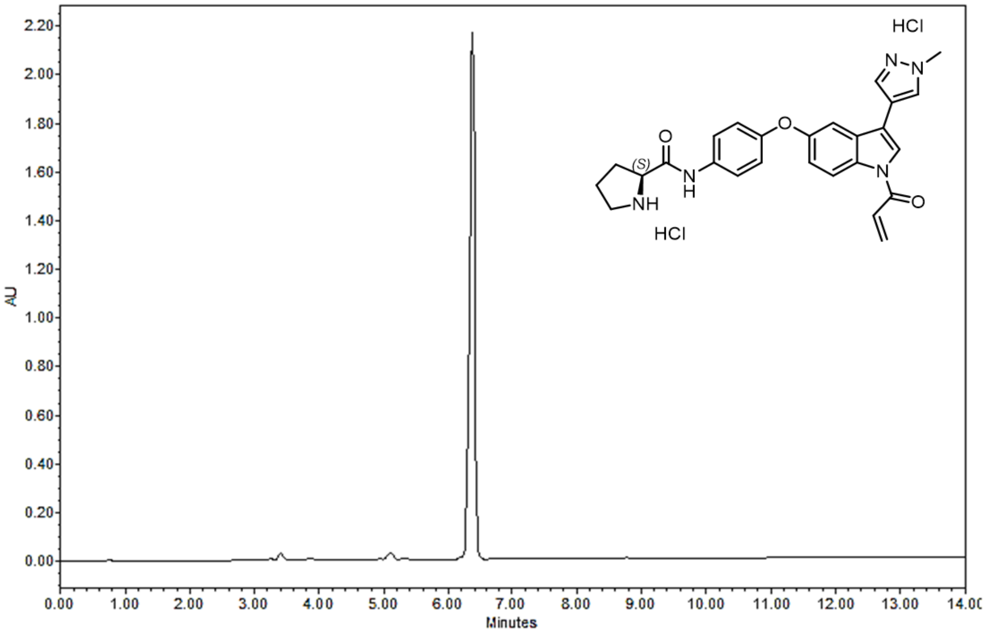

Retention time (min)	% Area
6.366	96.40%

(*S,E*)-1-(3-(1-Methyl-1*H*-pyrazol-4-yl)-5-(4-((pyrrolidin-2-ylmethyl)amino)phenoxy)-1*H*-indol-1-yl)but-2-en-1-one hydrochloride (**6**)

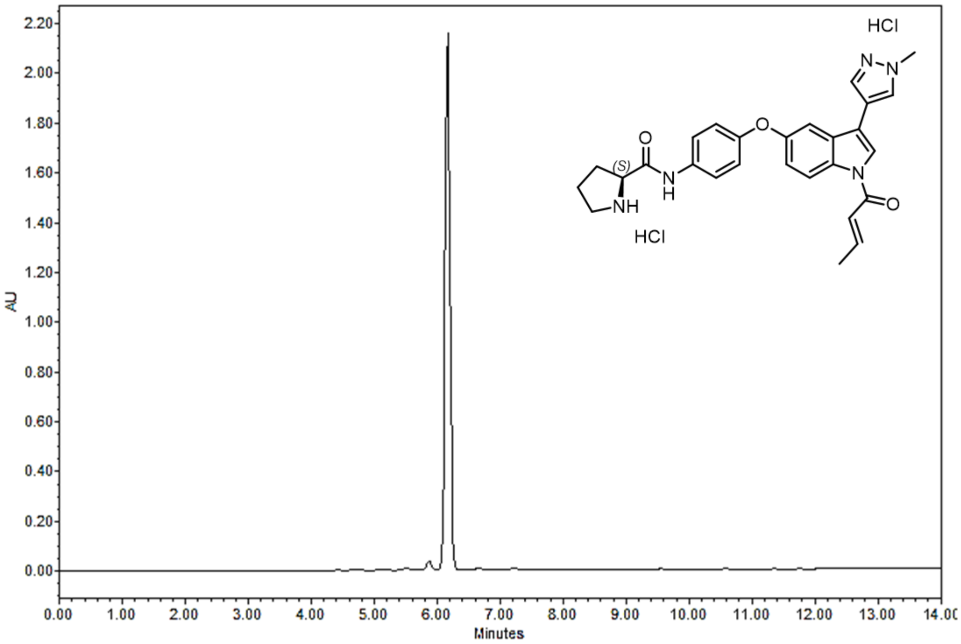

Retention time (min)	% Area
6.163	96.15%

(*S*)-*N*-(4-((1-(3-chloropropanoyl)-3-(1-methyl-1*H*-pyrazol-4-yl)-1*H*-indol-5-yl)oxy)phenyl)pyrrolidine-2-carboxamide hydrochloride (**7**)

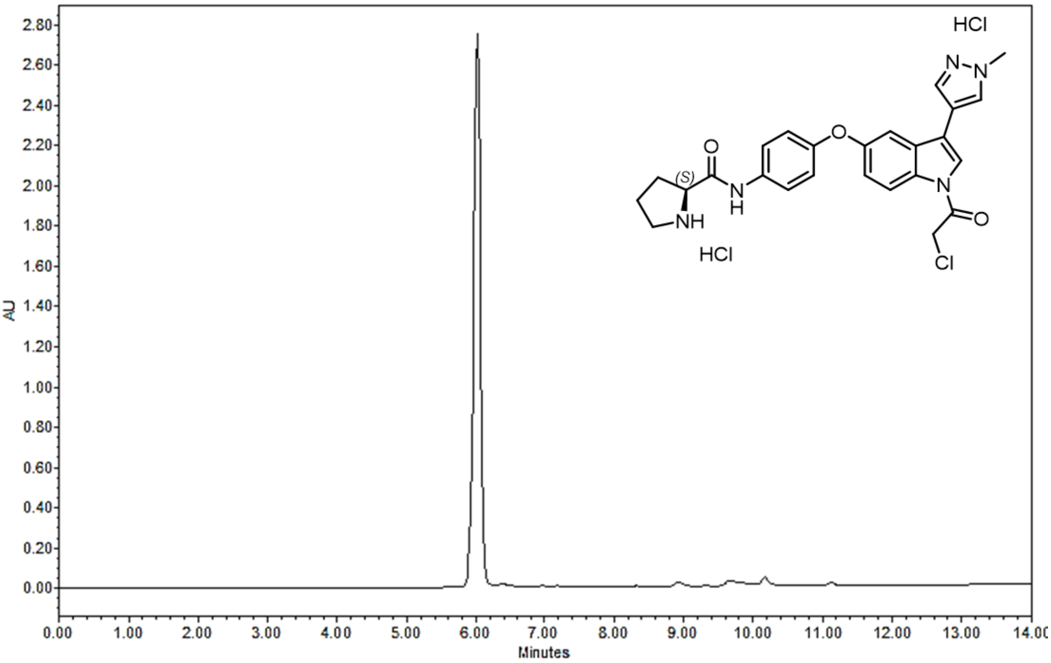

Retention time (min)	% Area
6.014	97.30%

(*S*)-*N*-(4-((3-(1-Methyl-1*H*-pyrazol-4-yl)-1-propionyl-1*H*-indol-5-yl)oxy)phenyl)pyrrolidine-2-carboxamide hydrochloride (**5r**)

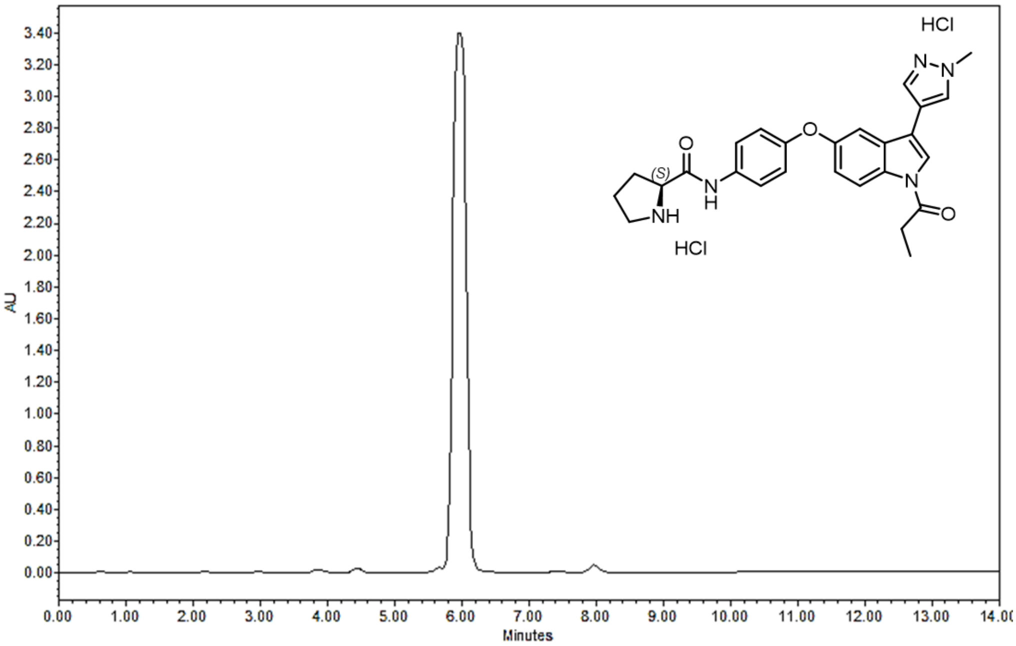

Retention time (min)	% Area
5.956	96.45%
